# The development of a brief version of the Lexington Attachment to Pets Scale (Brief-LAPS)

**DOI:** 10.3389/fvets.2025.1619187

**Published:** 2025-09-02

**Authors:** Thomas Bøker Lund, Sandra A. Corr, Viktoria Hirschhofer, Peter Sandøe, James Serpell, Svenja Springer

**Affiliations:** ^1^Department of Food and Resource Economics, University of Copenhagen, Copenhagen, Denmark; ^2^College of Medical, Veterinary and Life Sciences, University of Glasgow, Glasgow, United Kingdom; ^3^Department of Interdisciplinary Life Sciences, Messerli Research Institute, University of Veterinary Medicine Vienna, Vienna, Austria; ^4^Department of Veterinary and Animal Sciences, University of Copenhagen, Frederiksberg, Denmark; ^5^School of Veterinary Medicine, University of Pennsylvania, Philadelphia, PA, United States

**Keywords:** Lexington Attachment to Pets, Brief-LAPS, Brief-Lexington Attachment to Pets, companion animal attachment, human-animal interaction (HAI), companion animal interaction, human animal attachment

## Abstract

Several questionnaire-based instruments have been developed to measure pet owners' attachments to their pets, but they are often lengthy, which likely limits their use in studies where respondent fatigue and data collection costs are concerns. One of these is the Lexington Attachment to Pets Scale (LAPS) which has been increasingly used in recent years. It consists of 23 items addressing owners' levels of attachment to their pets. A total attachment score (full LAPS), and 3 sub-scores on the sub-dimensions of General Attachment, People Substitution, Animal Rights/Welfare, can be calculated. The current paper describes the development of a brief-version of the LAPS (Brief-LAPS). We first provide an overview of existing research where the LAPS is used. Then, to develop the Brief-LAPS, we use a combination of input from experts in human-animal interaction (*n* = 54) about the content validity of the 23 items, and analysis of measurement invariance on questionnaire data from cat and dog owners in 3 European countries [Austria, Denmark, and the UK (*n* = 2,037)]. Sixteen of the 23 original items were removed, leaving a 7-item Brief-LAPS scale where items from all 3 sub-dimensions are represented. The Brief-LAPS is intended to replace the full LAPS, and it does not offer brief versions of the 3 sub-dimensions. The full and Brief-LAPS are highly correlated (Pearson's r = 0.95). Also, patterns of associations between the full LAPS and a range of measures of interest in practical research are highly similar when running the same associational analyses with the Brief-LAPS. For future users, the paper provides scoring instructions for the Brief-LAPS, including how to calculate a composite score (range: 0–21). We recommend the use of the Brief-LAPS for studies in which questionnaire burden and data collection costs are a concern, but not when mean comparisons with the full LAPS are of interest, or when one or more of the LAPS sub-dimensions are of interest. Scholars should use caution when comparing the Brief-LAPS scores between countries. We recommend additional psychometric evaluations particularly in non-Western countries, to ensure that the Brief-LAPS scale is psychometrically sound beyond the three countries studied in this paper.

## Introduction

A central focus of the rapidly growing research on human-animal interaction (HAI) considers attachment to companion animals. The quality or strength of this attachment is often assessed through questionnaires, and frequently the measures of attachment come in the form of latent scales where multiple question items are used. Many latent scales of companion animal attachment have been developed over the years. Limiting our overview to measures where companion animal attachment in adults can be assessed for more than one animal species, the first appeared in the mid-1980′s (1). In subsequent years, additional measures were developed (2–8) and recently two further measures have been added (9, 10).

Most of these existing attachment measures require the participants to respond to a relatively large number of questionnaire items, i.e., often 15 or more questions (see Table 1). Arguments for using multiple question items include the ability to uncover more facets of the attachment to ensure content coverage, and that using more items will increase the variance of the latent scale. Indeed, the Cronbach's α equation, which is a widely used coefficient of reliability/internal consistency, rewards scales with many items (11).

**Table 1:** An overview of instruments that assess human-animal attachment (across species and in adult populations).

**Source**	**Measure (possible sub-dimensions)**	**Number of question items**
Albert and Bulcroft (2)	Pet Attachment Scale	9
Bures et al. (9)	Shortened version of the Pet Attachment (PAS)	3 (for Primary Caregiver)
Holcomb et al. (1)	Pet Attachment Survey	27 (16 for “Relationship Maintenance Scale”, 11 for “Intimacy scale”)
Johnson et al. (3)	Lexington Attachment to Pets Scale (“General Attachment”, “People Substitution”, “Animal Rights/Welfare”)	23 (11 for “General Attachment”, 7 for “People Substitution”, 5 for “Animal Rights/Welfare”)
Kafer et al. (4)	Pet Relationship Scale (PRS) (“Affectionate Companionship (AC)”, “Equal Family Member Status (EFMS)”, “Mutual Physical Activity (MPA)”)	22 (8 for “Affectionate Companionship (AC)”, 7 for “Equal Family Member Status (EFMS)”, and 7 for “Mutual Physical Activity (MPA)”)
Nugent and Daugherty (10)	Family Bondedness Scale	23
Winefield et al. (6)	The Owner-Pet Relationship (OPR)	15
Zasloff (7)	Comfort from Companion Animal Scale	11
Zilcha-Mano et al. (8)	Pet Attachment Questionnaire (Attachment anxiety scale, attachment avoidance scale)	26 (13 for each scale)

We only include scales where companion animal attachment in adults can be assessed for more than one animal species (i.e. at least cats and dogs). This excludes papers where several items of interest were reported but no scales were developed [e.g.: (65)], species-specific measures of attachment (66) and children's pet attachment (67).

In some data collection settings, measures that require many questions may be unproblematic. However, questionnaire length may pose a real challenge in population studies where random sampling of populations is used. Here, survey bureaus are often contracted to collect the data, and survey cost charges increase in tandem with the length of the questionnaire. Further, when random sampling is used, it is central to obtain a high response rate to avoid nonresponse bias. So questionnaires should be as brief as possible to limit respondent burden (12) that can cause respondents to drop out before completing the questionnaire (13, 14) or to decline to respond at all when the estimated completion time is presented in the invitation text (15).

There is therefore a need to provide researchers who collect data where respondent burden and data collection costs are a concern with a measure of companion animal attachment that is relatively brief, but still psychometrically sound, and where the variance of the latent scale is not sacrificed.

The aim of this paper is to develop a brief version of an existing human-animal attachment scale, namely the Lexington Attachment to Pets Scale (LAPS) (3). The LAPS consists of 23 items which are intended to assess attachment levels of humans for companion animals across animal species. A total attachment score can be calculated (we will refer to this as the full LAPS), but it is also possible to focus on 3 sub-dimensions of attachment which the scale developers labeled General Attachment, People Substitution, and Animal Rights/Welfare (3). Here, we only aim to develop a brief version of the full LAPS scale, which we will refer to as the “Brief-LAPS”.

Our motivation for developing a brief version of an existing measure instead of generating an entirely new one stems from a desire to create a measure that also is *backward compatible* with existing studies and findings. We see two good reasons for focusing on the LAPS. First, it appears to be one of the most widely used scales of its type. Second, as we show in Study 1 below, the use of the LAPS has grown steadily in recent years. Indeed, validation studies where the LAPS is translated into country-specific versions are continuously appearing (16–19). Concurrently however, there are indications that the factorial structure of the original LAPS suggested by Johnson and colleagues cannot be replicated (16, 17, 20). This challenge could be addressed by developing a brief version of the LAPS using techniques designed to support psychometric scale development.

We divided the research into four separate studies that each contributed to the development of the Brief-LAPS. Study 1 is a literature review focusing on how the LAPS has been used in applied research. The review includes information about how the LAPS score variable is constructed in practical research, and the types of research themes for which the LAPS is used. In Study 2, results are presented from a survey of HAI experts that were tasked with evaluating the content validity of the 23 LAPS items. Their evaluations are used in the selection of items to be included in the Brief-LAPS. In Study 3, the Brief-LAPS is developed and psychometrically assessed using the method of measurement invariance, and the 7 items that make up the resulting Brief-LAPS are presented. In Study 4, we compare whether results using the full LAPS scale and the Brief-LAPS scale produce similar findings when it comes to statistical significance testing of associations between LAPS and other measures of interest to the research community.

## Study 1: Review of the use of the LAPS in the research literature

### Study 1: introduction

The Brief-LAPS should be relevant and easily applicable for researchers studying attachment to companion animals. We therefore initially focus on how the full LAPS has been used in practical research, including an overview of the species in which the owner-animal attachment has been studied, and the type of research in which the LAPS has previously been used. We also focus on how the LAPS variable has been calculated in practical research. The reason for this is that there are different ways in which the scores on latent scales, such as the LAPS, can be calculated. These include the composite score method (i.e. all observed item scores are summed together), and, factor scores, which are linear combinations of the observed items (21). Johnson and colleagues used the composite approach: specifically, they developed a composite variable for the full LAPS scale ranging from 0 (lowest level of attachment) to 69 (highest level) (3).

### Study 1: methods and analysis

A literature search was conducted in four electronic databases: Scopus, BASE, Web of Science, and Google Scholar. The search query used for all databases was “Lexington Attachment to Pets Scale”. Initially, the frequently used abbreviation “LAPS” was also used as a search term, but this was abandoned, as it generated a very large number of hits, because LAPS is an abbreviation and concept that is used in other research fields. Since all manuscripts included in the review mention the full name at least once before using the abbreviation, we believe that we missed very few, if any, papers.

Initial searches in Scopus, BASE, and Web of Science (January 2024) focused on titles, keywords, and abstracts. In February 2024, Google Scholar was used, as this electronic database also included full-text searches. Duplicate records were excluded, i.e., a publication identified in one database was not included again if found in another. Author S.S. reviewed the initial results from Scopus, BASE, and Web of Science, while author V.H. reviewed the Google Scholar results. Publications were included when they met the following criteria: (i) research reported in a peer-reviewed journal, (ii) in English language, (iii) the LAPS questionnaire data were used and reported in the article. Publications were coded by S.S. and V.H. to extract information about journal, study country, studied animal(s), approach to calculating LAPS scores, types of analyses performed, and the overarching research themes of the manuscripts (for more details about the literature search, see Supplementary File 1).

We present the results of the literature review using cross-tabulations with publication counts, and depict temporal trends in the use of the full LAPS scale and its three sub-dimensions using a line graph.

### Study 1: results

**Figure 1:**
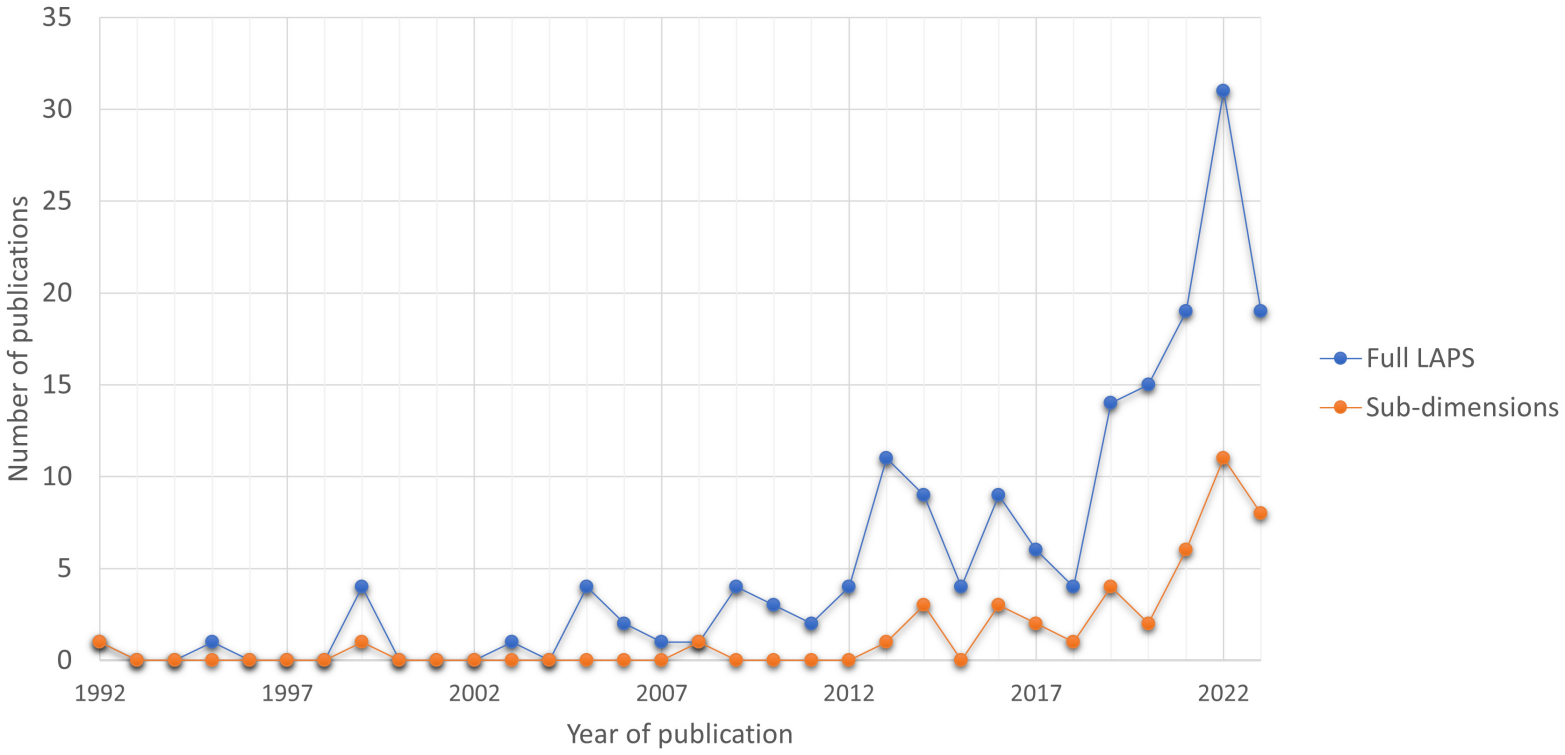
Use of the full LAPS and the 3 LAPS sub-dimensions in English peer-reviewed papers in the period 1992 to 2023 (*n* = 169). The figure includes publications from the period 1992 to 2023 only. Publications from 2024 were excluded, as the total number of publications for that year was not fully recorded in the databases at the time the literature search was conducted.

One hundred and seventy-five peer-reviewed papers that made use of the scale in the period January 1992 to February 2024 were identified. It can be seen in Figure 1 that use of the LAPS has grown steadily in recent years.

### Overview of journals and study populations

The LAPS was used in research published in the major journals that focus on anthrozoology. The study populations were from varied geographical regions, although most studies featured Western populations. Only 19 of the studies involved owners from Asia or South America (see Table 2).

**Table 2:** Overview of journals where the LAPS measure is used, study countries and pet animal species covered (*n* = 175).

**Journals**	**Count**
Anthrozoös	42
Animals	19
Human-Animal Interactions Bulletin	11
Journal of Applied Animal Welfare Science	7
Others	96
Total	175
**Study country**	
USA	72
UK	13
Italy	11
Canada	12
Germany	10
Australia	7
South America (Brazil, Mexico, Argentina)	9
Asia (China, Japan, Korea)	10
More than one country included	13
Others	18
Total	175
**Animal species**	
Dogs only	66
Dogs and Cats	38
Multiple (>2) animal species	23
Animal species not specified	20
Cats only	18
Dolphins, Fish, zoo animals, reptiles	6
Rabbits only	2
Rats only	1
Horse only	1
Total	175

Sixty-six studies included only dogs, 18 included only cats, and 38 included both species. In 23 studies, multiple animal species were covered, although these studies typically also included dogs, cats or both dogs and cats.

### Use of the full LAPS scale and 3 LAPS sub-dimension scales

Most studies (*n* = 159) conducted descriptive statistics on, or reported the internal consistency of, the full LAPS (see Table 3). In many studies (*n* = 133) the full LAPS was used in associational analysis. Thirty-eight studies reported descriptive statistics for, or the internal consistency of, 1 or more of the 3 sub-dimensions. There were 46 studies that used 1 or more of the sub-dimension scales in associational analyses.

**Table 3:** Overview of the use of the full LAPS scale, and the three LAPS sub-dimension scales (*n* = 175).

**Use of the full LAPS and the three LAPS sub-dimensions**	**Count**
Descriptive statistics or internal consistency reported for the full LAPS scale	159
The full LAPS was used in associational analyses	133
Descriptive statistics or internal consistency reported for one or several sub-dimension scales	38
One or several sub-dimension scales were used in associational analyses	46

### Calculation of LAPS scores, and use of group mean comparisons and correlational analyses

A clear majority of studies (*n* = 127) used the composite approach to calculate LAPS scores. Specifically, they followed the scoring method laid out by Johnson and colleagues in the original paper, which produces a composite variable for the full LAPS scale ranging from 0 (lowest level of attachment) to 69 (highest level) (3).

Just six studies reported the use of factor scores, and 4 studies used a structural equation approach. In 37 of the studies, the scoring approach was not described in the methods section, and it is not possible to infer the approach based on the presented results and analyses. Group comparisons of the means of the LAPS were conducted in 70 of the studies, and correlational tests were carried out in 132 studies (see Table 4).

**Table 4:** Overview of practices to calculate LAPS scores, and use of group mean comparisons and correlational analyses (*n* = 175).

**Calculation of scale scores and types of analysis**	**Count**
**Scale scores employed**	
Composite scores	127
Factor scores	6
Structural equation model	4
Not reported	37
**Types of analysis where LAPS is used**	
Group mean comparison	70
Analyses with correlation coefficients	132

### Research themes for which the LAPS was used

Five overarching research themes were identified. The first theme, human health and conditions, explores how the emotional attachment between owners and their pets affects human mental health (22–25), physical health (26–28), perceived loneliness (12, 29), and lifestyle choices, such as exercising together (30). The second theme, human behavior and attitudes toward pets, involves studies that use the LAPS to investigate how emotional attachment influences owners' behavior toward pets, such as caring for aging animals (31, 32), and spending more time with the animal (30, 33), owners' decision-making about the use of veterinary services (34–36), or owners' willingness to invest in pet products and services (37). The third theme examines emotional attachment as a specific human socio-demographic characteristic (24, 38, 39). The fourth theme focuses on human personality, investigating how traits like neuroticism impact human-animal attachment (40). Finally, the fifth theme uses the LAPS to compare emotional attachment to different animal species, such as the differences between dog and cat owners (36, 41, 42) (Table 5).

**Table 5:** Overview of research themes where associations with the LAPS were studied (*n* = 175).

**Research themes**	**Count**
**Human health and conditions**	
Human mental health	47
Social support	34
Human physical health	25
Human loneliness	11
Human lifestyle	8
**Owner behavior and attitudes targeting their pet**	
Owner behavior toward pet	50
Attitudes toward, and use of, veterinary services	13
Willingness to pay for pet products and services	1
**Other research themes**	
Human socio-demographic characteristics^a^	28
Human personality	25
Animal species^b^	8
Scale construction/psychometric assessment	12
Other	13

a Socio-demographic predictors include country differences, age, gender, and social status indicators (e.g. income and education), and living alone.

b Comparison of LAPS scores across species.

### Study 1: conclusion

The English language studies that have used the LAPS were predominantly from North America and Europe, and the majority had respondents with either a dog, a cat or both. Therefore, it is important to ensure that the Brief-LAPS scale is psychometrically sound for studies of attachment to dogs and cats. Inclusion of other species appears to be less important as they are seldomly studied, although it would still be desirable if the Brief-LAPS could be used in such cases.

The preferred scale construction method was to use composite scores (range from 0 to 69), and the composite score was often used in group mean comparisons and in analyses of correlations.

The LAPS was used in many research fields, including to study associations between animal attachment and human health and behavior, human socio-demographic characteristics and human personality, as well as treatment decision-making in veterinary care. Other studies have explored levels of human attachment across different animal species.

## Study 2: Content analysis of LAPS items

### Study 2: introduction

Since we are not developing a new scale but adapting an existing one, many of the steps involved in the development and validation of a scale are irrelevant here (43). It is important, however, to maintain the content of the original construct. We therefore aimed to include items from each of the 3 sub-dimensions of the LAPS. To guide us in the choice of items to retain from the 3 sub-dimensions, the idea of content validity is central. We use content validity as a term following, for instance, Boateng et al. (43) and Furr (44). The idea of content validity is to evaluate whether there is a match between what researchers are claiming that a scale measures and the actual scale content. As Furr puts it, “…if scale scores are to be interpreted in terms of a particular psychological phenomenon, then its content must reflect important facets of that phenomenon—no more, no less” (44).

It is recommended in the scale development literature that content validity should be evaluated by experts in the field with a solid understanding of the relevant psychological phenomenon to avoid bias, preconceptions, and flawed opinions from the scale creators (43, 44). In our effort to include the most accurate items from each of the sub-dimensions of the full LAPS in the Brief-LAPS version, we therefore invited experts in human-animal attachment to evaluate the content validity of the 23 LAPS items.

### Study 2: methods

Study 2 was approved by the Institutional Review Board at the Faculties of Science and of Health and Medical Sciences at the University of Copenhagen (journal no.: 504/0465/24-5000).

We sought experts from the International Society for Anthrozoology (ISAZ), as a community of scholars and scientists working in the relevant study field (https://isaz.net/). ISAZ agreed to distribute an e-mail on May 7, 2024, via its voluntary membership listserv inviting a total of 237 members to participate in the survey.

Fifty-four of the 237 invitees completed the survey (response rate~23%). Table 6 presents an overview of the participant characteristics. Most respondents were female. There was considerable variation with respect to age (26% < 40 years; 48% 40–59 years; 26% >59 years). Most participants had a master's degree or a PhD or equivalent doctoral degree. The respondents also came from diverse occupational backgrounds, but most worked in academia or animal-assisted interventions. Participants were asked to self-rate their knowledge about HAI and owners' attachments to their pets on a scale from 1 (very low) to 7 (very high). The majority considered their knowledge to be high (self-reported knowledge about HAI: M = 5.7; and about owners' attachments to their pets: M = 5.1). Eight of the participants did not know the LAPS scale at all, 24 had heard about the scale, but knew little about it. Thirteen knew the scale quite well, and nine stated that they knew the scale very well.

**Table 6:** Socio-demographic and self-reported knowledge levels of the expert sample (*n* = 53–54).

**Expert characteristics**	***n***
**Gender**	
Female	47
Male	6
Other/do not wish to say	1
**Age**	
18–39 years	14
40–59 years	26
>59 years	14
**Highest completed level of education**	
Higher education (but not bachelor's/master's degree)	1
Undergraduate or bachelor's degree at college/university	4
Master's degree at college/university	12
PhD or equivalent doctoral degree	37
**Occupational field**	
Veterinary (e.g. as veterinarian, veterinary behaviorist, and veterinary technician/nurse)	6
Animal behavior (e.g. non-veterinary behaviorist, trainer)	12
Human medicine (e.g., medical doctor/clinician, nurse/nurse practitioner, clinical psychologist)	3
Social work	5
Academia (e.g. as researcher/lecturer at the university, professional school, academy, college)	38
Animal-assisted interventions	21
Other	8
**How familiar are you with the LAPS scale?**	
I don't know the scale at all	8
I have heard about the scale, but have limited knowledge about it	24
I know the scale quite well	13
I know the scale very well	9
	**Mean (s.d)**
Self-reported expert knowledge about human-animal interaction (*n* = 53)^a^	5.7 (1.3)
Self-reported expert knowledge about owner's attachment to their pet (*n* = 53)^a^	5.1 (1.3)

a Response options ranged on a scale from from 1 (very low) to 7 (very high).

Participants were asked to consider each of the 23 LAPS question items (which are formulated as statements), in the context of six response options. The first response gave participants the opportunity to flag statements that they found to be unsuitable measures of attachment (“Unsuitable statement to describe pet owners' attachment”). Three response options covered the LAPS sub-dimensions (“People Substitution”, “Animal Rights/Welfare”, “General Attachment”). One response option was available for statements where the LAPS sub-dimension was unclear or included several of them (“Unclear or several sub-dimension(s)”). Finally, an option was available if the statement was considered to belong to another dimension of attachment (“A different attachment dimension”) (instruction text details are appended in Supplementary File 2).

### Study 2: analysis

The frequency of respondents' evaluations of all 23 items is reported. We also examined whether the evaluation of five statements that many experts rated as “unsuitable” or belonging to a different attachment dimension differed across expert characteristics. We further examined whether the general assignment of the 23 statements to all six response options differed across expert characteristics. In both examinations, chi^2^ tests were conducted, and a *p* < 0.05 was considered statistically significant.

### Study 2: results

**Figure 2:**
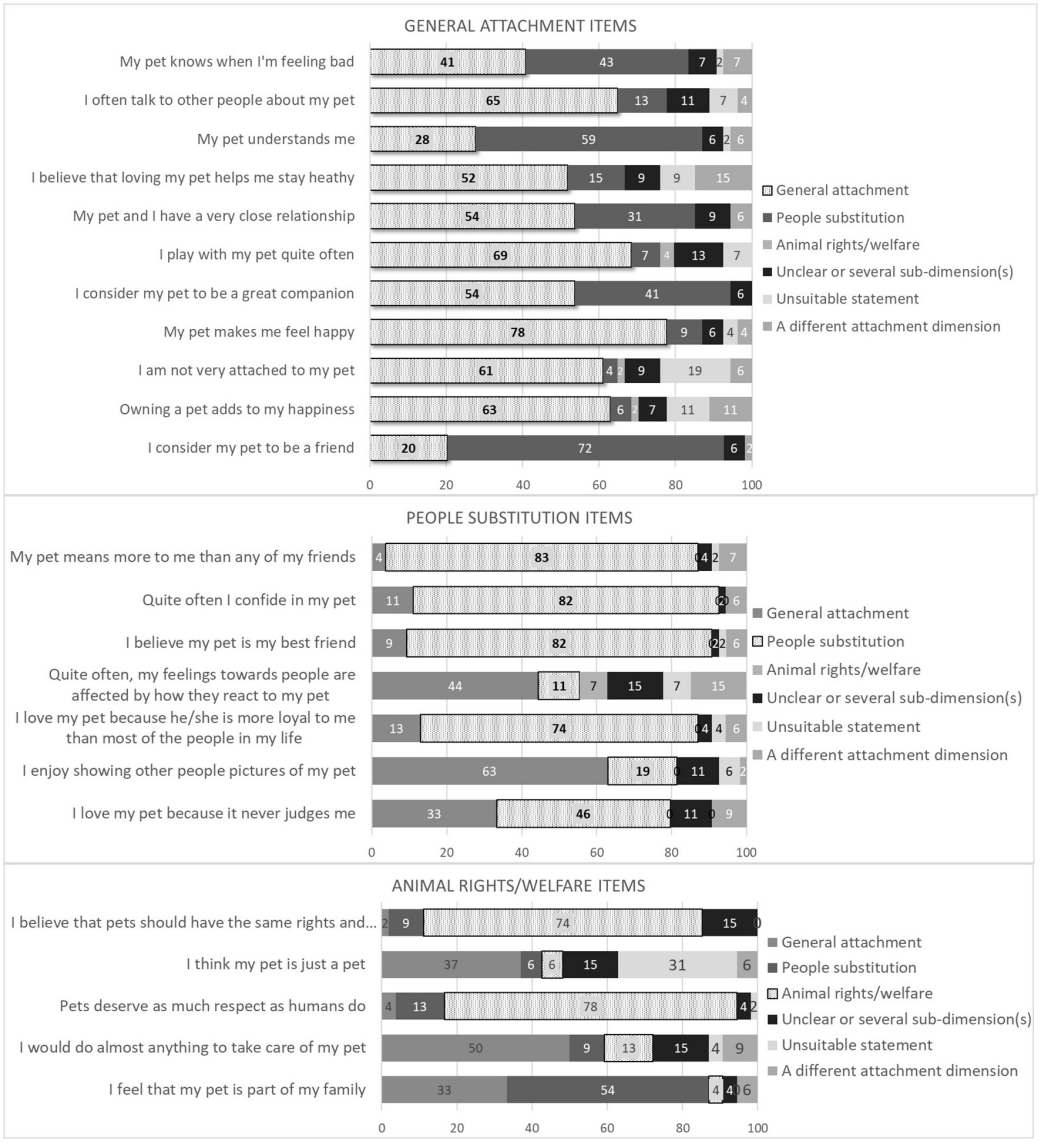
How experts in human-animal interaction (*n* = 54) evaluated the item-specific content of the 23 LAPS items (presented as 100% stacked bar charts). Items are placed in the sub-dimension it was assigned to by Johnson et al. (3).

The experts' evaluations of the 23 LAPS items are presented in Figure 2. The figure consists of 3 graphs, one for each sub-dimension of the LAPS. Each item was placed in the sub-dimension graph that it was assigned to by Johnson et al. (3).

There were five items where a relatively large share (> 20%) of the experts indicated that the statement was unsuitable to assess attachment or did not belong to one of the 3 LAPS sub-dimensions (i.e. that it belonged to a different attachment dimension). The items were: “Owning a pet adds to my happiness”, “I am not very attached to my pet”, “I think that my pet is just a pet”, “I believe that loving my pet helps me stay healthy” and “Quite often, my feelings toward people are affected by how they react to my pet”. We identified no differences in the evaluation of the five statements as unsuitable or belonging to a different attachment statement across age, educational levels, occupational characteristics (comparing people working in academia and animal-assisted interventions with other occupations), self-reported expertise in HAI and owner's attachment to pets, and knowledge about the LAPS.

Figure 2 also shows that many items were not unequivocally assigned to the correct sub-dimension by the experts. Just 13 items (6 from General Attachment, 5 from People Substitution, and 2 from Animal Rights/Welfare) were assigned to the same sub-dimension by more than 60% of experts. Of these, only 11 items (5 from General Attachment, 4 from People Substitution, and 2 from Animal Rights/Welfare) were assigned to the original sub-dimension laid out in Johnson et al. (3) by more than 60% of experts. Five of the 11 items belonging to the General Attachment sub-dimension were considered indicators of People Substitution by more than 30% of experts. For example, the General Attachment statement “My pet knows when I am feeling bad” was considered to belong to General Attachment by 40.7% and to People Substitution by 42.6% of experts. Conversely, 3 of the 7 items belonging to People Substitution were considered to be indicators of General Attachment by more than 30% of experts. For instance, “I love my pet because it never judges me” was considered an indicator of General Attachment by 33.3%, and of People Substitution by 46.3% of experts.

We examined whether the assignment of the 23 statements to all 6 response options differed across expert characteristics and found almost no differences. Only 1 statistically significant difference (at the 0.05 level) was identified. Specifically, experts from academia were more likely to evaluate the item “I think my pet is just a pet” as an unsuitable statement to describe pet owners' attachment, while experts not working in academia were more likely to evaluate the item as belonging to the General Attachment dimension.

### Study 2: conclusion

More than 20% of the participants considered 5 of the 23 LAPS items to be unsuitable indicators of owner's attachment to pets or to belong to other domains of attachment that are not intended to be covered by the LAPS. Also, experts did not unequivocally assign many of the 23 items to the same sub-dimension.

Experts agreed that just 2 (of the original 5) items were good indicators of Animal Rights/Welfare. The remaining 3 items were seldomly assigned to this sub-dimension. For many of the remaining items, experts had difficulties separating whether they were indicators of General Attachment or People Substitution. In some cases, items that according to the original study (3) are supposed to be indicators of General Attachment were evaluated as indicators of People Substitution by most of the experts, and vice versa. It seems reasonable to conclude that many of the General Attachment items and People Substitution items reflect both dimensions.

A limitation of this expert data is that only 54 of the 237 invitees responded to the questionnaire. This could introduce nonresponse bias where those who did not respond differ from those who did in a way that is relevant to the study findings. However, apart from one statistically significant difference between experts from academia and other experts, no differences in the evaluation of items were identified across respondent characteristics such as age, gender, occupation, education, and self-reported knowledge. Assuming that we were able to compare relevant expert characteristics, this suggests that nonresponse bias is limited.

## Study 3: Development of the Brief-LAPS scale

### Study 3: introduction

To develop the Brief LAPS scale we utilized findings from Study 2 to identify the items that HAI experts evaluated as having the strongest content validity.

Additionally, Study 1 delivered insights that are consequential for how we develop the Brief-LAPS. Firstly, in practical research, composite scores are predominantly used to calculate the LAPS variable. Secondly, many studies (70 of 175) use these composite scores to conduct group comparisons. When composite scoring is used, the psychometric literature recommends that a scale needs to possess what is referred to as scalar invariance (45, 46) (For technically interested readers, a description of scalar invariance and other types of measurement invariance levels is provided in Supplementary File 4).

If a scale does not have this level of measurement invariance, comparison of group differences may be biased. Examples from HAI research where a lack of scalar invariance could lead to biased results include differences in attachment to pets between cat and dog owners, and between individuals who live alone and individuals who live with others.

For this reason, our aim was to develop a brief scale that achieved scalar invariance.

### Study 3: methods

Study 3 was approved by the Institutional Review Board at the Faculties of Science and of Health and Medical Sciences at the University of Copenhagen (journal no.: 504/010300/22-5000).

Pet owners in three European countries were surveyed: Austria, Denmark, and the UK. A sample of participants were recruited from pre-existing panels of citizens. The sampling scheme was intended to be representative of the overall populations in all three countries, i.e. both participants with and without pets were included. The study data is fairly representative for the target populations with regard to age, gender and living areas in the 3 countries. However, in this paper we only utilize data from respondents that self-identified as pet owners. Since the samples were drawn from pre-existing panels, it should be noted that actual random sampling was not carried out. Details about the study data, including panel recruitment criteria and average error rates have been described in detail elsewhere (47). The questionnaire included several themes (see the full questionnaire in Supplementary File 3). In this paper we included 884 pet owners from Austria, 688 from Denmark, and 767 from the UK.

The 23 LAPS statements and response options in English were replicated from the original study (3), and we used existing translated versions of the LAPS in German (19) and Danish (48). Table 7 presents the number of species that were declared as the favorite pet for each country and in total.

**Table 7:** Species of the respondent's favorite pet - per country and in total.

**Species**	**Country**			
	**Austria**	**Denmark**	**UK**	**Total**
Dog	332	359	393	1,084
Cat	443	250	267	960
Horse	14	17	13	44
Rabbit	22	16	16	54
Rodent (e.g. hamster, guinea pig, chinchilla, mouse/rat)	21	17	14	52
Bird	14	14	14	42
Reptile (e.g. lizard, snake, turtle)	15	4	9	28
Fish (both aquarium fish and fish in garden pond)	17	6	36	59
Other than the abovementioned	6	5	5	16

Dogs and cats were clearly the favorite pets; there were very few cases where horses, birds, reptiles, fish, rodents, rabbits, or other animal species were selected. We therefore decided to exclude the latter species in the development of the Brief-LAPS and only focus on respondents that mentioned dogs and cats as their favorite pet (*n* = 2,037).

### Study 3: analysis

Item exclusion strategy: The exact number of items to include in the Brief-LAPS was not decided in advance, but we hoped that we could reduce the scale to include a maximum of 8 items, and preferably 6–8 items. The item removal process involved the use of confirmatory factor analysis (CFA) (49). We examined modification indices which provide information about the items in a CFA that contribute to a poor model fit (50, 51). Items that contributed the most to a poor model fit were incrementally removed until we reached 8 remaining items. At that point we started evaluating several candidate Brief-LAPS scales (consisting of different combinations of items) in an iterative procedure where measurement invariance (see next sub-section) was also evaluated.

#### Measurement invariance approach and cut-off points

The Brief-LAPS was developed using the method of measurement invariance. As clarified in the introduction to Study 3, we aimed to obtain measurement invariance at the scalar level.

After the identification of a brief scale, assessments of measurement invariance were carried out using multi-group confirmatory factor analysis (MG-CFA) (52). Chiefly, there are 3 main invariance levels that step by step establish higher levels of invariance: configural invariance, metric invariance, and scalar invariance (46, 53).

Our aim was to reach scalar invariance. As noted earlier, at this invariance level it is justifiable to make group mean comparisons using composite scores (i.e. all observed item scores are summed together). If the criteria for metric and scalar invariance were not fulfilled, we released restrictions with a view to identifying partial metric or partial scalar invariance (54). (A technical description of these invariance levels is appended in Supplementary File 4).

In CFA, multiple estimations techniques are available where the maximum likelihood (ML) estimation is the default. However, ML estimation is not recommended where input variables are non-normally distributed (55). There are just four response options available for all LAPS items, and the distribution of many of the items is highly skewed. So, ML estimation is not appropriate. Instead, we employed categorical ordered MG-CFA where the weighted least squares mean and variance adjusted (WLSMV) estimation is used based on a polychoric correlation matrix [see e.g. Pendergast and colleagues (56)]. Mplus version 8.6 (64-bit) (57) was used for this analysis.

There are different guidelines available for assessing acceptable model fit in categorical ordered MG-CFA (58). We followed the recommendations of Rutkowski and Svetina (52) for one-factor models where cut-off points for the root mean squared error approximation (RMSEA) and the comparative fit index (CFI) are set out. More specifically, in our evaluation of the overall fit of the configural, metric, and scalar models, the RMSEA could not exceed 0.055 [here, there are no recommendations for the CFI (52)]. In our incremental tests we examined the following changes in RMSEA and CFI: when comparing the metric model with the configural model, the cut-off value for ΔRMSEA was +0.05, and for ΔCFI – 0.004. When comparing the scalar model with the metric model, the cut-off value for ΔRMSEA was +0.01, and ΔCFI for −0.004 (52). Before evaluating higher levels of invariance, we ensured that the requirements for invariance at lower levels were fully met. Additionally, we report model results from chi^2^ testing. However, it should be noted that chi^2^ testing is sensitive to sample sizes in factor analysis and tends to reject acceptable models when there are large sample sizes (59). Therefore, we mainly rely on the RMSEA and CFI test results. All analyses of measurement invariance were conducted in *Mplus Version 8.6 (64-bit)* (57).

The sub-groups that were checked for invariance were selected based on Study 1 identifying variables that were often examined for associations with the LAPS in English peer-reviewed research. The following sub-groups were compared in the MG-CFA examination of measurement invariance: country (Austria, Denmark, UK), favorite animal species (dog or cat), gender (female or male), age (old or young), household income (high income or not high income), living alone (yes or no), children in the family (yes or no), perceived loneliness (high or low), loneliness (high or low), tangible support (high or low), social support (high or low) (details about the variables and coding are presented in Supplementary File 4).

### Study 3: results

The development of the Brief-LAPS scale involved several steps and trials. First, we removed the 5 items that many experts evaluated as unsuitable. Among the remaining 18 items, we then attempted to include only the items that were consistently assigned to the same sub-dimension by the experts. We set the threshold for a consistent assignment at >60%, resulting in 11 consistently assigned items. We used modification indices to remove 4 items after which model fit for the remaining 7 items was acceptable. However, the pool of 7 items mainly included People Substitution items (5 of 7 items), and just 1 item from General Attachment and 1 from Animal Rights/Welfare. Further, the item from the General Attachment scale that was retained was: “I consider my pet to be a friend”. This was predominantly considered a People Substitution indicator by the experts. We concluded that this content representation of the original LAPS scale was too unbalanced, and we therefore rejected it as a suitable brief scale.

We then expanded the model search with items that were not unequivocally assigned to 1 sub-dimension by the experts, thus giving 18 items as input variables to the CFA. An item removal strategy was initiated where modification indices helped flag items contributing the most to a poor model fit. Using this removal strategy, we identified several 7-item scales that exhibited acceptable model fit. However, for most of these models the subsequent assessment of measurement invariance failed to meet the cut-off points multiple times. One model, however, did meet the cut-offs for measurement invariance across almost all of the compared subgroups. Model fit for this scale was acceptable [Chi^2^ = 69.69 (15); RMSEA=0.044; CFI=0.996; *n* = 2,037] and Cronbach's α was satisfactory (0.86 (95% CI: 0.85–0.87). Further, the item pool was quite balanced, as it included items from all 3 sub-dimensions without any single sub-dimension dominating the pool.

The resulting Brief-LAPS is presented in Table 8. It consists of 7 items where all factor loadings are >0.600 and commonalities are in the range 0.400–0.805. The sub-dimension that the particular item was assigned to by Johnson et al. (3) is presented in a separate column.

**Table 8:** An overview of the seven items included in the Brief-LAPS and factor loadings from CFA.

**Question statement**	**Standardized factor loadings**	**Commonalities**	**Original LAPS sub-dimension^A^**
My pet knows when I'm feeling bad	0.707	0.527	GA
My pet and I have a very close relationship	0.899	0.805	GA
I play with my pet quite often	0.721	0.518	GA
I consider my pet to be a friend	0.841	0.706	GA
My pet means more to me than any of my friends	0.634	0.400	PS
I love my pet because it never judges me	0.701	0.489	PS
Pets deserve as much respect as humans do	0.755	0.568	AR

CFA test results (extracted from Mplus output): Chi^2^ = 69.69 (14); RMSEA = 0044; CFI = 0.996; n = 2,037; Cronbach's α = 0.86 (95% CI: 0.85–0.87).

^A^GA, General Attachment; PS, People Substitution; AR, Animal Rights/Welfare.

### Measurement invariance results

The evaluation of measurement invariance is laid out in Supplementary File 4 where a table with model fit for all invariance levels along with overall RMSEA and CFI, as well as ΔRMSEA and ΔCFI values is presented. We detected invariance at scalar level for the following variables: animal species, gender, age, income, household composition, perceived loneliness, UCLA loneliness scale, tangible and emotional support. Thus, composite scores can be used for such variables with limited risk of biased estimations. However, in the assessment of measurement invariance for countries, we did not detect scalar invariance. Therefore it was explored if it was possible to reach partial scalar invariance for country comparisons. Indeed, we identified partial scalar invariance for countries by freeing 8 (of 21) threshold parameters. See details in the notes to the table in Supplementary File 4.

We consider the implications of the lack of full scalar invariance between countries in the Discussion. However, when we conducted CFA and calculated Cronbach's alpha internal consistency coefficients for each country separately, model fit was acceptable [Austria: Chi^2^ = 34.323 (14); RMSEA = 0.043; CFI = 0.996; *n* = 773]; Cronbach's α=0.86 (95% CI: 0.84–0.88). Denmark: Chi^2^ = 38.834 (14); RMSEA = 0.054; CFI = 0.995; *n* = 607; Cronbach's α = 0.84 (95% CI: 0.82-0.86) UK: Chi^2^= 34.328 (14); RMSEA=0.047; CFI = 0.996; *n* = 657; Cronbach's α=0.86 [95% CI: 0.84–0.88)].

### Study 3: conclusion

A 7-item Brief-LAPS scale was developed in which all 3 of the LAPS sub-dimensions are represented. Still, it should be noted that the item coverage of the three sub-dimensions is a little unbalanced. There is only 1 Animal Rights/Welfare item, as this was the only item that ensured acceptable model fit of just 2 possible items considered suitable by the experts in Study 2. There is also a slight overrepresentation of General Attachment items (cf. Table 8), but we note that one of these items (“I consider my pet to be a friend”) is mainly considered by the experts to be an indicator of People Substitution. Furthermore, one of the General Attachment items that is included (“My pet knows when I'm feeling bad”) is considered to be a General Attachment indicator by some of the experts and a People Substitution indicator by other experts. Thus, the 3 attachment domains from the full LAPS are covered in the Brief-LAPS.

## Study 4: Comparison of the full LAPS and Brief-LAPS

### Study 4: introduction

To ensure that results obtained using the Brief-LAPS are compatible with those obtained using the full LAPS, we compared their associations with a number of variables, including socio-demographic factors, attitudes to veterinary services targeting pets, loneliness, and social support.

### Study 4: methods

The same data employed in Study 3 were used in this analysis (see previous details). The following variables were developed for the analysis below:

Gender: (0 = male; 1 = female). Two other gender response options were available (“Other” and “I do not wish to tell”), but these were treated as missing data, since they were very rarely selected.

Age: Number of years (range 18–86).

Income: Respondents were asked about the household's annual income in the relevant currency of each country. For each country, we divided annual income into five equally sized groups. Where “I don't know” was selected, this was treated as missing data.

Household composition: whether the respondent lives alone, with 2 or more adults, or in a household where there are children.

Attitudes to advanced veterinary services targeting pets: here we used 3 items from a recent paper where the same data was used as in this study (47). The items are: “My pet should have access to the same diagnostic tests that are available to human patients”, “My pet should have access to the same treatment options that are available to human patients” and “The advanced care available in modern veterinary medicine is unnecessary—animals should not be treated in the same way as humans.” Response options ranged from 1: strongly disagree to 7: strongly agree.

Emotional and tangible support: This is a 6-item measure of social support that covers two dimensions, namely emotional and tangible support (60). We calculated composite scores for both dimensions.

UCLA 3-item loneliness scale: This 3-item scale measures loneliness (61).

Perceived loneliness: this single-item measure is used in national surveys in the UK (Community Life Survey, England 2017 to 2018: Statistical bulletin) and the Nordic/Baltic countries (62) as a brief measure of perceived loneliness.

### Study 4: analysis

For each of the abovementioned measures we report the strength and direction of the association with the full LAPS and with the Brief-LAPS. Depending on the type of variable used in the analysis, we used either Spearman's correlation coefficients, Pearson's correlation coefficients, or analyses of variance for categorical variables with more than 2 values.

The full LAPS and the Brief-LAPS have a different score range (Full LAPS = 0–69; Brief-LAPS = 0–21). Therefore, when reporting group differences in analyses of variance, we rescaled both LAPS measures into the same metric, so that they range from 0 (lowest attachment) to 1 (highest attachment).

### Study 4: results

The full LAPS and the Brief-LAPS are very highly correlated (Pearson's r = 0.95).

The strength and direction of associations between sociodemographic variables and the full and Brief-LAPS are presented in Table 9 (for gender, age, income). The patterns are very similar.

**Table 9:** Association between sociodemographic variables (gender, age, income) and the Full and Brief-LAPS—reported for each country.

**Socio-demographic characteristics**	**Austria**		**Denmark**		**UK**	
	**Full LAPS**	**Brief-LAPS**	**Full LAPS**	**Brief-LAPS**	**Full LAPS**	**Brief-LAPS**
Gender^a, b^	0.25^***^	0.22^***^	0.21^***^	0.19^***^	0.24^***^	0.18^***^
Age^c^	0.04	0.06	−0.08	−0.06	0.04	0.06
Income^c^	−0.20^***^	−0.17^***^	−0.24^***^	−0.22^***^	−0.13^**^	−0.12^**^

^**^p < 0.01; ^***^p < 0.001.

^a^Gender variable: (0 = male; 1 = female). So, a positive correlation indicates that females score higher on the Full LAPS and Brief-LAPS.

^b^Spearman's rank-order correlation coefficients and tests of association.

^c^Pearson's correlation coefficients and tests of association.

In Table 10, the scores on the Full and Brief-LAPS are presented across different household compositions (living alone, 2 or more adults, and households with children). In all countries, the score patterns move in similar directions for both scales across the 3 household types. Results from F-tests are also similar in that all *p*-values are significant.

**Table 10:** The associatio between household type and the Full and Brief-LAPS—reported for each country.

**Household type**	**Austria**		**Denmark**		**UK**	
	**Full LAPS**	**Brief-LAPS**	**Full LAPS**	**Brief-LAPS**	**Full LAPS**	**Brief-LAPS**
Lives alone	0.73	0.74	0.71	0.74	0.72	0.74
More than one adult (no children)	0.74	0.76	0.63	0.66	0.75	0.76
Children in household	0.67	0.69	0.60	0.64	0.70	0.71
*p*-value (F-test)	<0.001	<0.001	<0.001	<0.001	<0.01	<0.05

The Full LAPS and Brief-LAPS were rescaled to range from 0 to 1 in this table to facilitate comparison.

Table 11 presents the strengths and directions of association between attitudes to advanced veterinary services targeting pets and the full and Brief-LAPS. The patterns appear very similar. In the final comparisons (see Table 12), we also find that the patterns between tangible support, emotional support, and loneliness are very similar for the Full and Brief-LAPS.

**Table 11:** Associations^a^ between attitudes to advanced veterinary services targeting pets and the Full and Brief-LAPS—reported for each country.

**Attitude statements**	**Austria**		**Denmark**		**UK**	
	**Full LAPS**	**Brief-LAPS**	**Full LAPS**	**Brief-LAPS**	**Full LAPS**	**Brief-LAPS**
“My pet should have access to the same diagnostic tests that are available to human patients”	0.59^***^	0.57^***^	0.57^***^	0.54^***^	0.50^***^	0.48^***^
“My pet should have access to the same treatment options that are available to human patients”	0.58^***^	0.54^***^	0.55^***^	0.51^***^	0.48^***^	0.47^***^
“The advanced care available in modern veterinary medicine is unnecessary—animals should not be treated in the same way as humans.”	−0.27^***^	−0.23^***^	−0.33^***^	−0.30^***^	−0.19^***^	−0.14^***^

^***^p < 0.001.

^a^ Pearson's correlation coefficients.

**Table 12:** Associations^a^ between social support (divided into practical and emotional support) and loneliness (divided into perceived loneliness and the UCLA loneliness scale) and the Full and Brief-LAPS—reported for each country.

**Support/loneliness indicator**	**Austria**		**Denmark**		**UK**	
	**Full LAPS**	**Brief-LAPS**	**Full LAPS**	**Brief-LAPS**	**Full LAPS**	**Brief-LAPS**
Tangible support	−0.01	−0.00	−0.12^*^	−0.14^*^	−0.13^***^	−0.14^***^
Emotional support	0.06	0.07^*^	−0.10^*^	−0.12^*^	−0.19^***^	−0.20^***^
UCLA loneliness scale	0.16^***^	0.14^***^	0.24^***^	0.23^***^	0.11^**^	0.09^**^
Perceived loneliness	0.08^*^	0.05	0.25^***^	0.23^***^	0.07	0.05

^*^p < 0.05; ^**^p < 0.01; ^***^p < 0.001.

^a^Pearson's correlation coefficients.

### Study 4: conclusion

The Brief-LAPS produces associations with other variables that are very similar to those identified with the full LAPS. The variables that were used to compare the full and Brief-LAPS covered a great deal of the content of variables that are typically correlated with the LAPS in applied research (cf. Table 5).

## Discussion

Our aim was to develop a brief version of the full LAPS Scale. After removal of 16 of the original 23 items we created a 7-item Brief-LAPS scale. The items were chosen based on evaluations from HAI experts, and we ensured that the Brief-LAPS included items from all 3 sub-dimensions of the full LAPS (3). We further demonstrated that the Brief-LAPS is highly correlated with the full LAPS (Pearson's r = 0.95), and that it produces associations with other variables that are very similar to those typically correlated with the full LAPS. Further, a strict strategy was used to evaluate the psychometric properties of the Brief-LAPS, which required meeting the criterion of scalar invariance. On most counts, the Brief-LAPS fulfilled this requirement.

Overall, we believe that the Brief-LAPS is a relevant option for scholars who aim to minimize questionnaire length. Still, it is important to highlight several limitations of the present study, and provide recommendations for future research.

It was a limitation that we failed to obtain scalar invariance for the three countries studied. This lack of scalar invariance weakens the applicability of the Brief-LAPS in cross-country comparisons. Specifically, using composite scores to compare mean differences on the Brief-LAPS between countries increases the risk of drawing false conclusions. A false conclusion might arise for example if the mean of the Brief-LAPS is found to be different between two countries (e.g., at the 0.05 level of significance) as a result of lack of measurement invariance, rather than a real difference existing between the two countries.

In a follow-up examination, partial scalar invariance was identified for the three countries. This opens the opportunity for researchers who aim to use the Brief-LAPS for cross-country comparisons to do so using MG-CFA, provided that a partial scalar model can be identified in their data. However, it is debated whether mean-level tests for partially invariant models are accurate (54). Either way, Table 4 shows that most researchers use composite scoring. Therefore, the aforementioned MG-CFA approach may not be a popular choice in practice. Researchers who aim to use composite scoring for cross-country comparisons should be extra aware of the level of difference between the countries being compared. If there is a strong difference in mean Brief-LAPS scores it is likely to be real, whereas if the difference is modest or minimal the risk of a false conclusion increases. In such cases it is relevant to accompany the group mean scale comparison with an item-by-item group comparison.

A further limitation related to the psychometric properties of the Brief-LAPS is that it was developed using samples of pet owners in three European countries. This means that it is uncertain that the scale will possess the same acceptable psychometric properties in other countries. Therefore, psychometric testing of the Brief-LAPS is recommended in future studies where the scale is featured in other study populations and languages. This recommendation is reinforced by the aforementioned limitation that we failed to achieve scalar invariance across the three countries examined. It will be particularly important to conduct psychometric assessment in countries where the original factorial structure of the LAPS was not replicated (16, 17, 20). Possible reasons for this type of cross-cultural invariance include lack of systematic translation and adaptation across languages-specific questionnaire versions (16, 63)—cultural differences across countries and languages (16)—and differences in social desirability bias and satisficing (64). These sources of potential invariance could be explored in future studies.

A further limitation is that there is a moderate degree of sub-dimension imbalance. Specifically, compared with the full LAPS, the share of General Attachment and People Substitution items is higher than the share of Animal Rights/Welfare items. For instance, in the full LAPS, 22% of the items are Animal Rights/Welfare items, while the share is 14% in the Brief-LAPS. The underrepresentation of Animal Rights/Welfare items could be an attention point for researchers with a focus on ethical orientations and behaviors, and for researchers where domain-level interpretation is important. Researchers with one or both of these ambitions could consider using the full LAPS, even though Study 2 in this paper suggests that the content validity of these sub-dimensions is suboptimal. Thus, the experts did not unequivocally assign the 23 items to the original sub-dimension suggested by Johnson et al. (3).

We also wish to mention that the response rate of the surveyed experts in Study 2 was relatively low (23%). This could introduce nonresponse error such that the results of the experts' evaluations of the 23 items are biased in an indeterminate direction. The evaluation of items was similar across several respondent characteristics, however, suggesting that nonresponse bias is limited. Still, we cannot rule out bias, and future studies could explore content validity of the 23 items further in a sample with higher response rate.

Finally, the assessment of measurement invariance was only carried out using data from owners who had stated that a cat or dog was their favorite pet. However, when we considered owners reporting that their favorite pet was something other than a cat or dog, the model fit of the Brief-LAPS was acceptable [Chi^2^ = 35.64 (15)]; RMSEA = 0.073; CFI = 0.994; Cronbach's alpha=0.897 (95% CI: 0.878–0.915; *n* = 293), suggesting that the Brief-LAPS is able to identify attachment quite well for other species too.

## Conclusion

This study developed the Brief-LAPS and found it to be a psychometrically sound brief version of the full LAPS. Since the full LAPS and the Brief-LAPS are relatively akin content-wise and produce similar results in associational analyses, we conclude that the Brief-LAPS can be substituted for the full LAPS in cases where brevity is needed. Specifically, we recommend using the Brief-LAPS in research where survey fatigue or costs are potential issues.

The Brief-LAPS is a less viable option when researchers consider it important to obtain as much variance on the latent scale as possible, as the full LAPS with its 23 items produces a larger score interval (0–69). The Brief-LAPS should not be used in research where the intention is to report mean scores on the full LAPS, e.g. with a view to comparing scores reported in other studies where the full scale was used. It should also not be used when researchers aim to use one or more of the 3 LAPS sub-dimensions in their analyses. Finally, caution should be exercised when researchers compare Brief-LAPS scores between countries.

Scoring instructions for the Brief-LAPS that give a composite score (range: 0–21) are presented in Appendix A.

## Data Availability

The raw data supporting the conclusions of this article will be made available by the authors, without undue reservation.

## Appendix A

**The seven items used in the Brief-LAPS**

Item 1. My pet knows when I am feeling bad

Item 2. My pet and I have a very close relationship

Item 3. I play with my pet quite often

Item 4. I consider my pet to be a friend

Item 5. My pet means more to me than any of my friends

Item 6. I love my pet because it never judges me

Item 7. Pets deserve as much respect as humans do

**Response options (numeric values)**

Strongly disagree (0)

Somewhat disagree (1)

Somewhat agree (2)

Strongly agree (3)

**Computation of the Brief-LAPS (composite score method)**

Brief-LAPS = Item 1 + Item 2 + Item 3 + Item 4 + Item 5 + Item

6 + Item 7.
