# Supplementary material for: The development of a brief version of the Lexington Attachment to Pets Scale (Brief-LAPS)
Source: Front Vet Sci. 2025 Sep 2;12:1619187. doi: 10.3389/fvets.2025.1619187 (PMC12439530; doi:10.3389/fvets.2025.1619187)
Supplement: Supplementary file 1 [file Supplementary_file_1.docx]

**Supplementary file 1**

**LAPS literature review**

The search term “Lexington Attachment to Pets Scale” was used for the literature review. Initially, LAPS was also tried out as search term, which is a frequently used abbreviation. However, this term was abandoned, as this led to a very large number of hits, because LAPS is an abbreviation and concept that is used in other research fields (e.g., “Local Analysis and Prediction System”). Since all manuscripts included in the review mention the full name at least once before using the abbreviation, we believe that we missed very few, if any, papers.

In total, 175 papers relevant to this study were identified through the four electronic databases SCOPUS, BASE, Web of Science, and Google Scholar.

First, the search was carried out in SCOPUS (total hits before inclusion criteria were considered=74) followed by BASE (total hits before inclusion criteria were considered =10), Web of Science (total hits before inclusion criteria were considered =53), and Google Scholar (total hits before inclusion criteria were considered =853). The significantly higher number of identified papers from Google Scholar can be attributed to its full-text analysis capabilities.

After removal of papers that did not meet the inclusion criteria, 61 papers were added from the SCOPUS search. After removal of papers that did not meet the inclusion criteria and were duplicates with the previously used electronic database(s), 0 papers were added from the BASE search, 2 additional papers were added from the Web of Science search, and 112 additional papers were added from the Google Scholar search.
