# Supplementary material for: The development of a brief version of the Lexington Attachment to Pets Scale (Brief-LAPS)
Source: Front Vet Sci. 2025 Sep 2;12:1619187. doi: 10.3389/fvets.2025.1619187 (PMC12439530; doi:10.3389/fvets.2025.1619187)
Supplement: Supplementary file 2 [file Supplementary_file_2.docx]

**Supplementary file 2**

**Instruction text provided to the experts in human-animal interaction:**

“INSTRUCTION:

The statements that will appear on the following pages are used to measure pet owners’ attachment to their pets. Please consider all kinds of animal species and all kinds of owners when you assess each statement. For each statement, you will have six response options available. On the next page, an overview and a detailed explanation of the response options are provided.”

“Click Next to go to next page. “

“INSTRUCTION CONTINUED:

For each statement, please first consider whether you think it serves to describe pet owners’ attachment to pets – both their personal attachment and their views about the moral rights of the pets.

If you do not think that the statement reflects such attachment, please click on the response:

Unsuitable statement to describe pet owners’ attachment.

If you do think that the statement reflects how some or all owners are attached to their pets, then please click on the sub-dimension of owner attachment it belongs to. The three sub-dimensions we want you to consider are:

People substitution, i.e., owners engage with their pet in the same way that they would with humans.

Animal rights/welfare, i.e., owners think that pets should have legal/moral rights and privileges and are concerned with the welfare of pets.

General attachment, i.e., any other kind of owner-pet attachment that does not fall under the two former dimensions.

If you think it is unclear which sub-dimension a statement belongs to, or that a statement cannot exclusively be said to belong to just one of the sub-dimensions click on:

Unclear or several sub-dimension(s).

If you think that the statement belongs to an entirely different attachment dimension not defined by the three sub-dimensions above, please click on:

A different attachment dimension.

Click Next when you are ready to progress.”
