# Supplementary material for: The development of a brief version of the Lexington Attachment to Pets Scale (Brief-LAPS)
Source: Front Vet Sci. 2025 Sep 2;12:1619187. doi: 10.3389/fvets.2025.1619187 (PMC12439530; doi:10.3389/fvets.2025.1619187)
Supplement: Supplementary file 3 [file Supplementary_file_3.docx]

**Supplementary file 3**

**Overview of questionnaires in language-specific versions**

Text with red font (filter instructions) and **blue-shaded text** were not presented to the respondents.

Page 2-39: Entire questionnaire in Danish

Page 40-79: Entire questionnaire in English

Page 80-122: Entire questionnaire in German

| **Indledning og baggrund:**  **Tak fordi du vil deltage i denne undersøgelse.**  **Formålet** med denne undersøgelse er at **finde ud af, hvorfor nogle folk har og andre ikke har kæledyr,** og at **undersøge kæledyrsejeres holdninger til dyrlægebehandling.** Det tager **ca. tre minutter at udfylde spørgeskemaet**, hvis du **ikke har kæledyr**. **Hvis du har kæledyr**, vil vi også spørge ind til **din tilknytning til dit/dine kæledyr**, din **holdning til moderne dyrlægepraksis** og dine **forventninger til de ydelser, du modtager hos dyrlægen**. Der er også spørgsmål vedrørende sundhedsforsikring til kæledyr samt brugen af sociale medier og internettet i relation til dyrlægebehandling. Det tager **ca. 15-20 minutter for kæledyrsejere at udfylde spørgeskemaet**.    Spørgeskemaet er en del af et fælles forskningsprojekt med tre deltagende lande: Danmark, Østrig og Storbritannien. De respektive forskere er fra Københavns Universitet, Det Veterinærmedicinske Universitet i Wien og University of Glasgow.  Det er **frivilligt at deltage** i undersøgelsen, og du kan **til enhver tid afbryde, før du har indsendt dine svar**. Dine svar videregives til forskerne i **anonymiseret** form, og **ingen oplysninger kan spores tilbage til dig**.  Når du trykker på ”Næste”, bekræfter du, at du er over 17 år gammel, og at du indvilliger i at deltage i undersøgelsen.  **Tak for dit bidrag!** |
| --- |
| **AFSNIT A:**  **Oplysninger om dit kæledyr, demografiske oplysninger samt oplysninger om den dyrlægepraksis/-klinik, du anvender.**  **Instruction A.1: single-choice question**  **A.1: Kæledyr holdes ofte i hjemmet, men vi er også interesserede i dyr, som ikke holdes i hjemmet (f.eks. heste). Vi er dog ikke interesserede i produktionsdyr, som typisk holdes på gårde, f.eks. malkekøer.**  **Har du et eller flere kæledyr?**   \| Ja \|  \| \| --- \| --- \| \| Nej \|  \|   **Instruction A.2: multiple choice question and only if A1=Ja**  **A.2: Hvor mange af de følgende kæledyr har du? Hvis du ikke har et kæledyr af den pågældende art, så afkryds venligst ”0 (ingen)”**   \|  \| 0 (ingen) \| 1 \| 2 \| 3 \| 4 \| Mere end 4 \| \| --- \| --- \| --- \| --- \| --- \| --- \| --- \| \| Hund \|  \|  \|  \|  \|  \|  \| \| Kat \|  \|  \|  \|  \|  \|  \| \| Hest \|  \|  \|  \|  \|  \|  \| \| Kanin \|  \|  \|  \|  \|  \|  \| \| Gnaver (f.eks. hamster, marsvin, chinchilla, mus/rotte) \|  \|  \|  \|  \|  \|  \| \| Fugl \|  \|  \|  \|  \|  \|  \| \| Krybdyr (f.eks. firben, slange, skildpadde) \|  \|  \|  \|  \|  \|  \| \| Fisk (både akvariefisk og fisk i en havedam) \|  \|  \|  \|  \|  \|  \| \| Andre dyr \|  \|  \|  \|  \|  \|  \|   *Construct filter variable =* ***Number of pet species***  *Count =* ***Number of pet species*** *if A2Hund>0, A2Kat>0, A2Hest>0, A2Kanin>0, A2Gnavert>0, A2Fugl>0, A2Krybdyr>0, A2Fisk>0, A2* Andre dyr *>0*  **Instruction A.3.1: only if A2Hund=1**  **A.3.1: Angiv venligst din hunds alder, antal besøg hos dyrlægen de seneste 12 måneder, samt hvorvidt hunden er dækket af en sundhedsforsikring.**  Scroll-down menu: alder (<0,5 - >20 år)  Scroll-down menu: antal besøg hos dyrlægen de seneste 12 måneder (0 - > 20)  Scroll-down menu: sundhedsforsikring (ja, nej, ikke længere)  **Instruction A.3.1.1: Danish questionnaire: single-choice question and only if A3.1 sundhedsforsikring=Ja**  **A.3.1.1: Hvor stor dækning er der på sundhedsforsikringen for din hund?**  Scroll-down menu: (ved ikke, op til 20.000 kr. årligt, 20.001-29.999 kr. årligt, 30.000-39.999 kr. årligt, 40.000-49.999 kr. årligt, mere end 50.000 kr. årligt)  **Instruction A.3.2: only if A2Hund=2**  **A.3.2: Angiv venligst alderen på dine hunde, antal besøg hos dyrlægen de seneste 12 måneder, samt hvorvidt de to hunde er dækket af en sundhedsforsikring.**  **A.3.2.1:**  Scroll-down menu: alder (<0,5 - >20 år)  Scroll-down menu: antal besøg hos dyrlægen de seneste 12 måneder (0 - > 20)  Scroll-down menu: sundhedsforsikring (ja, nej, ikke længere)  **A.3.2.2:**  Scroll-down menu: alder (<0,5 - >20 år)  Scroll-down menu: antal besøg hos dyrlægen de seneste 12 måneder (0 - > 20)  Scroll-down menu: sundhedsforsikring (ja, nej, ikke længere)  **Instruction A.3.3: only if A2Hund=3**  **A.3.3: Angiv venligst alderen på dine hunde, antal besøg hos dyrlægen de seneste 12 måneder, samt hvorvidt de tre hunde er dækket af en sundhedsforsikring.**  **A.3.3.1:**  Scroll-down menu: alder (<0,5 - >20 år)  Scroll-down menu: antal besøg hos dyrlægen de seneste 12 måneder (0 - > 20)  Scroll-down menu: sundhedsforsikring (ja, nej, ikke længere)  **A.3.3.2:**  Scroll-down menu: alder (<0,5 - >20 år)  Scroll-down menu: antal besøg hos dyrlægen de seneste 12 måneder (0 - > 20)  Scroll-down menu: sundhedsforsikring (ja, nej, ikke længere)  **A.3.3.3:**  Scroll-down menu: alder (<0,5 - >20 år)  Scroll-down menu: antal besøg hos dyrlægen de seneste 12 måneder (0 - > 20)  Scroll-down menu: sundhedsforsikring (ja, nej, ikke længere)  **Instruction A.3.4: only if A2Hund>3**  **A.3.4: Vi spørger nu ind til alderen på tre af dine hunde, antal besøg hos dyrlægen de seneste 12 måneder, samt hvorvidt de tre hunde er dækket af en sundhedsforsikring. Vælg venligst de tre hunde, hvis navne kommer først i alfabetet.**  **A.3.4.1:** Første hund (navnets begyndelsesbogstav kommer først i alfabetet)  Scroll-down menu: alder (<0,5 - >20 år)  Scroll-down menu: antal besøg hos dyrlægen de seneste 12 måneder (0 - > 20)  Scroll-down menu: sundhedsforsikring (ja, nej, ikke længere)  **A.3.4.2:** Anden hund (navnets begyndelsesbogstav kommer som næste i alfabetet)  Scroll-down menu: alder (<0,5 - >20 år)  Scroll-down menu: antal besøg hos dyrlægen de seneste 12 måneder (0 - > 20)  Scroll-down menu: sundhedsforsikring (ja, nej, ikke længere)  **A.3.4.3:** Tredje hund (navnets begyndelsesbogstav kommer som tredje i alfabetet)  Scroll-down menu: alder (<0,5 - >20 år)  Scroll-down menu: antal besøg hos dyrlægen de seneste 12 måneder (0 - > 20)  Scroll-down menu: sundhedsforsikring (ja, nej, ikke længere)  **Instruction A.3.5: Danish questionnaire: only if A.3.2.1** **sundhedsforsikring=Ja or A.3.2.2 sundhedsforsikring=Ja or A.3.3.1sundhedsforsikring=Ja or A3.3.2sundhedsforsikring=Ja or A3.3.3sundhedsforsikring=Ja or A.3.4.1sundhedsforsikring=Ja or A3.4.2sundhedsforsikring=Ja or A3.4.3sundhedsforsikring=Ja**  **A.3.5: Hvor stor dækning er der på sundhedsforsikringen for din hund/dine hunde? *Hvis dækningen ikke er den samme for alle hunde, så tænk på den hund, hvis navn kommer først i alfabetet.***  **Danmark:**  Scroll-down menu: (ved ikke, op til 20.000 kr. årligt, 20.001-29.999 kr. årligt, 30.000-39.999 kr. årligt, 40.000-49.999 kr. årligt, mere end 50.000 kr. årligt)  **Instruction A.3.6: only if A2Kat=1**  **A.3.6: Angiv venligst din kats alder, antal besøg hos dyrlægen de seneste 12 måneder, samt hvorvidt katten er dækket af en sundhedsforsikring.**  Scroll-down menu: alder (<0,5 - >20 år)  Scroll-down menu: antal besøg hos dyrlægen de seneste 12 måneder (0 - > 20)  Scroll-down menu: sundhedsforsikring (ja, nej, ikke længere)  **Instruction A.3.6.1: Danish questionnaire: single-choice question and only if A3.6 sundhedsforsikring=Ja**  **A.3.6.1: Hvor stor dækning er der på sundhedsforsikringen for din kat?**    Scroll-down menu: (ved ikke, op til 20.000 kr. årligt, 20.001-29.999 kr. årligt, 30.000-39.999 kr. årligt, 40.000-49.999 kr. årligt, mere end 50.000 kr. årligt)  **Instruction A.3.7: only if A2Kat=2**  **A.3.7: Angiv venligst alderen på dine katte, antal besøg hos dyrlægen de seneste 12 måneder, samt hvorvidt de to katte er dækket af en sundhedsforsikring.**  **A.3.7.1:**  Scroll-down menu: alder (<0,5 - >20 år)  Scroll-down menu: antal besøg hos dyrlægen de seneste 12 måneder (0 - > 20)  Scroll-down menu: sundhedsforsikring (ja, nej, ikke længere)  **A.3.7.2:**  Scroll-down menu: alder (<0,5 - >20 år)  Scroll-down menu: antal besøg hos dyrlægen de seneste 12 måneder (0 - > 20)  Scroll-down menu: sundhedsforsikring (ja, nej, ikke længere)  **Instruction A3.8: only if A2Kat=3**  **A.3.8: Angiv venligst alderen på dine katte, antal besøg hos dyrlægen de seneste 12 måneder, samt hvorvidt de tre katte er dækket af en sundhedsforsikring.**  **A.3.8.1:**  Scroll-down menu: alder (<0,5 - >20 år)  Scroll-down menu: antal besøg hos dyrlægen de seneste 12 måneder (0 - > 20)  Scroll-down menu: sundhedsforsikring (ja, nej, ikke længere)  **A.3.8.2:**  Scroll-down menu: alder (<0,5 - >20 år)  Scroll-down menu: antal besøg hos dyrlægen de seneste 12 måneder (0 - > 20)  Scroll-down menu: sundhedsforsikring (ja, nej, ikke længere)  **A.3.8.3:**  Scroll-down menu: alder (<0,5 - >20 år)  Scroll-down menu: antal besøg hos dyrlægen de seneste 12 måneder (0 - > 20)  Scroll-down menu: sundhedsforsikring (ja, nej, ikke længere)  **Instruction A.3.9: only if A2Kat>3**  **A.3.9: Vi spørger nu ind til alderen på tre af dine katte, antal besøg hos dyrlægen de seneste 12 måneder, samt hvorvidt de tre katte er dækket af en sundhedsforsikring. Vælg venligst de tre katte, hvis navne kommer først i alfabetet.**  **A.3.9.1:** Første kat (navnets begyndelsesbogstav kommer først i alfabetet)  Scroll-down menu: alder (<0,5 - >20 år)  Scroll-down menu: antal besøg hos dyrlægen de seneste 12 måneder (0 - > 20)  Scroll-down menu: sundhedsforsikring (ja, nej, ikke længere)  **A.3.9.2:** Anden kat (navnets begyndelsesbogstav kommer først i alfabetet)  Scroll-down menu: alder (<0,5 - >20 år)  Scroll-down menu: antal besøg hos dyrlægen de seneste 12 måneder (0 - > 20)  Scroll-down menu: sundhedsforsikring (ja, nej, ikke længere)  **A.3.9.3:** Tredje kat (navnets begyndelsesbogstav kommer først i alfabetet)  Scroll-down menu: alder (<0,5 - >20 år)  Scroll-down menu: antal besøg hos dyrlægen de seneste 12 måneder (0 - > 20)  Scroll-down menu: sundhedsforsikring (ja, nej, ikke længere)  **Instruction A.3.10: Danish questionnaire: only if A.3.7.1sundhedsforsikring=Ja or A.3.7.2sundhedsforsikring=Ja or A.3.8.1sundhedsforsikring=Ja or A3.8.2sundhedsforsikring=Ja or A3.8.3sundhedsforsikring=Ja or A.3.9.1sundhedsforsikring=Ja or A3.9.2sundhedsforsikring=Ja or A3.9.3sundhedsforsikring=Ja**  **A.3.10: Hvor stor dækning er der på sundhedsforsikringen for din kat/dine katte? *Hvis dækningen ikke er den samme for alle katte, så tænk på den kat, hvis navn kommer først i alfabetet.***  **Danmark:**  Scroll-down menu: (ved ikke, op til 20.000 kr. årligt, 20.001-29.999 kr. årligt, 30.000-39.999 kr. årligt, 40.000-49.999 kr. årligt, mere end 50.000 kr. årligt)  **Instruction A.4: multiple choice question and only if A1=Nej**  **A.4: Hvorfor har du ikke hund eller kat?**   \| Jeg kan ikke lide hunde og/eller katte. \|  \| \| \| --- \| --- \| --- \| \| En person i min husstand kan ikke lide hunde og/eller katte. \|  \| \| \| Jeg har ikke tid til at tage mig af en hund og/eller en kat. \|  \| \| \| Jeg har ikke råd til at tage mig af en hund og/eller en kat. \|  \| \| \| Jeg/en person i min husstand har allergi, som vil blive påvirket. \|  \| \| \| Jeg må ikke have hund og/eller kat, hvor jeg bor. \|  \| \| \| Jeg bor i lejlighed, og det er ikke i orden at have en kat eller hund under de vilkår. \|  \| \| \| Jeg mener, at mennesker slet ikke bør have kæledyr, fordi det krænker dyrenes rettigheder. \|  \| \| \| Andre grunde. \|  \|   **Instruction A.4.1: åbent svar hvis A4=”Andre grunde”**  **Du er velkommen til at uddybe, hvorfor du ikke har en hund eller kat.**   \| Åbent svar:… \| \| --- \| \| Jeg har ikke mere at tilføje \|  \|   **Instruction A.5: drop-down menu from 18 - 100 years + “Jeg foretrækker ikke at svare”**  **A.5:** **Angiv venligst din nuværende alder i år i rullemenuen.**  ________ år gammel.  **Instruction A.6: single-choice question**  **A.6: Angiv venligst dit køn.**   \| Mand \|  \| \| --- \| --- \| \| Kvinde \|  \| \| Ingen af disse \|  \| \| Jeg foretrækker ikke at svare \|  \|   **Instruction A.7: single-choice question**  **A.7: Angiv venligst det område, du bor i:**  **For Danmark:**   \| Region Hovedstaden \|  \| \| --- \| --- \| \| Region Midtjylland \|  \| \| Region Nordjylland \|  \| \| Region Sjælland \|  \| \| Region Syddanmark \|  \|   **Instruction A.8: single-choice question**  **A.8: Arbejder du eller har du arbejdet inden for dyrlægebranchen (fx som praktiserende dyrlæge, veterinærsygeplejerske eller klinikassistent)?**   \| Ja \|  \| \| --- \| --- \| \| Nej \|  \|   **Instruction A.10.1: single-choice question**  **A.10.1: Bor du alene?** (du skal ikke tælle kæledyr med)   \| Ja \|  \| \| --- \| --- \| \| Nej \|  \|   **Instruction A.10.2: single-choice question and only if A10.1=Nej and list in drop-down-menu**  **A.10.2: Hvem bor du sammen med?**   \| Jeg bor sammen med en anden voksen/andre voksne \|  \| \| --- \| --- \| \| Jeg bor sammen med en anden voksen/andre voksne og et barn under 18 år (fuldtid) \|  \| \| Jeg bor sammen med en anden voksen/andre voksne og et barn under 18 år (deltid) \|  \| \| Jeg bor sammen med en anden voksen/andre voksne og børn under 18 år (fuldtid) \|  \| \| Jeg bor sammen med en anden voksen/andre voksne og børn under 18 år (deltid) \|  \| \| Jeg bor sammen med et barn under 18 år (fuldtid) \|  \| \| Jeg bor sammen med et barn under 18 år (deltid) \|  \| \| Jeg bor sammen med børn under 18 år (fuldtid) \|  \| \| Jeg bor sammen med børn under 18 år (deltid) \|  \| \| Andet \|  \|   **Instruction A11.1: single-choice question and only if A2=Hund & A10.2 = “Jeg bor sammen med en anden voksen/andre voksne** herunder mit/mine barn/børn, der er 18 år eller ældre**” or “Jeg bor sammen med en anden voksen/andre voksne og et barn (fuldtid)” or “Jeg bor sammen med en anden voksen/andre voksne og et barn (deltid)” or “Jeg bor sammen med en anden voksen/andre voksne og børn (fuldtid)” or “Jeg bor sammen med en anden voksen/andre voksne og børn (deltid)” or “Jeg bor sammen med et barn (fuldtid)” or “Jeg bor sammen med et barn (deltid)” or “Jeg bor sammen med børn (fuldtid)” or “Jeg bor sammen med børn (deltid)”**  **A.11.1: Hvem blev hunden/hundene anskaffet til?**   \| Alle (eller de fleste) i husstanden \|  \| \| --- \| --- \| \| Mig selv \|  \| \| Min partner/ægtefælle \|  \| \| Mit barn/stedbarn \|  \| \| Mine børn/stedbørn \|  \| \| Andet/andre dyr i husstanden (til selskab) \|  \| \| Andre \|  \| \| Ved ikke \|  \|   **Instruction A11.2: single-choice question and only if A2=Kat & A10.2 = “Jeg bor sammen med en anden voksen/andre voksne** herunder mit/mine barn/børn, der er 18 år eller ældre**” or “Jeg bor sammen med en anden voksen/andre voksne og et barn (fuldtid)” or “Jeg bor sammen med en anden voksen/andre voksne og et barn (deltid)” or “Jeg bor sammen med en anden voksen/andre voksne og børn (fuldtid)” or “Jeg bor sammen med en anden voksen/andre voksne og børn (deltid)” or “Jeg bor sammen med et barn (fuldtid)” or “Jeg bor sammen med et barn (deltid)” or “Jeg bor sammen med børn (fuldtid)” or “Jeg bor sammen med børn (deltid)”**  **A.11.2: Hvem blev katten/kattene anskaffet til?**   \| Alle (eller de fleste) i husstanden \|  \| \| --- \| --- \| \| Mig selv \|  \| \| Min partner/ægtefælle \|  \| \| Mit barn/stedbarn \|  \| \| Mine børn/stedbørn \|  \| \| Andet/andre dyr i husstanden (til selskab) \|  \| \| Andre \|  \| \| Ved ikke \|  \|   **Instruction A12.1: single-choice question and only if A2=Hund & A10.2 = “Jeg bor sammen med en anden voksen/andre voksne** herunder mit/mine barn/børn, der er 18 år eller ældre**” or “Jeg bor sammen med en anden voksen/andre voksne og et barn (fuldtid)” or “Jeg bor sammen med en anden voksen/andre voksne og et barn (deltid)” or “Jeg bor sammen med en anden voksen/andre voksne og børn (fuldtid)” or “Jeg bor sammen med en anden voksen/andre voksne og børn (deltid)” or “Jeg bor sammen med et barn (fuldtid)” or “Jeg bor sammen med et barn (deltid)” or “Jeg bor sammen med børn (fuldtid)” or “Jeg bor sammen med børn (deltid)”**  **A.12.1: Hvem i husstanden, mener du, er mest knyttet til hunden/hundene?**   \| Alle (eller de fleste) i husstanden \|  \| \| --- \| --- \| \| Mig selv \|  \| \| Min partner/ægtefælle \|  \| \| Mit barn/stedbarn \|  \| \| Mine børn/stedbørn \|  \| \| Andre \|  \| \| Ved ikke \|  \|   **Instruction A12.2: single-choice question and only if A2=Kat & A10.2 = “Jeg bor sammen med en anden voksen/andre voksne** herunder mit/mine barn/børn, der er 18 år eller ældre**” or “Jeg bor sammen med en anden voksen/andre voksne og et barn (fuldtid)” or “Jeg bor sammen med en anden voksen/andre voksne og et barn (deltid)” or “Jeg bor sammen med en anden voksen/andre voksne og børn (fuldtid)” or “Jeg bor sammen med en anden voksen/andre voksne og børn (deltid)” or “Jeg bor sammen med et barn (fuldtid)” or “Jeg bor sammen med et barn (deltid)” or “Jeg bor sammen med børn (fuldtid)” or “Jeg bor sammen med børn (deltid)”**  **A.12.2: Hvem i husstanden, mener du, er mest knyttet til katten/kattene?**   \| Alle (eller de fleste) i husstanden \|  \| \| --- \| --- \| \| Mig selv \|  \| \| Min partner/ægtefælle \|  \| \| Mit barn/stedbarn \|  \| \| Mine børn/stedbørn \|  \| \| Andre \|  \| \| Ved ikke \|  \|   **Instruction A13.1: multiple choice question and only if A2=Hund**  **A.13.1: Hvorfor blev hunden/hundene anskaffet? Vælg alle de muligheder, der er relevante for dig.**   \| Som selskab til mig (eller andre personer) \|  \| \| --- \| --- \| \| Som selskab til andre dyr \|  \| \| Til at avle på \|  \| \| Til at udstille ved konkurrencer/udstillinger \|  \| \| Til hundesport (træning, agility osv.) \|  \| \| Til at motionere med (gåture) \|  \| \| Som jagthund \|  \| \| Til at holde øje med hjemmet og/eller ejendommen \|  \| \| Andet \|  \| \| Ved ikke \|  \|   **Instruction A13.2: multiple choice question and only if A2=Kat**  **A.13.2: Hvorfor blev katten/kattene anskaffet? Vælg alle de muligheder, der er relevante for dig.**   \| Som selskab til mig (eller andre personer) \|  \| \| --- \| --- \| \| Som selskab til andre dyr \|  \| \| Til at avle på \|  \| \| Til at udstille ved konkurrencer/udstillinger \|  \| \| Til at holde mus og rotter væk fra hjemmet \|  \| \| Den/de dukkede bare op og er blevet her \|  \| \| Andet \|  \| \| Ved ikke \|  \|   **Instruction 14.1: only if**  **Number of pet species=1 & A2Hund=1**  **Number of pet species=1 & A2Kat=1**  **Number of pet species=1 & A2Hest=1**  **Number of pet species=1 & or A2Kanin=1**  **Number of pet species=1 & A2Gnaver=1**  **Number of pet species=1 & A2Fugl=1**  **Number of pet species=1 & A2Krybdyr=1**  **Number of pet species=1 & A2Fisk=1**  **Number of pet species=1 & A2 Andre dyr =1**  **A14.1: Tænk på dit kæledyr og angiv, hvorvidt du er helt uenig, delvist uenig, delvist enig eller helt enig i de følgende udsagn.**  *Please use answer options /matrix provided under question A.14.11.1*  **Instruction 14.2: only if Number of pet species=1 & A2Hund>1**  **A14.2: Tænk på din yndlingshund og angiv, hvorvidt du er helt uenig, delvist uenig, delvist enig eller helt enig i de følgende udsagn.**  *Please use answer options /matrix provided under question A.14.11.1*  **Instruction 14.3: only if Number of pet species=1 & A2Kat>1**  **A14.3: Tænk på din yndlingskat og angiv, hvorvidt du er helt uenig, delvist uenig, delvist enig eller helt enig i de følgende udsagn.**  *Please use answer options /matrix provided under question A.14.11.1*  **Instruction 14.4: only if Number of pet species=1 & A2Hest>1**  **A14.4: Tænk på din yndlingshest og angiv, hvorvidt du er helt uenig, delvist uenig, delvist enig eller helt enig i de følgende udsagn.**  *Please use answer options /matrix provided under question A.14.11.1*  **Instruction 14.5: only if Number of pet species=1 & A2Kanin>1**  **A14.5: Tænk på din yndlingskanin og angiv, hvorvidt du er helt uenig, delvist uenig, delvist enig eller helt enig i de følgende udsagn.**  *Please use answer options /matrix provided under question A.14.11.1*  **Instruction 14.6: only if Number of pet species=1 & A2Gnaver>1**  **A14.6: Tænk på din yndlingsgnaver og angiv, hvorvidt du er helt uenig, delvist uenig, delvist enig eller helt enig i de følgende udsagn.**  *Please use answer options /matrix provided under question A.14.11.1*  **Instruction 14.7: only if Number of pet species=1 & A2Fugl>1**  **A14.7: Tænk på din yndlingsfugl og angiv, hvorvidt du er helt uenig, delvist uenig, delvist enig eller helt enig i de følgende udsagn.**  *Please use answer options /matrix provided under question A.14.11.1*  **Instruction 14.8: only if Number of pet species=1 & A2Krybdyr>1**  **A14.8: Tænk på dit yndlingskrybdyr og angiv, hvorvidt du er helt uenig, delvist uenig, delvist enig eller helt enig i de følgende udsagn.**  *Please use answer options /matrix provided under question A.14.11.1*  **Instruction 14.9: only if Number of pet species=1 & A2Fisk>1**  **A14.9: Tænk på din yndlingsfisk og angiv, hvorvidt du er helt uenig, delvist uenig, delvist enig eller helt enig i de følgende udsagn.**  *Please use answer options /matrix provided under question A.14.11.1*  **Instruction 14.10: only if Number of pet species=1 & A2** **Andre dyr >1**  **A14.10: Tænk på dit yndlingskæledyr og angiv, hvorvidt du er helt uenig, delvist uenig, delvist enig eller helt enig i de følgende udsagn.**  *Please use answer options /matrix provided under question A.14.11.1*  **Instruction A.14.11: only if Number of pet species>1**  **A14.11: Tænk på dit yndlingskæledyr. Hvilken art er det?**  *Please use answer options /matrix provided under question A.14.11.1*   \| Hund \|  \| \| --- \| --- \| \| Kat \|  \| \| Hest \|  \| \| Kanin \|  \| \| Gnaver (f.eks. hamster, marsvin, chinchilla, mus/rotte) \|  \| \| Fugl \|  \| \| Krybdyr (f.eks. firben, slange, skildpadde) \|  \| \| Fisk (både akvariefisk og fisk i en havedam) \|  \| \| Andre dyr \|  \|   **Instruction 14.11.1: only if Number of pet species>1**  **A14.11.1: Angiv venligst, hvorvidt du er helt uenig, delvist uenig, delvist enig eller helt enig i de følgende udsagn om dit yndlingskæledyr.**  *1=helt uenig, 2=delvist uenig, 3=delvist enig og 4=helt enig*   \|  \| **Tilknytning til mit kæledyr** \| **1** \| **2** \| **3** \| **4** \| \| --- \| --- \| --- \| --- \| --- \| --- \| \| 1 \| Mit kæledyr betyder mere for mig end mine venner gør. \|  \|  \|  \|  \| \| 2 \| Jeg betror mig ofte til mit kæledyr. \|  \|  \|  \|  \| \| 3 \| Jeg mener, at kæledyr skal have samme rettigheder og privilegier som familiemedlemmer. \|  \|  \|  \|  \| \| 4 \| Jeg mener, mit kæledyr er min bedste ven. \|  \|  \|  \|  \| \| 5 \| Jeg oplever ofte, at min opfattelse af andre mennesker påvirkes af, hvordan de reagerer på mit kæledyr. \|  \|  \|  \|  \| \| 6 \| Jeg elsker mit kæledyr, fordi han/hun er mere loyal over for mig end de fleste mennesker i mit liv. \|  \|  \|  \|  \| \| 7 \| Jeg kan godt lide at vise andre mennesker billeder af mit kæledyr. \|  \|  \|  \|  \| \| 8 \| Jeg synes, mit kæledyr bare er et kæledyr. \|  \|  \|  \|  \| \| 9 \| Jeg elsker mit kæledyr, fordi det aldrig dømmer mig. \|  \|  \|  \|  \| \| 10 \| Mit kæledyr kan mærke, når jeg har det dårligt. \|  \|  \|  \|  \| \| 11 \| Jeg taler ofte med andre mennesker om mit kæledyr. \|  \|  \|  \|  \| \| 12 \| Mit kæledyr forstår mig. \|  \|  \|  \|  \| \| 13 \| Jeg tror på, at kærligheden til mit kæledyr, hjælper mig med at holde mig sund og rask. \|  \|  \|  \|  \| \| 14 \| Kæledyr fortjener lige så meget respekt, som mennesker gør. \|  \|  \|  \|  \| \| 15 \| Mit kæledyr og jeg har et meget nært forhold. \|  \|  \|  \|  \| \| 16 \| Jeg vil gøre næsten alt for at tage mig af mit kæledyr. \|  \|  \|  \|  \| \| 17 \| Jeg leger ofte med mit kæledyr. \|  \|  \|  \|  \| \| 18 \| Jeg anser mit kæledyr for at være en fantastisk følgesvend. \|  \|  \|  \|  \| \| 19 \| Mit kæledyr gør mig glad. \|  \|  \|  \|  \| \| 20 \| Jeg føler, at mit kæledyr er en del af min familie. \|  \|  \|  \|  \| \| 21 \| Jeg er ikke særligt knyttet til mit kæledyr. \|  \|  \|  \|  \| \| 22 \| Det at eje et kæledyr gør mig glad. \|  \|  \|  \|  \| \| 23 \| Jeg anser mig kæledyr for at være en ven. \|  \|  \|  \|  \|   **Instruction A15.1: only if A2=Hund**  **A.15.1: Angiv venligst, hvilken type dyrlægepraksis du normalt besøger med din hund/dine hunde:**  **Instruction A15.2: only if A2=Kat**  **A.15.2: Angiv venligst, hvilken type dyrlægeklinik du normalt besøger med din kat/dine katte:**   \| En lille dyrlægeklinik (1-3 dyrlæger) \|  \| \| --- \| --- \| \| En større dyrlægeklinik (4 eller flere dyrlæger) \|  \| \| Dyrlægeklinik, hvor der også behandles andre typer af dyr (fx landbrugsdyr) \|  \| \| Dyrehospital på universitetet \|  \| \| Klinik, som hører under en dyreværnsorganisation \|  \| \| Jeg besøger forskellige kliniktyper \|  \| \| Jeg besøger slet ikke en dyrlægeklinik \|  \| \| Ved ikke \|  \|   **Instruction A.16: only if A2=Hund &/or A2=Kat**  **A.16: Angiv venligst, hvor enig eller uenig du er i de følgende udsagn angående din dyrlæge:**  1= helt uenig; 2 = uenig; 3 = delvist uenig; 4 = neutral (hverken enig eller uenig); 5 = delvist enig; 6 = enig og 7 = helt enig; 8=ved ikke   \|  \| **Det er vigtigt, at ...** \| 1 \| 2 \| 3 \| 4 \| 5 \| 6 \| 7 \| 8 \| \| --- \| --- \| --- \| --- \| --- \| --- \| --- \| --- \| --- \| --- \| \| 1 \| jeg kommer ind til samme dyrlæge hver gang. \|  \|  \|  \|  \|  \|  \|  \|  \| \| 2 \| jeg har et godt forhold til/kan lide min dyrlæge. \|  \|  \|  \|  \|  \|  \|  \|  \| \| 3 \| mit dyr kan lide/ser ud til at være tryg ved min dyrlæge. \|  \|  \|  \|  \|  \|  \|  \|  \| \| 4 \| min dyrlæge ser ud til at bekymre sig om mit kæledyr. \|  \|  \|  \|  \|  \|  \|  \|  \| \| 5 \| min dyrlæge har specialiseret sig ud over sin dyrlægeuddannelse. \|  \|  \|  \|  \|  \|  \|  \|  \| \| 6 \| min dyrlæge virker professionel. \|  \|  \|  \|  \|  \|  \|  \|  \| \| 7 \| jeg kan stole på min dyrlæge \|  \|  \|  \|  \|  \|  \|  \|  \|   **Instruction A.17.13: only if A2=Dog &/or A2=Cat**  **A.17: Hvor vigtige er de følgende aspekter for dig, når du vælger dyrlæge?**  *1= “Slet ikke vigtigt”; 2=”mindre vigtigt”; 3=” vigtigt”; 4=” meget vigtigt”; 5=” Ved ikke”*   \|  \| **Dyrlægeklinikken skal ...** \| **1** \| **2** \| **3** \| **4** \| **5** \| \| --- \| --- \| --- \| --- \| --- \| --- \| --- \| \| 1 \| - være tæt på, hvor jeg bor \|  \|  \|  \|  \|  \| \| 2 \| - tilbyde 24 timers behandling af de mest almindelige akutte ting (ikke sende mig et andet sted hen). \|  \|  \|  \|  \|  \| \| 3 \| - være tilgængelig med gode parkeringsmuligheder \|  \|  \|  \|  \|  \| \| 4 \| - tilbyde konsultationstider der passer ind i praktiske forhold \|  \|  \|  \|  \|  \| \| 5 \| - tage hensyn til artsspecifikke behov (f.eks. separat venteværelse til katte for at mindske stress). \|  \|  \|  \|  \|  \| \| 6 \| - tilbyde specialistbehandling hvis påkrævet, f.eks. udskiftning af led eller kræftbehandling. \|  \|  \|  \|  \|  \| \| 7 \| - tilbyde alternativ medicin, f.eks. traditionel kinesisk medicin eller homøopati. \|  \|  \|  \|  \|  \| \| 8 \| - have rimelige priser. \|  \|  \|  \|  \|  \| \| 9 \| - være privatejet. \|  \|  \|  \|  \|  \| \| 10 \| - være en del af en kæde, f.eks. AniCura eller Evidensia. \|  \|  \|  \|  \|  \| \| 11 \| - tilbyde hjemmebesøg. \|  \|  \|  \|  \|  \| \| 12 \| - tilbyde afdragsordning. \|  \|  \|  \|  \|  \| \| 13 \| - afregne direkte med forsikringen. \|  \|  \|  \|  \|  \|   **Instruction A.18: multiple-choice question and only if A2=Hund &/or A2=Kat**  **A.18: Hvilke af de følgende diagnostik- og behandlingsmuligheder, forventer du, er tilgængelige i den klinik, du normalt besøger? Vælg alle de muligheder, der gælder for dig.**   \| **Radiografi** (røntgenbilleder) \|  \| \| --- \| --- \| \| **Ultralyd** (en billeddannelsesteknik, som normalt anvendes i maven) \|  \| \| **Endoskopi** (et kamera, som ser ind i kroppen og fjerner fremmedlegemer fra passager såsom maven og luftvejene - muliggør kikkertoperationer) \|  \| \| **Artroskopi** (et kamera, som ser ind i led) \|  \| \| **Eget laboratorium** (til f.eks. blodprøver og urinanalyse) \|  \| \| **Tandudstyr** \|  \| \| **MR-scanner** (en avanceret form for billeddiagnostik, som normalt anvendes til rygsøjlen) \|  \| \| **CT-scanner** (en avanceret form for billeddiagnostik, som normalt anvendes til brystkassen) \|  \| \| **Ingen af de ovenstående** \|  \| \| **Ved ikke** \|  \|   **Instruction A.19.1: single-choice question and only if A2=Hund**  **A.19.1: Har du nogensinde været hos en specialdyrlæge (f.eks. en neurolog, adfærdsterapeut, eller onkolog) med din hund/dine hunde?**   \| Ja \|  \| \| --- \| --- \| \| Nej \|  \| \| Ved ikke \|  \|   **Instruction A.19.2: single-choice question and only if A2=Kat**  **A.19.2: Har du nogensinde været hos en specialdyrlæge (f.eks. en neurolog, adfærdsterapeut, eller onkolog) med din kat/dine katte?**   \| Ja \|  \| \| --- \| --- \| \| Nej \|  \| \| Ved ikke \|  \|   **Instruction A.20.1: single-choice question and only if A2=Hund & A.3.1-A.3.4sundhedsforsikring=Nej or A.3.1-A3.4sundhedsforsikring=ikke længere**  **A.20.1: Hvis (en af) dine uforsikrede hunde led af alvorlig sygdom og enten skulle i behandling (med gode chancer for et godt udfald) eller aflives, hvad ville du så gøre?**  **Instruction A.20.2: single-choice question and only if A2=Kat & A.3.6-A.3.9 sundhedsforsikring=Nej or A.3.6-A3.9sundhedsforsikring=ikke længere**  **A.20.2: Hvis (en af) dine uforsikrede katte led af alvorlig sygdom og enten skulle i behandling (med gode chancer for et godt udfald) eller aflives, hvad ville du så gøre?**   \| **Jeg ville anmode om aflivning.** \|  \| \| --- \| --- \| \| **Jeg ville bruge op til 999 kr. på behandling.** \|  \| \| **Jeg ville bruge 1.000-1.999 kr. på behandling.** \|  \| \| **Jeg ville bruge 2.000-4.999 kr. på behandling.** \|  \| \| **Jeg ville bruge 5.000-9.999 kr. på behandling.** \|  \| \| **Jeg ville bruge 10.000-29.999 kr. på behandling.** \|  \| \| **Jeg ville bruge 30.000-59.999 kr. på behandling.** \|  \| \| **Jeg ville bruge 60.000-99.999 kr. på behandling.** \|  \| \| **Jeg ville bruge 100.000 kr. eller derover på behandling.** \|  \| \| **Ved ikke** \|  \|   **Instruction A.21.1: single-choice question and only if A2=Dog & A.3.1-A.3.4sundhedsforsikring=Ja**  **A.21.1: Hvis (en af) dine forsikrede hunde led af alvorlig sygdom og enten skulle i behandling (med gode chancer for et godt udfald) eller aflives, hvad ville du så gøre?**  **Instruction A.21.2: single-choice question and only if A2=Cat & A.3.6-A.3.9sundhedsforsikring=Ja**  **A.21.2: Hvis (en af) dine forsikrede katte led af alvorlig sygdom og enten skulle i behandling (med gode chancer for et godt udfald) eller aflives, hvad ville du så gøre?**   \| **Jeg ville anmode om aflivning.** \|  \| \| --- \| --- \| \| **Jeg ville bruge op til 999 kr. mere end forsikringen dækker** \|  \| \| **Jeg ville bruge 1.000-1.999 kr. mere end forsikringen dækker** \|  \| \| **Jeg ville bruge 2.000-4.999 kr. mere end forsikringen dækker** \|  \| \| **Jeg ville bruge 5.000-9.999 kr. mere end forsikringen dækker** \|  \| \| **Jeg ville bruge 10.000-29.999 kr. mere end forsikringen dækker** \|  \| \| **Jeg ville bruge 30.000-59.999 kr. mere end forsikringen dækker** \|  \| \| **Jeg ville bruge 60.000-99.999 kr. mere end forsikringen dækker** \|  \| \| **Jeg ville bruge 100.000 kr. eller derover mere end forsikringen dækker** \|  \| \| **Ved ikke** \|  \| |
| **Instruction Section B: only if A2=Hund & A2=Kat**  **AFSNIT B:**  **Anden del af spørgeskemaet fokuserer på de avancerede dyrlægebehandlinger, som er tilgængelige i moderne veterinærpraksis.**    **B.1: Udviklingen i praksis med kæledyr**   \|  \|  \| 1 \| 2 \| 3 \| 4 \| 5 \| 6 \| 7 \| \| --- \| --- \| --- \| --- \| --- \| --- \| --- \| --- \| --- \| \| 1 \| Mit kæledyr skal have adgang til de samme **undersøgelser,** som er tilgængelige for mennesker. \|  \|  \|  \|  \|  \|  \|  \| \| 2 \| Mit kæledyr skal have adgang til de samme **behandlingsmuligheder,** som er tilgængelige for mennesker. \|  \|  \|  \|  \|  \|  \|  \| \| 3 \| Jeg tilmelder gerne mit kæledyr til et forskningsprojekt for at bidrage til fremtidige behandlingsmuligheder, så længe risikoen for potentielle bivirkninger er lav. \|  \|  \|  \|  \|  \|  \|  \| \| 4 \| Min dyrlæge skal tilbyde mit kæledyr den mest avancerede behandling. \|  \|  \|  \|  \|  \|  \|  \| \| 5 \| Det er vigtigt, at min dyrlæge bidrager til udviklingen af behandlingsmuligheder til gavn for fremtidige patienter. \|  \|  \|  \|  \|  \|  \|  \| \| 6 \| De avancerede behandlingsmuligheder inden for moderne dyrlægepraksis er gået ”for vidt”, og dyrene udsættes for ”alt for meget”. \|  \|  \|  \|  \|  \|  \|  \| \| 7 \| De avancerede behandlingsmuligheder inden for moderne dyrlægepraksis er unødvendige - dyr skal ikke behandles på samme måde som mennesker. \|  \|  \|  \|  \|  \|  \|  \|   **B.1: Angiv venligst, hvor enig eller uenig du er i de følgende udsagn:**  1= helt uenig; 2 = uenig; 3 = delvist uenig; 4 = neutral (hverken enig eller uenig); 5 = delvist enig; 6 = enig og 7 = helt enig |
| **B.2: Faktorer, som relaterer sig til patienten, kunden og dyrlægens professionelle omgivelser**  **B.2: Angiv venligst, hvor enig eller uenig du er i de følgende udsagn angående din dyrlæge:**  1= helt uenig; 2 = uenig; 3 = delvist uenig; 4 = neutral (hverken enig eller uenig); 5 = delvist enig; 6 = enig og 7 = helt enig   \|  \|  \| 1 \| 2 \| 3 \| 4 \| 5 \| 6 \| 7 \| 8 \| \| --- \| --- \| --- \| --- \| --- \| --- \| --- \| --- \| --- \| --- \| \| 1 \| Mit kæledyr skal altid prioriteres af min dyrlæge, når han/hun tager beslutninger. \|  \|  \|  \|  \|  \|  \|  \|  \| \| 2 \| Hvis mit kæledyr er uhelbredeligt sygt, skal min dyrlæge være villig til at yde lindrende behandling, så jeg har tid til at sige farvel. \|  \|  \|  \|  \|  \|  \|  \|  \| \| 3 \| Min dyrlæge skal diskutere alle relevante behandlingsmuligheder med mig, men beslutningen ligger hos mig. \|  \|  \|  \|  \|  \|  \|  \|  \| \| 4 \| Min dyrlæge skal tage hensyn til mine følelser, når der tages stilling til behandlingen. \|  \|  \|  \|  \|  \|  \|  \|  \| \| 5 \| Min dyrlæge skal opfordre mig til at vælge den bedste behandling til mit kæledyr, selv hvis det sætter mig under pres, f.eks. tidsmæssigt eller økonomisk. \|  \|  \|  \|  \|  \|  \|  \|  \| \| 6 \| Min dyrlæge skal tage hensyn til mine personlige forhold (f.eks. økonomi, tid til rådighed, levevilkår) i beslutningstagningsprocessen, selv hvis det betyder, at han/hun dermed ikke kan give mit kæledyr den bedst mulige behandling. \|  \|  \|  \|  \|  \|  \|  \|  \| \| 7 \| Min dyrlæge skal kun tage beslutninger, som er i mit kæledyrs bedste interesse, selv hvis det betyder, at jeg bliver sendt til en anden dyrlæge. \|  \|  \|  \|  \|  \|  \|  \|  \| \| 8 \| Det er min dyrlæges ansvar at finde den bedst mulige løsning for mit kæledyr *og* mig. \|  \|  \|  \|  \|  \|  \|  \|  \| \| 9 \| Min dyrlæge skal ikke kun opføre sig professionelt, men også yde følelsesmæssig støtte. \|  \|  \|  \|  \|  \|  \|  \|  \| \| 10 \| Hvis min dyrlæges mening adskiller sig fra min, skal han/hun lade mig tage beslutningen i sidste ende. \|  \|  \|  \|  \|  \|  \|  \|  \| \| 11 \| Det er vigtigere, at min dyrlæge handler i mit kæledyrs bedste interesse, end at han/hun tager hensyn til mine bekymringer (f.eks. økonomiske problemer). \|  \|  \|  \|  \|  \|  \|  \|  \| \| 12 \| Hvis der er en konflikt, så skal min dyrlæge udvise empati over for mine personlige følelser, når der tages stilling til behandlingen. \|  \|  \|  \|  \|  \|  \|  \|  \| \| 13 \| Min dyrlæge skal ikke tage beslutninger – jeg vil tage beslutningen på vegne af mit kæledyr. \|  \|  \|  \|  \|  \|  \|  \|  \| \| 14 \| Min dyrlæge skal udvise empati over for min situation, når der tages stilling til behandlingen. \|  \|  \|  \|  \|  \|  \|  \|  \| \| 15 \| Min dyrlæge skal være parat til at gå på kompromis med behandlingen, hvis jeg ikke har råd til den bedst mulige/mest relevante behandling. \|  \|  \|  \|  \|  \|  \|  \|  \| |
| **B.2.1: Råd fra dyrlægen**  **Instruction B.2.1: single-choice question**  **B.2.1:** **Har du været i en situation, hvor du ville fortsætte en behandling af dit kæledyr imod din dyrlæges råd?**   \| Ja \|  \| \| --- \| --- \| \| Nej \|  \| \| Ved ikke \|  \|   **Instruction B.2.2: only if B.2.1=Ja**  **B.2.2: Angiv venligst, i hvilken grad de følgende faktorer påvirkede dit ønske om at fortsætte behandlingen mod din dyrlæges råd:**  1= slet ikke; 2 = i mindre grad; 3 = moderat; 4 = i nogen grad; 5 = i høj grad og 6 = ved ikke   \|  \|  \| 1 \| 2 \| 3 \| 4 \| 5 \| 6 \| \| --- \| --- \| --- \| --- \| --- \| --- \| --- \| --- \| \| 1 \| Jeg kender mit kæledyr bedre, end dyrlægen gør. \|  \|  \|  \|  \|  \|  \| \| 2 \| Pres fra en anden person (f.eks. familiemedlem, ven). \|  \|  \|  \|  \|  \|  \| \| 3 \| Jeg fandt relevante oplysninger på internettet. \|  \|  \|  \|  \|  \|  \| \| 4 \| Jeg mener/mente, at det var i mit kæledyrs bedste interesse. \|  \|  \|  \|  \|  \|  \| \| 5 \| Jeg har haft/har hørt om positive erfaringer med et andet dyr, som fik samme behandling. \|  \|  \|  \|  \|  \|  \| \| 6 \| Jeg har haft/har hørt om positive erfaringer fra familie eller venner, som gennemgik samme behandling. \|  \|  \|  \|  \|  \|  \| \| 7 \| Jeg har set positive beretninger om behandlingen i medierne og på sociale medier. \|  \|  \|  \|  \|  \|  \| \| 8 \| Jeg fik en second opinion hos en anden dyrlæge. \|  \|  \|  \|  \|  \|  \| \| 9 \| Mit kæledyr var forsikret. \|  \|  \|  \|  \|  \|  \|   **Instruction B.2.3: single-choice question**  **B.2.3: Har du været i en situation, hvor du afviste en behandling af dit kæledyr, selvom din dyrlæge anbefalede den?**   \| Ja \|  \| \| --- \| --- \| \| Nej \|  \| \| Ved ikke \|  \|   **Instruction B.2.4: only if B.2.3=Ja**  **B.2.4: Angiv venligst, i hvilken grad de følgende faktorer påvirkede valget, du tog, om at afvise behandlingen, selvom din dyrlæge anbefalede den:**  1= slet ikke; 2 = i mindre grad; 3 = moderat; 4 = i nogen grad; 5 = i høj grad og 6 = ved ikke   \|  \|  \| 1 \| 2 \| 3 \| 4 \| 5 \| 6 \| \| --- \| --- \| --- \| --- \| --- \| --- \| --- \| --- \| \| 1 \| Jeg havde ikke råd til behandlingen på det tidspunkt. \|  \|  \|  \|  \|  \|  \| \| 2 \| Jeg følte, at mit kæledyr var for gammelt. \|  \|  \|  \|  \|  \|  \| \| 3 \| Jeg var ikke stærkt følelsesmæssigt tilknyttet til mit kæledyr. \|  \|  \|  \|  \|  \|  \| \| 4 \| Pres fra en anden person (f.eks. familiemedlem, ven). \|  \|  \|  \|  \|  \|  \| \| 5 \| Jeg har/havde begrænset tid til at yde den påkrævede omsorg. \|  \|  \|  \|  \|  \|  \| \| 6 \| Jeg mente ikke, at behandlingen var i mit kæledyrs bedste interesse. \|  \|  \|  \|  \|  \|  \| \| 7 \| Jeg har haft/har hørt om dårlige erfaringer med et andet dyr, som gennemgik samme behandling. \|  \|  \|  \|  \|  \|  \| \| 8 \| Jeg har haft/har hørt om dårlige erfaringer fra familie eller venner, som gennemgik samme behandling. \|  \|  \|  \|  \|  \|  \| \| 9 \| Jeg har set negative beretninger om behandlingen i medierne og på sociale medier. \|  \|  \|  \|  \|  \|  \| \| 10 \| Jeg ville have en second opinion hos en anden dyrlæge. \|  \|  \|  \|  \|  \|  \| |
| **B.3: Sundhedsforsikring i veterinærpraksis**  **Instruction B.3.1: multiple choice question and only if A3.1sundhedsforsikring=Ja or A.3.2.1sundhedsforsikring=Ja or A.3.2.2sundhedsforsikring=Ja or A.3.3.1sundhedsforsikring=Ja or A3.3.2sundhedsforsikring=Ja or A3.3.3 sundhedsforsikring=Ja or A.3.4.1sundhedsforsikring=Ja or A3.4.2sundhedsforsikring=Ja or A3.4.3sundhedsforsikring=Ja or A.3.6sundhedsforsikring=Ja or A.3.7.1sundhedsforsikring=Ja or A.3.7.2sundhedsforsikring=Ja or A.3.8.1sundhedsforsikring=Ja or A3.8.2sundhedsforsikring=Ja or A3.8.3sundhedsforsikring=Ja or A.3.9.1sundhedsforsikring=Ja or A3.9.2sundhedsforsikring=Ja or A3.9.3sundhedsforsikring=Ja**  **B.3.1 Hvorfor har du tegnet en sundhedsforsikring til dit kæledyr/dine kæledyr? Vælg alle de muligheder, der gælder for dig.**   \| 1 \| Min dyrlæge rådede mig til at tegne en sundhedsforsikring. \|  \| \| --- \| --- \| --- \| \| 2 \| Jeg har tidligere haft sundhedsforsikring til kæledyr. \|  \| \| 3 \| Jeg har tidligere stået i en situation, hvor jeg ikke havde råd til behandling. \|  \| \| 4 \| Mine venner rådede mig til at tegne en sundhedsforsikring. \|  \| \| 5 \| Jeg mener, at det er en del af at være en ansvarlig kæledyrsejer. \|  \| \| 6 \| Jeg har ikke nødvendigvis råd til dyrlægebehandling til mit kæledyr uden forsikring. \|  \| \| 7 \| Fordi mit kæledyr er gammelt og har større risiko for sygdom. \|  \| \| 8 \| Så jeg undgår at skulle trække et svært økonomisk valg, hvis kæledyret bliver syg. \|  \| \| 9 \| Forsikring var inkluderet til en meget billig pris eller var gratis, da jeg købte mit kæledyr (f.eks. i hvalpepakke). \|  \| \| 10 \| Ved ikke \|  \| \| 11 \| Andet \|  \|   **Instruction B.3.2: multiple choice question and only if A3.1sundhedsforsikring=Nej or A.3.2.1sundhedsforsikring=Nej or A.3.2.2sundhedsforsikring=Nej or A.3.3.1sundhedsforsikring=Nej or A3.3.2sundhedsforsikring=Nej or A3.3.3 sundhedsforsikring=Nej or A.3.4.1sundhedsforsikring=Nej or A3.4.2sundhedsforsikring=Nej or A3.4.3sundhedsforsikring=Nej or A.3.6sundhedsforsikring=Nej or A.3.7.1sundhedsforsikring=Nej or A.3.7.2sundhedsforsikring=Nej or A.3.8.1sundhedsforsikring=Nej or A3.8.2sundhedsforsikring=Nej or A3.8.3sundhedsforsikring=Nej or A.3.9.1sundhedsforsikring=Nej or A3.9.2sundhedsforsikring=Nej or A3.9.3sundhedsforsikring=Nej**  **B.3.2 Hvorfor har du ikke tegnet en sundhedsforsikring til dit kæledyr/dine kæledyr? Vælg alle de muligheder, der gælder for dig.**   \| 1 \| Jeg har ikke hørt om det. \|  \| \| --- \| --- \| --- \| \| 2 \| Jeg stoler ikke på forsikringer. \|  \| \| 3 \| Forsikringer til kæledyr er for dyre. \|  \| \| 4 \| Min dyrlæge rådede mig til ikke at tegne en sundhedsforsikring. \|  \| \| 5 \| Fordelene opvejer ikke omkostningerne. \|  \| \| 6 \| Det var for svært at finde en passende forsikring. \|  \| \| 7 \| Kæledyret er for gammelt, så det er ikke længere relevant. \|  \| \| 8 \| Kæledyret er for gammelt, så forsikringen er for dyr. \|  \| \| 9 \| Ved ikke \|  \| \| 10 \| Andet \|  \|   **Instruction B.3.3: multiple choice question and only if A3.1sundhedsforsikring=ikke længere or A.3.2.1sundhedsforsikring=ikke længere or A.3.2.2sundhedsforsikring=ikke længere or A.3.3.1sundhedsforsikring=ikke længere or A3.3.2sundhedsforsikring=ikke længere or A3.3.3 sundhedsforsikring=ikke længere or A.3.4.1sundhedsforsikring=ikke længere or A3.4.2sundhedsforsikring=ikke længere or A3.4.3sundhedsforsikring=ikke længere or A.3.6sundhedsforsikring=ikke længere or A.3.7.1sundhedsforsikring=ikke længere or A.3.7.2sundhedsforsikring=ikke længere or A.3.8.1sundhedsforsikring=ikke længere or A3.8.2sundhedsforsikring=ikke længere or A3.8.3sundhedsforsikring=ikke længere or A.3.9.1sundhedsforsikring=ikke længere or A3.9.2sundhedsforsikring=ikke længere or A3.9.3sundhedsforsikring=ikke længere**  **B.3.3 Hvorfor er du ikke fortsat med at forsikre dit kæledyr/dine kæledyr? Vælg alle de muligheder, der gælder for dig.**   \| 1 \| Fordelene opvejede ikke omkostningerne. \|  \| \| --- \| --- \| --- \| \| 2 \| Det blev for dyrt. \|  \| \| 3 \| Der blev tilføjet for mange undtagelser. \|  \| \| 4 \| Det var ikke alle udgifter, der blev dækket. \|  \| \| 5 \| Ved ikke \|  \| \| 6 \| Andet \|  \| |
| **B.4.: Kunders og dyrlægeklinikkers brug af sociale medier**  **Sociale medier (f.eks. Facebook, Twitter, Instagram) er former for elektronisk kommunikation, hvor brugerne indgår i online fællesskaber for at dele informationer, idéer, personlige beskeder og andet indhold (såsom videoer og billeder).**    **Instruction B.4.1: multiple choice question**  **B.4.1: Hvilke sociale medier bruger du (tænk både på medier, hvor du er tilskuer og dem, hvor du er aktiv medlem)? Vælg alle de muligheder, der gælder for dig.**   \| 1 \| Jeg bruger ikke sociale medier \|  \| \| --- \| --- \| --- \| \| 2 \| Facebook \|  \| \| 3 \| Instagram \|  \| \| 4 \| Twitter \|  \| \| 5 \| YouTube \|  \| \| 6 \| TikTok \|  \| \| 7 \| Andet \|  \|   **Instruction B.4.2: single-choice choice question and only if B4.1=Facebook, B4.1=Instagram, B4.1=Twitter, B4.1=YouTube, B4.1=TikTok and/or B4.1=Andet**  **B.4.2: Hvor ofte bruger du sociale medier?**   \| Hver dag \|  \| \| --- \| --- \| \| 4-6 dage om ugen \|  \| \| 1-3 dage om ugen \|  \| \| Mindre end en gang om ugen \|  \| \| Sjældnere \|  \| \| Ved ikke \|  \|   **Instruction B.4.3: single-choice choice question**  **B.4.3:** **Forventer du, at din dyrlæge er aktiv på de sociale medier?**     \| Ja \|  \| \| --- \| --- \| \| Nej \|  \| \| Ved ikke \|  \|   **B.4.4: Angiv venligst, hvor enig eller uenig du er i de følgende udsagn:**  1= helt uenig; 2 = uenig; 3 = delvist uenig; 4 = neutral (hverken enig eller uenig); 5 = delvist enig; 6 = enig og 7 = helt enig og 8= ved ikke   \|  \| **Jeg tror, at dyrlægens brug af sociale medier ...** \| 1 \| 2 \| 3 \| 4 \| 5 \| 6 \| 7 \| 8 \| \| --- \| --- \| --- \| --- \| --- \| --- \| --- \| --- \| --- \| --- \| \| 1 \| giver folk et indblik i, hvad der sker i klinikken. \|  \|  \|  \|  \|  \|  \|  \|  \| \| 2 \| er unødvendig. \|  \|  \|  \|  \|  \|  \|  \|  \| \| 3 \| er en nyttig måde for nye kæledyrsejere at finde en klinik på. \|  \|  \|  \|  \|  \|  \|  \|  \| \| 4 \| giver kæledyrsejere mulighed for at være i kontakt med dyrlægeklinikken og/eller dyrlægen på en uformel og nem måde. \|  \|  \|  \|  \|  \|  \|  \|  \|   **Instruction B.4.5: single-choice choice question**  **B.4.5:** **Har du nogensinde skrevet en eller flere negative kommentarer om/klager over din dyrlæge på internettet (f.eks. hjemmesider eller sociale medier)?**   \| Ja \|  \| \| --- \| --- \| \| Nej \|  \|   **Instruction B.4.6: multiple choice question and only if B.4.5=yes**  **B.4.6: Hvis ja, hvad handlede den eller de negative kommentarer/klager om? Vælg alle de muligheder, der gælder for dig.**   \| For lang ventetid. \|  \| \| --- \| --- \| \| Dyrlægens råd. \|  \| \| Personalets håndtering af mit kæledyr. \|  \| \| Resultatet af mit kæledyrs behandling/komplikationer. \|  \| \| Prisen for behandlingen. \|  \| \| En bestemt ansats/bestemte ansattes adfærd. \|  \| \| Mangel på teknisk udstyr i klinikken. \|  \| \| Dyrlægen kommunikerer uvenligt. \|  \| \| Dyrlægen kommunikerer for teknisk eller kompliceret. \|  \| \| Personalet kommunikerer uvenligt. \|  \| \| Andet \|  \|   **Instruction B.4.7: single-choice choice question**  **B.4.7:** **Svarede din dyrlæge på den negative feedback/klagen?**   \| Ja \|  \| \| --- \| --- \| \| Nej \|  \| \| Ved ikke \|  \| |
| **B.5: Brug af internettet til at opsøge veterinærmedicinske oplysninger**  **Instruction B.5.1: single-choice choice question**  **B.5.1: Hvor ofte bruger du internettet til at søge faglige oplysninger FØR en dyrlægekonsultation?**   \| Aldrig \|  \| \| --- \| --- \| \| Sommetider \|  \| \| Ofte \|  \| \| Altid \|  \| \| Ved ikke \|  \|   **Instruction B.5.2: single-choice choice question**  **B.5.2: Hvor ofte bruger du internettet til at søge faglige oplysninger EFTER en dyrlægekonsultation?**   \| Aldrig \|  \| \| --- \| --- \| \| Sommetider \|  \| \| Ofte \|  \| \| Altid \|  \| \| Ved ikke \|  \|   **Instruction B.5.3: multiple choice question and only if B.5.1 or B.5.2=sommetider, B.5.1 or B.5.2=ofte, B.5.1 or B.5.2=altid and/or B.5.1 or B.5.2=Ved ikke**  **B.5.3: Hvilke kilder har du brugt?**   \| 1 \| Blogs og chats \|  \| \| --- \| --- \| --- \| \| 2 \| Hjemmesider med dyrlægefaglige oplysninger \|  \| \| 3 \| Sociale medier (f.eks. Facebook, Twitter) \|  \| \| 4 \| Dyrlægens hjemmeside \|  \| \| 5 \| Dyrlægeforeningens hjemmeside \|  \| \| 6 \| Et universitets hjemmeside \|  \| \| 7 \| Andet \|  \|   **Instruction B.5.4: only if B.5.1 or B.5.2=sommetider, B.5.1 or B.5.2=ofte, B.5.1 or B.5.2=altid and/or B.5.1 or B.5.2=ved ikke**  **B.5.4:** **Angiv venligst, hvor enig eller uenig du er i de følgende udsagn:**  1= helt uenig; 2 = uenig; 3 = delvist uenig; 4 = neutral (hverken enig eller uenig); 5 = delvist enig; 6 = enig og 7 = helt enig og 8= ved ikke   \|  \| **Brugen af internetkilder ...** \| 1 \| 2 \| 3 \| 4 \| 5 \| 6 \| 7 \| 8 \| \| --- \| --- \| --- \| --- \| --- \| --- \| --- \| --- \| --- \| --- \| \| 1 \| øger mine forventninger til standarden af den dyrlægebehandling, der er tilgængelig for mit kæledyr. \|  \|  \|  \|  \|  \|  \|  \|  \| \| 2 \| gør mig i stand til at have en mere oplyst drøftelse med min dyrlæge. \|  \|  \|  \|  \|  \|  \|  \|  \| \| 3 \| kan resultere i situationer, hvor jeg er bedre informeret end min dyrlæge. \|  \|  \|  \|  \|  \|  \|  \|  \| \| 4 \| gør mig i stand til at udfordre min dyrlæge til at begrunde hans/hendes anbefalinger. \|  \|  \|  \|  \|  \|  \|  \|  \| \| 5 \| hjælper mig til at tage den rigtige beslutning for mit kæledyr. \|  \|  \|  \|  \|  \|  \|  \|  \| \| 6 \| gør mig i stand til at købe nogle typer medicin billigere (f.eks. loppebehandling, ormekur). \|  \|  \|  \|  \|  \|  \|  \|  \| \| 7 \| kan give et forkert indtryk af dyrlæger (f.eks. hvad de fleste dyrlæger kan tilbyde eller det forventelige udfald af et nyt indgreb). \|  \|  \|  \|  \|  \|  \|  \|  \|   **Instruction B.5.5: single-choice question and only if B.5.1 or B.5.2=sommetider, B.5.1 or B.5.2=ofte, B.5.1 or B.5.2=altid and/or B.5.1 or B.5.2=ved ikke**  **B.5.5: Har du nogensinde været uenig i din dyrlæges professionelle råd baseret på oplysninger, som du havde fundet på internettet?**   \| Ja \|  \| \| --- \| --- \| \| Nej \|  \| \| Ved ikke \|  \| |
| **B.6: Telemedicin i praksis med smådyr**  **Telemedicin er en ny mulighed inden for dyrlægepraksis, hvor der ved hjælp af elektroniske medier gives råd og behandling ”fra distancen”. Den gør kæledyrsejere i stand til at have en hel konsultation med deres dyrlæge – som man skal betale for - uden at tage deres dyr med hen til klinikken.**  **Instruction B.6.1: single-choice question**  **B.6.1: Har du nogensinde brugt telemedicin til at få faglig rådgivning om dit/dine kæledyr?**   \| Ja \|  \| \| --- \| --- \| \| Nej \|  \| \| Jeg vidste ikke, at det var en mulighed \|  \|     **Instruction B.6.2: single-choice question and only if B.6.1=Nej or B.6.1=** **Jeg vidste ikke, at det var en mulighed**  **B.6.2: Ville du gøre brug af telemedicin, hvis din dyrlæge tilbød det i stedet for en fysisk konsultation?**   \| Ja \|  \| \| --- \| --- \| \| Nej \|  \| \| Ved ikke \|  \|   **B.6.3: Angiv venligst, hvor enig eller uenig du er i de følgende udsagn:**  1= helt uenig; 2 = uenig; 3 = delvist uenig; 4 = neutral (hverken enig eller uenig); 5 = delvist enig; 6 = enig og 7 = helt enig og 8= ved ikke   \|  \| **Brugen af telemedicin ...** \| 1 \| 2 \| 3 \| 4 \| 5 \| 6 \| 7 \| 8 \| \| --- \| --- \| --- \| --- \| --- \| --- \| --- \| --- \| --- \| --- \| \| 1 \| kan være nyttig, da jeg synes, at transporten hen til dyrlægen er besværlig. \|  \|  \|  \|  \|  \|  \|  \|  \| \| 2 \| kan spare mit kæledyr for en stressende transport hen til dyrlægen. \|  \|  \|  \|  \|  \|  \|  \|  \| \| 3 \| kan hjælpe til at beslutte, om mit kæledyr har behov for at blive tilset af en dyrlæge. \|  \|  \|  \|  \|  \|  \|  \|  \| \| 4 \| kan være nyttig ved opfølgningskonsultationer. \|  \|  \|  \|  \|  \|  \|  \|  \| \| 5 \| kan gøre tilgangen til en specialist nemmere, hvis der ikke er én i nærheden. \|  \|  \|  \|  \|  \|  \|  \|  \| \| 6 \| er god i nødstilfælde. \|  \|  \|  \|  \|  \|  \|  \|  \| \| 7 \| svækker båndet mellem dyrlæge og klient. \|  \|  \|  \|  \|  \|  \|  \|  \| \| 8 \| styrker båndet mellem dyrlæge og klient. \|  \|  \|  \|  \|  \|  \|  \|  \| \| 9 \| bør koste mindre end en normal konsultation. \|  \|  \|  \|  \|  \|  \|  \|  \| \| 10 \| har ingen fordele. \|  \|  \|  \|  \|  \|  \|  \|  \| \| 11 \| øger risikoen for, at noget bliver overset, fordi dyret ikke undersøges fysisk af dyrlægen. \|  \|  \|  \|  \|  \|  \|  \|  \| \| 12 \| er mere bekvemt end at møde op fysisk. \|  \|  \|  \|  \|  \|  \|  \|  \| \| 13 \| er ikke en mulighed for mig, da jeg ikke har de nødvendige IT-færdigheder eller IT-udstyr. \|  \|  \|  \|  \|  \|  \|  \|  \| |
|  |
| **Tak fordi du deltog i denne undersøgelse!** |

| **Introduction and background:**  **Thank you for participating in this survey.**  The **purpose** of this survey is to **explore why some people keep pets and others do not, and to explore the attitudes of pet owners to developments in modern small animal practice.**  The **survey will take approximately 3 minutes** to complete for **non-pet owners**. **If you have a pet**, we will also ask about **your attachment to your pet** (or pets), and your **attitude towards modern veterinary practice** and **expectations of veterinary services**. There are also some questions about health insurance for pets, as well as the use of social media and internet resources in relation to veterinary treatment. For **pet owners, the questionnaire will take approximately 15- 20 minutes** to complete.    This questionnaire is from a joint research project with three involved countries: Denmark, Austria, and the UK. The respective researchers are from the University of Copenhagen, University of Veterinary Medicine, Vienna, and the University of Glasgow.  **Completion** of the questionnaire is **voluntary,** and you can **exit at any point prior to submitting the final answer**. Your responses will then be passed to the researchers in an **anonymized** form, and **no information can be traced back to you**.  When clicking on the button “Next”, you confirm that you are over 17 years old, and consent to participate in this survey.  **Thank you for your contribution!** |
| --- |
| **SECTION A:**  **Information on your pet, demographics, and the veterinary practice/clinic you attend.**  **Instruction A.1: single-choice question**  **A.1: Pets are often kept at home, but we also want to hear about animals that are not kept at home (e.g. horses). However, we are not interested here in production animals kept on farms e.g. dairy cows.**  **Do you have one or more pet(s)?**   \| Yes \|  \| \| --- \| --- \| \| No \|  \|   **Instruction A.2: multiple choice question and only if A1=Yes**  **A.2: How many of the following pets do you have? Please indicate the number of each type, or select 0 (none) where appropriate.**   \|  \| 0 (none) \| 1 \| 2 \| 3 \| 4 \| More than 4 \| \| --- \| --- \| --- \| --- \| --- \| --- \| --- \| \| Dog \|  \|  \|  \|  \|  \|  \| \| Cat \|  \|  \|  \|  \|  \|  \| \| Horse \|  \|  \|  \|  \|  \|  \| \| Rabbit \|  \|  \|  \|  \|  \|  \| \| Rodent (e.g. hamster, guinea pig, chinchilla, mouse/rat) \|  \|  \|  \|  \|  \|  \| \| Bird \|  \|  \|  \|  \|  \|  \| \| Reptile (e.g. lizard, snake, turtle) \|  \|  \|  \|  \|  \|  \| \| Fish (both aquarium fish and fish in garden pond) \|  \|  \|  \|  \|  \|  \| \| Other pet than the above mentioned \|  \|  \|  \|  \|  \|  \|   *Construct filter variable =* ***Number of pet species***  *Count =* ***Number of pet species*** *if A2Dog>0, A2Cat>0, A2Horse>0, A2Rabbit>0, A2Rodent>0, A2Bird>0, A2Reptile>0, A2Fish>0, A2*Other pet than the above mentioned *>0*  **Instruction A.3.1: only if A2Dog=1**  **A.3.1: Please provide the age of your dog, the number of visits to a veterinarian in the last 12 months, and whether the dog is covered by a health insurance.**  Scroll down menu: age (<0.5 - >20 years)  Scroll down menu: number of visits to a veterinarian in the last 12 months (0 - > 20)  Scroll down menu: health insurance (yes, no, not anymore)  **Instruction A.3.1.1: English questionnaire: single-choice question and only if A3.1Health insurance=Yes**  **A.3.1.1: What type of insurance do you have for your dog?**  scroll-down menu (A policy where the limit is *per year,* A policy where the limit is *per condition, I don’t know)*  **Instruction A.3.1.2: single-choice question and only if A.3.1.1=A policy where the limit is *per year*.**  **A.3.1.2: What is the insurance limit for your dog?**  scroll-down menu (I don’t know, up to £1500 per year, between £1501- £4000 per year, between £4001- £8000 per year, more than £8000 per year)  **Instruction A.3.1.3: single-choice question and only if A.3.1.1=A policy where the limit is *per condition*.**  **A.3.1.3: What is the insurance limit for your dog?**  scroll-down menu (I don’t know, up to £1500 per condition, between £1501- £4000 per condition, between £4001- £8000 per condition, more than £8000 per condition)  **Instruction A.3.2: only if A2Dog=2**  **A.3.2: Please provide the age of your dogs, the number of visits to a veterinarian in the last 12 months, and whether the two dogs are covered by health insurance.**  **A.3.2.1:**  Scroll down menu: age (<0.5 - >20 years)  Scroll down menu: number of visits to a veterinarian in the last 12 months (0 - > 20)  Scroll down menu: health insurance (yes, no, not anymore)  **A.3.2.2:**  Scroll down menu: age (<0.5 - >20 years)  Scroll down menu: number of visits to a veterinarian in the last 12 months (0 - > 20)  Scroll down menu: health insurance (yes, no, not anymore)  **Instruction A.3.3: only if A2Dog=3**  **A.3.3: Please provide the age of your dogs, the number of visits to a veterinarian in the last 12 months, and whether the three dogs are covered by health insurance.**  **A.3.3.1:**  Scroll down menu: age (<0.5 - >20 years)  Scroll down menu: number of visits to a veterinarian in the last 12 months (0 - > 20)  Scroll down menu: health insurance (yes, no, not anymore)  **A.3.3.2:**  Scroll down menu: age (<0.5 - >20 years)  Scroll down menu: number of visits to a veterinarian in the last 12 months (0 - > 20)  Scroll down menu: health insurance (yes, no, not anymore)    **A.3.3.3:**  Scroll down menu: age (<0.5 - >20 years)  Scroll down menu: number of visits to a veterinarian in the last 12 months (0 - > 20)  Scroll down menu: health insurance (yes, no, not anymore)  **Instruction A.3.4: only if A2Dog>3**  **A.3.4: We will now ask about the age of three of your dogs, the number of visits to a veterinarian in the last 12 months, and whether the three dogs are covered by health insurance. Please choose the three dogs whose names come first in the alphabet.**  **A.3.4.1:** First dog (first letter in name comes first in the alphabet)  Scroll down menu: age (<0.5 - >20 years)  Scroll down menu: number of visits to a veterinarian in the last 12 months (0 - > 20)  Scroll down menu: health insurance (yes, no, not anymore)  **A.3.4.2:** Second dog (first letter in name comes second in the alphabet)  Scroll down menu: age (<0.5 - >20 years)  Scroll down menu: number of visits to a veterinarian in the last 12 months (0 - > 20)  Scroll down menu: health insurance (yes, no, not anymore)  **A.3.4.3:** Third dog (first letter in name comes third in the alphabet)  Scroll down menu: age (<0.5 - >20 years)  Scroll down menu: number of visits to a veterinarian in the last 12 months (0 - > 20)  Scroll down menu: health insurance (yes, no, not anymore)  **Instruction A.3.5: English questionnaire: only if A.3.2.1Health insurance=Yes or A.3.2.2Health insurance=Yes or A.3.3.1Health insurance=Yes or A3.3.2Health insurance=Yes or A3.3.3Health insurance=Yes or A.3.4.1Health insurance=Yes or A3.4.2Health insurance=Yes or A3.4.3Health insurance=Yes**  **A.3.5: What type of insurance do you have for your dog(s)? *If the type of insurance is not the same for all dogs, then think about the dog whose name comes first in the alphabet.***  Scroll-down menu (A policy where the limit is *per year,* A policy where the limit is *per condition, I don’t know)*  **Instruction A.3.5.1: single-choice question and only if A.3.5 =A policy where the limit is *per year*.**  **A.3.5.1: What is the insurance limit for your dog(s)? *If the limit is not the same for all dogs, then think about the dog whose name comes first in the alphabet.***  Scroll-down menu (I don’t know, up to £1500 per year, between £1501- £4000 per year, between £4001- £8000 per year, more than £8000 per year)  **Instruction A.3.5.2: single-choice question and only if A.3.5 =A policy where the limit is *per condition*.**  **A.3.5.2: What is the insurance limit for your dog(s)? *If the limit is not the same for all dogs, then think about the dog whose name comes first in the alphabet.***  Scroll-down menu (I don’t know, up to £1500 per condition, between £1501- £4000 per condition, between £4001- £8000 per condition, more than £8000 per condition)  **Instruction A.3.6: only if A2Cat=1**  **A.3.6: Please provide the age of your cat, the number of visits to a veterinarian in the last 12 months, and whether the cat is covered by health insurance.**  Scroll down menu: age (<0.5 - >20 years)  Scroll down menu: number of visits to a veterinarian in the last 12 months (0 - > 20)  Scroll down menu: health insurance (yes, no, not anymore)  **Instruction A.3.6.1: English questionnaire: single-choice question and only if A3.6Health insurance=Yes**  **A.3.6.1: What type of insurance do you have for your cat?**  scroll-down menu (A policy where the limit is *per year,* A policy where the limit is *per condition, I don’t know)*  **Instruction A.3.6.2: single-choice question and only if A.3.6.1 =A policy where the limit is *per year*.**  **A.3.6.2: What is the insurance limit for your cat?**  Scroll-down menu (I don’t know, up to £1500 per year, between £1501- £4000 per year, between £4001- £8000 per year, more than £8000 per year)  **Instruction A.3.6.3: single-choice question and only if A.3.6.1 =A policy where the limit is *per condition*.**  **A.3.6.3: What is the insurance limit for your cat?**  Scroll-down menu (I don’t know, up to £1500 per condition, between £1501- £4000 per condition, between £4001- £8000 per condition, more than £8000 per condition)  **Instruction A.3.7: only if A2Cat=2**  **A.3.7: Please provide the age of your cats, the number of visits to a veterinarian in the last 12 months, and whether the two cats are covered by health insurance.**  **A.3.7.1:**  Scroll down menu: age (<0.5 - >20 years)  Scroll down menu: number of visits to a veterinarian in the last 12 months (0 - > 20)  Scroll down menu: health insurance (yes, no, not anymore)  **A.3.7.2:**  Scroll down menu: age (<0.5 - >20 years)  Scroll down menu: number of visits to a veterinarian in the last 12 months (0 - > 20)  Scroll down menu: health insurance (yes, no, not anymore)  **Instruction A3.8: only if A2Cat=3**  **A.3.8: Please provide the age of your cats, the number of visits to a veterinarian in the last 12 months, and whether the three cats are covered by health insurance.**  **A.3.8.1:**  Scroll down menu: age (<0.5 - >20 years)  Scroll down menu: number of visits to a veterinarian in the last 12 months (0 - > 20)  Scroll down menu: health insurance (yes, no, not anymore)  **A.3.8.2:**  Scroll down menu: age (<0.5 - >20 years)  Scroll down menu: number of visits to a veterinarian in the last 12 months (0 - > 20)  Scroll down menu: health insurance (yes, no, not anymore)  **A.3.8.3:**  Scroll down menu: age (<0.5 - >20 years)  Scroll down menu: number of visits to a veterinarian in the last 12 months (0 - > 20)  Scroll down menu: health insurance (yes, no, not anymore)  **Instruction A.3.9: only if A2Cat>3**  **A.3.9: We will now ask about the age of three of your cats, the number of visits to a veterinarian in the last 12 months, and whether the three cats are covered by health insurance. Please choose the three cats whose names come first in the alphabet.**  **A.3.9.1:** First cat (first letter in name comes first in the alphabet)  Scroll down menu: age (<0.5 - >20 years)  Scroll down menu: number of visits to a veterinarian in the last 12 months (0 - > 20)  Scroll down menu: health insurance (yes, no, not anymore)  **A.3.9.2:** Second cat (first letter in name comes second in the alphabet)  Scroll down menu: age (<0.5 - >20 years)  Scroll down menu: number of visits to a veterinarian in the last 12 months (0 - > 20)  Scroll down menu: health insurance (yes, no, not anymore)  **A.3.9.3:** Third cat (first letter in name comes third in the alphabet)  Scroll down menu: age (<0.5 - >20 years)  Scroll down menu: number of visits to a veterinarian in the last 12 months (0 - > 20)  Scroll down menu: health insurance (yes, no, not anymore)  **Instruction A.3.10: English questionnaire: only if A.3.7.1Health insurance=Yes or A.3.7.2Health insurance=Yes or A.3.8.1Health insurance=Yes or A3.8.2Health insurance=Yes or A3.8.3Health insurance=Yes or A.3.9.1Health insurance=Yes or A3.9.2Health insurance=Yes or A3.9.3Health insurance=Yes**  **A.3.10: What type of insurance do you have for your cat(s)? *If the type of insurance is not the same for all cats, then think about the cat whose name comes first in the alphabet.***  Scroll-down menu (A policy where the limit is *per year,* A policy where the limit is *per condition, I don’t know)*  **Instruction A.3.10.1: single-choice question and only if A.3.10=A policy where the limit is *per year*.**  **A.3.9.1: What is the insurance limit for your cat(s)? *If the limit is not the same for all cats, then think about the cat whose name comes first in the alphabet.***  Scroll-down menu (I don’t know, up to £1500 per year, between £1501- £4000 per year, between £4001- £8000 per year, more than £8000 per year)  **Instruction A.3.10.2: single-choice question and only if A.3.10=A policy where the limit is *per condition*.**  **A.3.10.2: What is the insurance limit for your cat(s)? *If the limit is not the same for all cats, then think about the cat whose name comes first in the alphabet.***  Scroll-down menu (I don’t know, up to £1500 per condition, between £1501- £4000 per condition, between £4001- £8000 per condition, more than £8000 per condition)  **Instruction A.4: multiple choice question and only if A1=No**  **A.4: Why you do not have a dog and/or a cat?**   \| I do not like dogs and/or cats. \|  \| \| --- \| --- \| \| Someone I live with does not like dogs and/or cats. \|  \| \| I do not have time to care for a dog and/or cat. \|  \| \| I cannot afford to care for a dog and/or cat. \|  \| \| I / someone in my household have/ has allergies that would be affected. \|  \| \| I am not allowed to keep dogs and/or cats in my accommodation. \|  \| \| I live in a flat and I do not think it is fair to keep a dog or a cat in that setting. \|  \| \| I believe that people should not keep pets at all because it violates animals’ rights. \|  \| \| Other reasons \|  \|   **Instruction A.4.1: open text field and only if A4=”Other reasons”**  **You are welcome to explain why you don’t have a dog or cat.**   \| **Open response:** \| \| \| --- \| --- \| \| **I have no more to add.** \|  \|   **Instruction A.5: drop-down menu from 18 - 100 years + “prefer not to say”**  **A.5:** **Please enter your current age in years from the drop-down menu.**  ________ years old.  **Instruction A.6: single-choice question**  **A.6: Please indicate your gender.**   \| Male \|  \| \| --- \| --- \| \| Female \|  \| \| Neither of these \|  \| \| Prefer not to say \|  \|   **Instruction A.7: single-choice question**  **A.7: In which area is your primary place of residence?**  **For UK:**   \| Scotland \|  \| \| --- \| --- \| \| Northern Ireland \|  \| \| Wales \|  \| \| England - North East \|  \| \| England - North West \|  \| \| England - Yorkshire and Humberside \|  \| \| England - West Midlands \|  \| \| England - East Midlands \|  \| \| England - South West \|  \| \| England - South East \|  \| \| England - Greater London \|  \|   **Instruction A.8: single-choice question**  **A.8: Do you / did you work in the veterinary field (e.g. practicing veterinarian, veterinary nurse, veterinary assistant)?**   \| Yes \|  \| \| --- \| --- \| \| No \|  \|   **Instruction A.10.1: single-choice question**  **A.10.1: Do you live alone (not including any pets)?**   \| Yes \|  \| \| --- \| --- \| \| No \|  \|   **Instruction A.10.2: single-choice question and only if A10.1=No and list in drop-down-menu**  **A.10.2: Who do you live with?**   \| I live with one or more adult(s) \|  \| \| --- \| --- \| \| I live with one or more adult(s) and one child below 18 years (full time) \|  \| \| I live with one or more adult(s) and one child below 18 years (part-time) \|  \| \| I live with one or more adult(s) and children below 18 years (full time) \|  \| \| I live with one or more adult(s) and children below 18 years (part-time) \|  \| \| I live with one child below 18 years (full time) \|  \| \| I live with one child below 18 years (part time) \|  \| \| I live with children below 18 years (full time) \|  \| \| I live with children below 18 years (part time) \|  \| \| Other \|  \|   **Instruction A11.1: single-choice question and only if A2=Dog & A10.2 = “I live with one or more adult(s)” or “I live with one or more adult(s) and one child below 18 years (full time)” or “I live with one or more adult(s) and one child below 18 years (part-time)” or “I live with one or more adult(s) and children below 18 years (full time)” or “I live with one or more adult(s) and children below 18 years (part-time)” or “I live with one child below 18 years (full time)” or “I live with one child below 18 years (part time)” or “I live with children below 18 years (full time)” or “I live with children below 18 years (part time)”**  **A.11.1: For whom was/were the dog(s) acquired?**   \| All (or most) in the household \|  \| \| --- \| --- \| \| Me \|  \| \| My partner/spouse \|  \| \| My child / step child \|  \| \| My children / step children \|  \| \| For (an)other pet(s) in the household (as a companion) \|  \| \| Others \|  \| \| I don’t know \|  \|   **Instruction A11.2: single-choice question and only if A2=Cat & A10.2 = “I live with one or more adult(s)” or “I live with one or more adult(s) and one child below 18 years (full time)” or “I live with one or more adult(s) and one child below 18 years (part-time)” or “I live with one or more adult(s) and children below 18 years (full time)” or “I live with one or more adult(s) and children below 18 years (part-time)” or “I live with one child below 18 years (full time)” or “I live with one child below 18 years (part time)” or “I live with children below 18 years (full time)” or “I live with children below 18 years (part time)”**  **A.11.2: For whom was/were the cat(s) acquired?**   \| All (or most) in the household \|  \| \| --- \| --- \| \| Me \|  \| \| My partner/spouse \|  \| \| My child / step child \|  \| \| My children / step children \|  \| \| For (an)other pet(s) in the household (as a companion) \|  \| \| Others \|  \| \| I don’t know \|  \|   **Instruction A12.1: single-choice question and only if A2=Dog & A10.2 = “I live with one or more adult(s)” or “I live with one or more adult(s) and one child below 18 years (full time)” or “I live with one or more adult(s) and one child below 18 years (part-time)” or “I live with one or more adult(s) and children below 18 years (full time)” or “I live with one or more adult(s) and children below 18 years (part-time)” or “I live with one child below 18 years (full time)” or “I live with one child below 18 years (part time)” or “I live with children below 18 years (full time)” or “I live with children below 18 years (part time)”**  **A.12.1: Who in the household do you think is most attached to the dog(s)?**   \| All (or most) in the household \|  \| \| --- \| --- \| \| Me \|  \| \| My partner/spouse \|  \| \| My child / step child \|  \| \| My children / step children \|  \| \| Others \|  \| \| I don’t know \|  \|   **Instruction A12.2: single-choice question and only if A2=Cat & A10.2 = “I live with one or more adult(s)” or “I live with one or more adult(s) and one child below 18 years (full time)” or “I live with one or more adult(s) and one child below 18 years (part-time)” or “I live with one or more adult(s) and children below 18 years (full time)” or “I live with one or more adult(s) and children below 18 years (part-time)” or “I live with one child below 18 years (full time)” or “I live with one child below 18 years (part time)” or “I live with children below 18 years (full time)” or “I live with children below 18 years (part time)”**  **A.12.2: Who in the household do you think is most attached to the cat(s)?**   \| All (or most) in the household \|  \| \| --- \| --- \| \| Me \|  \| \| My partner/spouse \|  \| \| My child / step child \|  \| \| My children / step children \|  \| \| Others \|  \| \| I don’t know \|  \|   **Instruction A13.1: multiple choice question and only if A2=Dog**  **A.13.1: Why was/were the dog(s) acquired? Choose all that are relevant for you.**   \| To provide companionship (for humans) \|  \| \| --- \| --- \| \| As company for other pet(s) \|  \| \| To breed from \|  \| \| To show in competitions / exhibitions \|  \| \| For dog sports (training, agility, etc.) \|  \| \| To exercise with (walks) \|  \| \| As a hunting dog \|  \| \| To protect home and/or property \|  \| \| Other \|  \| \| I don’t know \|  \|   **Instruction A13.2: multiple choice question and only if A2=Cat**  **A.13.2: Why was/were the cat(s) acquired? Choose all that are relevant for you.**   \| To provide companionship (for humans) \|  \| \| --- \| --- \| \| As company for other pet(s) \|  \| \| To breed from \|  \| \| To show in competitions / exhibitions \|  \| \| To keep rodents away from the home \|  \| \| It/they just appeared at my home and stayed \|  \| \| Other \|  \| \| I don’t know \|  \|   **Instruction 14.1: only if**  **Number of pet species=1 & A2Dog=1**  **Number of pet species=1 & A2Cat=1**  **Number of pet species=1 & A2Horse=1**  **Number of pet species=1 & A2Rabbit=1**  **Number of pet species=1 & A2Rodent=1**  **Number of pet species=1 & A2Bird=1**  **Number of pet species=1 & A2Reptile=1**  **Number of pet species=1 & A2Fish=1**  **Number of pet species=1 & A2Other pet than the above mentioned** **=1**  **A14.1: Please think about your pet and indicate whether you strongly disagree, somewhat disagree, somewhat agree, or strongly agree in the following statements.**  *Please use answer options /matrix provided under question A.14.11.1*  **Instruction 14.2: only if Number of pet species=1 & A2Dog>1**  **A14.2: Please think about your favourite dog and indicate whether you strongly disagree, somewhat disagree, somewhat agree, or strongly agree in the following statements.**  *Please use answer options /matrix provided under question A.14.11.1*  **Instruction 14.3: only if Number of pet species=1 & A2Cat>1**  **A14.3: Please think about your favourite cat and indicate whether you strongly disagree, somewhat disagree, somewhat agree, or strongly agree in the following statements.**  *Please use answer options /matrix provided under question A.14.11.1*  **Instruction 14.4: only if Number of pet species=1 & A2Horse>1**  **A14.4: Please think about your favourite horse and indicate whether you strongly disagree, somewhat disagree, somewhat agree, or strongly agree in the following statements.**  *Please use answer options /matrix provided under question A.14.11.1*  **Instruction 14.5: only if Number of pet species=1 & A2Rabbit>1**  **A14.5: Please think about your favourite rabbit and indicate whether you strongly disagree, somewhat disagree, somewhat agree, or strongly agree in the following statements.**  *Please use answer options /matrix provided under question A.14.11.1*  **Instruction 14.6: only if Number of pet species=1 & A2Rodent>1**  **A14.6: Please think about your favourite rodent and indicate whether you strongly disagree, somewhat disagree, somewhat agree, or strongly agree in the following statements.**  *Please use answer options /matrix provided under question A.14.11.1*  **Instruction 14.7: only if Number of pet species=1 & A2Bird>1**  **A14.7: Please think about your favourite bird and indicate whether you strongly disagree, somewhat disagree, somewhat agree, or strongly agree in the following statements.**  *Please use answer options /matrix provided under question A.14.11.1*  **Instruction 14.8: only if Number of pet species=1 & A2Reptile>1**  **A14.8: Please think about your favourite reptile and indicate whether you strongly disagree, somewhat disagree, somewhat agree, or strongly agree e in the following statements.**  *Please use answer options /matrix provided under question A.14.11.1*  **Instruction 14.9: only if Number of pet species=1 & A2Fish>1**  **A14.9: Please think about your favourite fish and indicate whether you strongly disagree, somewhat disagree, somewhat agree, or strongly agree in the following statements.**  *Please use answer options /matrix provided under question A.14.11.1*  **Instruction 14.10: only if Number of pet species=1 & A2Other pet than the above mentioned** **>1**  **A14.10: Please think about your favourite pet and indicate whether you strongly disagree, somewhat disagree, somewhat agree, or strongly agree in the following statements.**  *Please use answer options /matrix provided under question A.14.11.1*  **Instruction A.14.11: only if Number of pet species>1**  **A14.11: Now we would like you to think about your favourite pet. What species is your favourite pet?**   \| Dog \|  \| \| --- \| --- \| \| Cat \|  \| \| Horse \|  \| \| Rabbit \|  \| \| Rodent (e.g. hamster, guinea pig, chinchilla, mouse/rat) \|  \| \| Bird \|  \| \| Reptile (e.g. lizard, snake, turtle) \|  \| \| Fish (both aquarium fish and fish in garden pond) \|  \| \| Other pet than the above mentioned \|  \|   **Instruction 14.11.1: only if Number of pet species>1**  **A14.11.1: Now please indicate whether you strongly disagree, somewhat disagree, somewhat agree, or strongly agree to the following statements about your favourite pet.**  *1=strongly disagree, 2=somewhat disagree, 3=somewhat agree and 4=strongly agree*   \|  \| **Attachment to my pet** \| **1** \| **2** \| **3** \| **4** \| \| --- \| --- \| --- \| --- \| --- \| --- \| \| 1 \| My pet means more to me than any of my friends \|  \|  \|  \|  \| \| 2 \| Quite often I confide in my pet \|  \|  \|  \|  \| \| 3 \| I believe that pets should have the same rights and privileges as family members \|  \|  \|  \|  \| \| 4 \| I believe my pet is my best friend \|  \|  \|  \|  \| \| 5 \| Quite often, my feelings towards people are affected by how they react to my pet \|  \|  \|  \|  \| \| 6 \| I love my pet because he/she is more loyal to me than most of the people in my life \|  \|  \|  \|  \| \| 7 \| I enjoy showing other people pictures of my pet \|  \|  \|  \|  \| \| 8 \| I think my pet is just a pet \|  \|  \|  \|  \| \| 9 \| I love my pet because it never judges me \|  \|  \|  \|  \| \| 10 \| My pet knows when I’m feeling bad \|  \|  \|  \|  \| \| 11 \| I often talk to other people about my pet \|  \|  \|  \|  \| \| 12 \| My pet understands me \|  \|  \|  \|  \| \| 13 \| I believe that loving my pet helps me stay heathy \|  \|  \|  \|  \| \| 14 \| Pets deserve as much respect as humans do \|  \|  \|  \|  \| \| 15 \| My pet and I have a very close relationship \|  \|  \|  \|  \| \| 16 \| I would do almost anything to take care of my pet \|  \|  \|  \|  \| \| 17 \| I play with my pet quite often \|  \|  \|  \|  \| \| 18 \| I consider my pet to be great companion \|  \|  \|  \|  \| \| 19 \| My pet makes me feel happy \|  \|  \|  \|  \| \| 20 \| I feel that my pet is part of my family \|  \|  \|  \|  \| \| 21 \| I am not very attached to my pet \|  \|  \|  \|  \| \| 22 \| Owning a pet adds to my happiness \|  \|  \|  \|  \| \| 23 \| I consider my pet to be a friend \|  \|  \|  \|  \|   **Instruction A15.1: only if A2=Dog**  **A.15.1: Please indicate which type of veterinary practice you usually attend with your dog(s):**  **Instruction A15.2: only if A2=Cat**  **A.15.2: Please indicate which type of veterinary practice you usually attend with your cat(s):**  **For UK:**   \| Small animal practice (1-3 veterinarians) \|  \| \| --- \| --- \| \| Small animal practice (4 or more veterinarians) \|  \| \| Mixed practice (where other types of animals are also treated e.g. farm animals) \|  \| \| University hospital \|  \| \| Charity clinic \|  \| \| I attend several types of practice. \|  \| \| I do not have a veterinary practice that I attend \|  \| \| I don't know \|  \|   **Instruction A.16: only if A2=Dog &/or A2=Cat**  **A.16: How important are the following aspects to you when thinking about your veterinarian?**  1= strongly disagree; 2 = disagree; 3 = somewhat disagree; 4 = neutral (neither agree nor disagree); 5 = somewhat agree; 6 = agree and 7 = strongly agree; 8=I don’t know   \|  \| **It is important that…** \| 1 \| 2 \| 3 \| 4 \| 5 \| 6 \| 7 \| 8 \| \| --- \| --- \| --- \| --- \| --- \| --- \| --- \| --- \| --- \| --- \| \| 1 \| I see the same veterinarian every time. \|  \|  \|  \|  \|  \|  \|  \|  \| \| 2 \| I have a good relationship with / like my veterinarian. \|  \|  \|  \|  \|  \|  \|  \|  \| \| 3 \| my animal likes /seems comfortable with my veterinarian. \|  \|  \|  \|  \|  \|  \|  \|  \| \| 4 \| my veterinarian seems to care about my pet \|  \|  \|  \|  \|  \|  \|  \|  \| \| 5 \| my veterinarian has specialist qualifications in addition to their veterinary degree \|  \|  \|  \|  \|  \|  \|  \|  \| \| 6 \| my vet has a professional manner \|  \|  \|  \|  \|  \|  \|  \|  \| \| 7 \| I can trust my veterinarian \|  \|  \|  \|  \|  \|  \|  \|  \|   **Instruction A.17.: only if A2=Dog &/or A2=Cat**  **A.17: How important are the following aspects to you when choosing a practice?**  *1= “Not important at all”; 2=”less important”; 3=” important”; 4=” really important”; 5=” I don’t know”*   \|  \| **The practice should…** \| **1** \| **2** \| **3** \| **4** \| **5** \| \| --- \| --- \| --- \| --- \| --- \| --- \| --- \| \| 1 \| - be near where I live \|  \|  \|  \|  \|  \| \| 2 \| - provide 24 hour care for routine emergencies themselves (not divert me elsewhere) \|  \|  \|  \|  \|  \| \| 3 \| - be accessible with good parking \|  \|  \|  \|  \|  \| \| 4 \| - have appointments available at convenient times \|  \|  \|  \|  \|  \| \| 5 \| - consider species-related requirements (e.g. separate waiting room for cats to reduce stress) \|  \|  \|  \|  \|  \| \| 6 \| - offer specialist care if required e.g. joint replacements or cancer treatments \|  \|  \|  \|  \|  \| \| 7 \| - offer alternative medicine e.g. traditional Chinese medicine, homeopathy \|  \|  \|  \|  \|  \| \| 8 \| - charge reasonable prices \|  \|  \|  \|  \|  \| \| 9 \| - be independently-owned \|  \|  \|  \|  \|  \| \| 10 \| - be corporate-owned e.g. AniCura, IVC *(only UK IVC)* \|  \|  \|  \|  \|  \| \| 11 \| - make house calls \|  \|  \|  \|  \|  \| \| 12 \| - offer payment plans \|  \|  \|  \|  \|  \| \| 13 \| - make direct insurance claims \|  \|  \|  \|  \|  \|   **Instruction A.18: multiple-choice question and only if A2=Dog &/or A2=Cat**  **A.18: Which of the following diagnostic and treatment options would you expect to be available in the practice you usually attend? Please tick all that apply.**   \| **Radiography** (x-rays) \|  \| \| --- \| --- \| \| **Ultrasound** (an imaging technique commonly used for examining the abdomen) \|  \| \| **Endoscopy** (camera to look inside body, remove foreign bodies from passageways e.g stomach, airway - enables ‘keyhole’ surgery). \|  \| \| **Arthroscopy** (camera to look inside joints) \|  \| \| **In-house laboratory** (e.g. for blood tests, urine analysis) \|  \| \| **Dental equipment** \|  \| \| **MRI** scanner (an advanced imaging technique commonly used for the spine) \|  \| \| **CT scanner** (an advanced an advanced imaging technique commonly used for the chest) \|  \| \| **None of the above** \|  \| \| **I don’t know** \|  \|   **Instruction A.19.1: single-choice question and only if A2=Dog**  **A.19.1: Have you ever taken your dog(s) to a specialist veterinarian (e.g. neurologist, behaviourist, oncologist)?**   \| Yes \|  \| \| --- \| --- \| \| No \|  \| \| I don’t know \|  \|   **Instruction A.19.2: single-choice question and only if A2=Cat**  **A.19.2: Have you ever taken your cat(s) to a specialist veterinarian (e.g. neurologist, behaviourist, oncologist)?**   \| Yes \|  \| \| --- \| --- \| \| No \|  \| \| I don’t know \|  \|   **Instruction A.20.1: single-choice question and only if A2=Dog & A.3.1-A.3.4Health Insurance=No or A.3.1-A3.4 Health Insurance=Not anymore**  **A.20.1: If (one of) your uninsured dog(s) was suffering from a severe illness, and would have to either undergo treatment (with a good chance of a successful outcome), or be euthanised, what would you do?**  **Instruction A.20.2: single-choice question and only if A2=Cat & A.3.6-A.3.9Health Insurance=No or A.3.6-A3.9Health Insurance=Not anymore**  **A.20.2: If (one of) your uninsured cat(s) was suffering from a severe illness, and would have to either undergo treatment (with a good chance of a successful outcome), or be euthanised, what would you do?**  **UK:**   \| **I would ask for euthanasia.** \|  \| \| --- \| --- \| \| **I would spend < £100 on treatment.** \|  \| \| **I would spend £101-500 on treatment.** \|  \| \| **I would spend £501-1000 on treatment.** \|  \| \| **I would spend £1001-3000 on treatment.** \|  \| \| **I would spend £3001-5000 on treatment.** \|  \| \| **I would spend £5001-7999 on treatment.** \|  \| \| **I would spend £8000 or more on treatment.** \|  \| \| **I don’t know** \|  \|   **Instruction A.21.1: single-choice question and only if A2=Dog & A.3.1-A.3.4Health Insurance=Yes**  **A.21.1: If (one of) your insured dog(s) were suffering from a severe illness, and would have to either undergo treatment (with a good chance of a successful outcome), or be euthanised, what would you do?**  **Instruction A.21.2: single-choice question and only if A2=Cat & A.3.6-A.3.9Health Insurance=Yes**  **A.21.2: If (one of) your insured cat(s) were suffering from a severe illness, and would have to either undergo treatment (with a good chance of a successful outcome), or be euthanised, what would you do?**  **UK:**   \| **I would ask for euthanasia.** \|  \| \| --- \| --- \| \| **I would spend < £100 above the insurance maximum.** \|  \| \| **I would spend £101-500 above the insurance maximum.** \|  \| \| **I would spend £501-1000 above the insurance maximum.** \|  \| \| **I would spend £1001-3000 above the insurance maximum.** \|  \| \| **I would spend £3001-5000 above the insurance maximum.** \|  \| \| **I would spend £5001-7999 above the insurance maximum.** \|  \| \| **I would spend £8000 or more above the insurance maximum.** \|  \| \| **I don’t know** \|  \| |
| **Instruction Section B: only if A2=Dog & A2=Cat**  **SECTION B:**  **This second part of the questionnaire focuses on the advanced veterinary treatments available in modern veterinary practice.**  **B.1: Advances in small animal practice**  **B.1: To what extent do you agree with the following statements:**  1= strongly disagree; 2 = disagree; 3 = somewhat disagree; 4 = neutral (neither agree nor disagree); 5 = somewhat agree; 6 = agree and 7 = strongly agree   \|  \|  \| 1 \| 2 \| 3 \| 4 \| 5 \| 6 \| 7 \| \| --- \| --- \| --- \| --- \| --- \| --- \| --- \| --- \| --- \| \| 1 \| My pet should have access to the same **diagnostic tests** that are available to human patients \|  \|  \|  \|  \|  \|  \|  \| \| 2 \| My pet should have access to the same **treatment options** that are available to human patients \|  \|  \|  \|  \|  \|  \|  \| \| 3 \| I would enrol my pet in a research study to help advance veterinary care, as long as the risk of potential complications was low. \|  \|  \|  \|  \|  \|  \|  \| \| 4 \| My vet should offer my pet the most advanced treatment that is available. \|  \|  \|  \|  \|  \|  \|  \| \| 5 \| It is important that my vet contributes knowledge to the advancement of veterinary care for future patients. \|  \|  \|  \|  \|  \|  \|  \| \| 6 \| The advanced care available in modern veterinary medicine has gone ‘too far’, putting animals through ‘too much’ \|  \|  \|  \|  \|  \|  \|  \| \| 7 \| The advanced care available in modern veterinary medicine is unnecessary - animals should not be treated in the same way as humans. \|  \|  \|  \|  \|  \|  \|  \| |
| **B.2: Factors related to the patient, the client and veterinarian’s professional environment**  **B.2: In respect of your veterinarian’s approach, to what extent do you agree with the following statements:**  1= strongly disagree; 2 = disagree; 3 = somewhat disagree; 4 = neutral (neither agree nor disagree); 5 = somewhat agree; 6 = agree and 7 = strongly agree   \|  \|  \| 1 \| 2 \| 3 \| 4 \| 5 \| 6 \| 7 \| \| --- \| --- \| --- \| --- \| --- \| --- \| --- \| --- \| --- \| \| 1 \| My pet should always be the priority for my vet when making medical decisions. \|  \|  \|  \|  \|  \|  \|  \| \| 2 \| If my pet was terminally ill, my vet should be willing to provide palliative care to allow me time to say ‘goodbye’. \|  \|  \|  \|  \|  \|  \|  \| \| 3 \| My vet should discuss all appropriate treatment options with me, but it is up to me to decide. \|  \|  \|  \|  \|  \|  \|  \| \| 4 \| My vet should consider my emotional concerns during the decision-making process. \|  \|  \|  \|  \|  \|  \|  \| \| 5 \| My vet should encourage me to provide the best care for my pet, even if it might put me under pressure e.g. time-related or financially. \|  \|  \|  \|  \|  \|  \|  \| \| 6 \| My vet should consider my personal situation / concerns in the decision-making process, even if this means not being able to deliver the best possible treatment for my pet. \|  \|  \|  \|  \|  \|  \|  \| \| 7 \| My vet should only make decisions that are in the best interests of my pet, even if this means sending me to another vet. \|  \|  \|  \|  \|  \|  \|  \| \| 8 \| It is my vet’s responsibility to find the best possible solution for my pet **and** me. \|  \|  \|  \|  \|  \|  \|  \| \| 9 \| My vet should not only act professionally but should also be emotionally supportive of me. \|  \|  \|  \|  \|  \|  \|  \| \| 10 \| If my vet’s opinion differs from mine, they should ultimately let me decide. \|  \|  \|  \|  \|  \|  \|  \| \| 11 \| It is more important that my vet acts in the best interest of my pet, than considers my concerns (e.g. financial issues). \|  \|  \|  \|  \|  \|  \|  \| \| 12 \| In case of conflict, my vet should empathise with my personal feelings during the decision-making process. \|  \|  \|  \|  \|  \|  \|  \| \| 13 \| My vet should not make the decisions - I should make the decisions for my pet \|  \|  \|  \|  \|  \|  \|  \| \| 14 \| My vet should empathise with my situation in the decision-making process. \|  \|  \|  \|  \|  \|  \|  \| \| 15 \| My vet should be willing to make compromises in patient care if I cannot afford the best possible/ most appropriate treatment. \|  \|  \|  \|  \|  \|  \|  \| |
| **B.2.1: Regarding veterinary advice**  **Instruction B.2.1: single-choice question**  **B.2.1:** **Have you ever been in a situation where you wanted to continue with treatment for your pet against your vet’s advice?**   \| Yes \|  \| \| --- \| --- \| \| No \|  \| \| I don’t know \|  \|   **Instruction B.2.2: only if B.2.1=Yes**  **B.2.2: To what extent did the following factors influence your desire to continue treatment against your vet’s advice?**  1= not at all; 2 = slightly; 3 = moderately; 4 = relatively strongly; 5 = very strongly and 6 = I don’t know   \|  \|  \| 1 \| 2 \| 3 \| 4 \| 5 \| 6 \| \| --- \| --- \| --- \| --- \| --- \| --- \| --- \| --- \| \| 1 \| I know my pet better than my vet does \|  \|  \|  \|  \|  \|  \| \| 2 \| Pressure from another person (e.g. family member, friend) \|  \|  \|  \|  \|  \|  \| \| 3 \| I obtained medical information from the internet \|  \|  \|  \|  \|  \|  \| \| 4 \| I believe(d) it to be in the best interests of my animal \|  \|  \|  \|  \|  \|  \| \| 5 \| I have had / heard of a positive experience with another animal having the same treatment \|  \|  \|  \|  \|  \|  \| \| 6 \| I have had / heard of a positive experience with family or friends going through the same treatment \|  \|  \|  \|  \|  \|  \| \| 7 \| I have seen positive reports about the treatment in the press and social media \|  \|  \|  \|  \|  \|  \| \| 8 \| I obtained a second opinion from another veterinarian \|  \|  \|  \|  \|  \|  \| \| 9 \| My animal was insured \|  \|  \|  \|  \|  \|  \|   **Instruction B.2.3: single-choice question**  **B.2.3: Have you ever been in a situation where you refused treatment for your pet that your vet recommended?**   \| Yes \|  \| \| --- \| --- \| \| No \|  \| \| I don’t know \|  \|   **Instruction B.2.4: only if B.2.3=Yes**  **B.2.4: To what extent did the following factors influence your decision to refuse treatment that your vet recommended?**  1 = not at all; 2 = slightly; 3 = moderately; 4 = relatively strongly; 5 = very strongly and 6 = I don’t know   \|  \|  \| 1 \| 2 \| 3 \| 4 \| 5 \| 6 \| \| --- \| --- \| --- \| --- \| --- \| --- \| --- \| --- \| \| 1 \| I could not afford the treatment at the time \|  \|  \|  \|  \|  \|  \| \| 2 \| I felt that my animal was too old \|  \|  \|  \|  \|  \|  \| \| 3 \| I was not strongly emotionally attached to my animal \|  \|  \|  \|  \|  \|  \| \| 4 \| Pressure from another person (e.g. family member, friend) \|  \|  \|  \|  \|  \|  \| \| 5 \| I have / had limited time resources to provide the required care \|  \|  \|  \|  \|  \|  \| \| 6 \| I did not think that the treatment was in my animal’s best interests \|  \|  \|  \|  \|  \|  \| \| 7 \| I have had / heard of a bad experience with another animal going through the same treatment \|  \|  \|  \|  \|  \|  \| \| 8 \| I have had / heard of a bad experience with family or friends going through the same treatment \|  \|  \|  \|  \|  \|  \| \| 9 \| I have seen negative reports about the treatment in the press and social media \|  \|  \|  \|  \|  \|  \| \| 10 \| I wanted to obtain a second opinion from a different veterinarian \|  \|  \|  \|  \|  \|  \| |
| **B.3: Health insurance in veterinary practice**  **Instruction B.3.1: multiple choice question and only if A3.1= Health insurance=yes or A.3.2.1Health insurance=Yes or A.3.2.2Health insurance=Yes or A.3.3.1Health insurance=Yes or A3.3.2Health insurance=Yes or A3.3.3Health insurance=Yes or A.3.4.1Health insurance=Yes or A3.4.2Health insurance=Yes or A3.4.3Health insurance=Yes or A.3.6=Health insurance=Yes or A.3.7.1Health insurance=Yes or A.3.7.2Health insurance=Yes or A.3.8.1Health insurance=Yes or A3.8.2Health insurance=Yes or A3.8.3Health insurance=Yes or A.3.9.1Health insurance=Yes or A3.9.2Health insurance=Yes or A3.9.3Health insurance=Yes**  **B.3.1 Why do you have a health insurance for your pet(s)? Please tick all that apply.**   \| 1 \| My vet advised me to take out health insurance \|  \| \| --- \| --- \| --- \| \| 2 \| I have had insurance for pets previously \|  \| \| 3 \| I have been in the situation of not being able to afford treatment before \|  \| \| 4 \| Friends advised me to take out health insurance \|  \| \| 5 \| I believe it is part of being a responsible owner \|  \| \| 6 \| I may not be able to afford veterinary care for my pet without it \|  \| \| 7 \| Because my pet is old and at greater risk of illness \|  \| \| 8 \| To avoid having to make difficult financial decisions if my pet becomes ill \|  \| \| 9 \| Insurance was included free /very cheap when I first obtained my pet (e.g. puppy package) \|  \| \| 10 \| I don’t know \|  \| \| 11 \| Other \|  \|   **Instruction B.3.2: multiple choice question and only if A3.1Health insurance=No or A.3.2.1Health insurance=No or A.3.2.2Health insurance=No or A.3.3.1=Health insurance=No or A3.3.2Health insurance=No or A3.3.3Health insurance=No or A.3.4.1Health insurance=No or A3.4.2Health insurance=No or A3.4.3Health insurance=No or A.3.6Health insurance=No or A.3.7.1Health insurance=No or A.3.7.2Health insurance=No or A.3.8.1Health insurance=No or A3.8.2Health insurance=No or A3.8.3Health insurance=No or A.3.9.1Health insurance=No or A3.9.2Health insurance=No or A3.9.3Health insurance=No**  **B.3.2 Why do you not have health insurance for your pet(s)? Please tick all that apply.**   \| 1 \| I had not heard about it \|  \| \| --- \| --- \| --- \| \| 2 \| I do not trust insurance policies \|  \| \| 3 \| Pet insurance policies are too expensive \|  \| \| 4 \| My vet advised me not to purchase health insurance \|  \| \| 5 \| The benefits are not worth the cost \|  \| \| 6 \| It was too difficult to find a suitable policy \|  \| \| 7 \| My pet is too old, and so it is not relevant anymore \|  \| \| 8 \| My pet is too old, and insurance has become too expensive \|  \| \| 9 \| I don’t know \|  \| \| 10 \| Other \|  \|   **Instruction B.3.3: multiple choice question and only if A3.1Health insurance=not anymore A.3.2.1Health insurance=Not anymore or A.3.2.2Health insurance=Not anymore or A.3.3.1=Health insurance=Not anymore or A3.3.2Health insurance=Not anymore or A3.3.3Health insurance=Not anymore or A.3.4.1Health insurance=Not anymore or A3.4.2Health insurance=Not anymore or A3.4.3Health insurance=Not anymore or A3.6Health insurance=not anymore or A.3.7.1Health insurance=Not anymore or A.3.7.2Health insurance=Not anymore or A.3.8.1Health insurance=Not anymore or A3.8.2Health insurance=Not anymore or A3.8.3Health insurance=Not anymore or A.3.9.1Health insurance=Not anymore or A3.9.2Health insurance=Not anymore or A3.9.3Health insurance=Not anymore**  **B.3.3 Why did you not continue to insure your pet(s)? Please tick all that apply.**   \| 1 \| The benefits were not worth the expense \|  \| \| --- \| --- \| --- \| \| 2 \| It became too expensive \|  \| \| 3 \| Too many exclusions were added \|  \| \| 4 \| Not all costs were covered \|  \| \| 5 \| I don’t know \|  \| \| 6 \| Other \|  \| |
| **B.4.: The use of social media by clients and veterinary practices**  **Social media are forms of electronic communication (e.g. Facebook, Twitter, Instagram) through which users create online communities to share information, ideas, personal messages, and other content (such as videos, photos).**  **Instruction B.4.1: multiple choice question**  **B.4.1: What sort of social media do you use, either casually or as a registered user*?* Tick all that apply.**   \| 1 \| I do not use any social media \|  \| \| --- \| --- \| --- \| \| 2 \| Facebook \|  \| \| 3 \| Instagram \|  \| \| 4 \| Twitter \|  \| \| 5 \| YouTube \|  \| \| 6 \| TikTok \|  \| \| 7 \| Other \|  \|   **Instruction B.4.2: single-choice choice question and only if B4.1=Facebook, B4.1=Instagram, B4.1=Twitter, B4.1=YouTube, B4.1=TikTok and/or B4.1=other**  **B.4.2: How often do you use social media?**   \| Every day \|  \| \| --- \| --- \| \| 4-6 days per week \|  \| \| 1-3 days per week \|  \| \| Less than one day per week \|  \| \| Less frequently \|  \| \| I don’t know \|  \|   **Instruction B.4.3: single-choice choice question**  **B.4.3:** **Do you expect your veterinary practice to have an active presence on social media?**     \| Yes \|  \| \| --- \| --- \| \| No \|  \| \| I don’t know \|  \|   **B.4.4: To what extent do you agree with the following statements?**  1= strongly disagree; 2 = disagree; 3 = somewhat disagree; 4 = neutral (neither agree nor disagree); 5 = somewhat agree; 6 = agree; 7 = strongly agree and 8 = I don’t know   \|  \| **I think that the use of social media by veterinary practices…** \| 1 \| 2 \| 3 \| 4 \| 5 \| 6 \| 7 \| 8 \| \| --- \| --- \| --- \| --- \| --- \| --- \| --- \| --- \| --- \| --- \| \| 1 \| gives people an insight into what happens in the practice. \|  \|  \|  \|  \|  \|  \|  \|  \| \| 2 \| is not necessary. \|  \|  \|  \|  \|  \|  \|  \|  \| \| 3 \| is a useful way for pet owners to find a (new) practice. \|  \|  \|  \|  \|  \|  \|  \|  \| \| 4 \| enables owners to stay in touch with the veterinary practice and/or the veterinarian in an informal and easy way. \|  \|  \|  \|  \|  \|  \|  \|  \|   **Instruction B.4.5: single-choice choice question**  **B.4.5:** **Have you ever left (a) negative comment(s) / complaint(s) about your vet / practice on the internet (e.g. webpages or social media)?**   \| Yes \|  \| \| --- \| --- \| \| No \|  \|   **Instruction B.4.6: multiple choice question and only if B.4.5=yes**  **B.4.6: If yes, what was/were the negative comment(s) / complaint(s) about? Please tick all that apply.***)*   \| too long a waiting time \|  \| \| --- \| --- \| \| the veterinarian’s medical advice \|  \| \| the way my pet was handled by the staff \|  \| \| the outcome of my pet’s treatment / complications \|  \| \| the cost of treatment \|  \| \| behaviour of specific staff member(s) \|  \| \| the lack of technical equipment in the practice \|  \| \| unfriendly communication by the veterinarian \|  \| \| too technical or complicated way of communicating by the veterinarian \|  \| \| unfriendly communication by other staff \|  \| \| Other \|  \|   **Instruction B.4.7: single-choice choice question**  **B.4.7:** **Did your vet respond to the negative feedback / complaint?**   \| Yes \|  \| \| --- \| --- \| \| No \|  \| \| I don’t know \|  \| |
| **B.5: Using internet resources to find veterinary medical information**  **Instruction B.5.1: single-choice choice question**  **B.5.1: How often do you use internet resources to find medical information PRIOR to a consultation with your vet?**   \| Never \|  \| \| --- \| --- \| \| Occasionally \|  \| \| Frequently \|  \| \| Always \|  \| \| I don’t know \|  \|   **Instruction B.5.2: single-choice choice question**  **B.5.2: How often do you use internet resources to find medical information AFTER consultation with your vet?**   \| Never \|  \| \| --- \| --- \| \| Occasionally \|  \| \| Frequently \|  \| \| Always \|  \| \| I don’t know \|  \|   **Instruction B.5.3: multiple choice question and only if B.5.1 or B.5.2=occasionally, B.5.1 or B.5.2=frequently, B.5.1 or B.5.2=always and/or B.5.1 or B.5.2=I don’t know**  **B.5.3: Which resources have you used?**   \| 1 \| Blogs and chat rooms \|  \| \| --- \| --- \| --- \| \| 2 \| Websites providing veterinary medical information \|  \| \| 3 \| Social media (e.g. Facebook, Twitter) \|  \| \| 4 \| Practice website \|  \| \| 5 \| Veterinary association websites (e.g. BVA, BSAVA, RCVS) \|  \| \| 6 \| University website \|  \| \| 7 \| Other \|  \|   **Instruction B.5.4: only if B.5.1 or B.5.2=occasionally, B.5.1 or B.5.2=frequently, B.5.1 or B.5.2=always and/or B.5.1 or B.5.2=I don’t know**  **B.5.4:** **To what extent do you agree with the following statements?**  1= strongly disagree; 2 = disagree; 3 = somewhat disagree; 4 = neutral (neither agree nor disagree); 5 = somewhat agree; 6 = agree; 7 = strongly agree and 8 = I don’t know   \|  \| **The use of internet resources…** \| 1 \| 2 \| 3 \| 4 \| 5 \| 6 \| 7 \| 8 \| \| --- \| --- \| --- \| --- \| --- \| --- \| --- \| --- \| --- \| --- \| \| 1 \| increases my expectations of the standard of veterinary care available for my pet. \|  \|  \|  \|  \|  \|  \|  \|  \| \| 2 \| enables me to have a more informed discussion with my vet. \|  \|  \|  \|  \|  \|  \|  \|  \| \| 3 \| can lead to situations where I am better informed than my vet. \|  \|  \|  \|  \|  \|  \|  \|  \| \| 4 \| enables me to challenge my vet to justify their recommendations. \|  \|  \|  \|  \|  \|  \|  \|  \| \| 5 \| helps me to make the right decision for my animal. \|  \|  \|  \|  \|  \|  \|  \|  \| \| 6 \| enables me to buy some medication(s) more cheaply (e.g. flea treatments, wormers). \|  \|  \|  \|  \|  \|  \|  \|  \| \| 7 \| can give the wrong impression of standard veterinary medicine \|  \|  \|  \|  \|  \|  \|  \|  \|   **Instruction B.5.5: single-choice question and only if B.5.1 or B.5.2=occasionally, B.5.1 or B.5.2=frequently, B.5.1 or B.5.2=always and/or B.5.1 or B.5.2=I don’t know**  **B.5.5: Have you ever disagreed with your vet’s professional advice based on information you obtained from the internet?**   \| Yes \|  \| \| --- \| --- \| \| No \|  \| \| I don’t know \|  \| |
| **B.6: Telemedicine in modern small animal practice**  **Telemedicine, defined as the practice of using electronic media to deliver advice and care ‘at a distance’, has become an option in modern small animal practice. This enables animal owners to have a full consultation with a vet - for which they are charged - without having to take their pet to the practice.**  **Instruction B.6.1: single-choice question**  **B.6.1: Have you ever used telemedicine to obtain veterinary advice about your pet(s)?**   \| Yes \|  \| \| --- \| --- \| \| No \|  \| \| I didn’t know it was an option \|  \|     **Instruction B.6.2: single-choice question and only if B.6.1=No or B.6.1=I didn’t know it was an option**  **B.6.2: Would you make use of telemedicine if your vet offered it instead of an in-person consultation?**   \| Yes \|  \| \| --- \| --- \| \| No \|  \| \| I don’t know \|  \|   **B.6.3: To what extent do you agree with the following statements?**  1= strongly disagree; 2 = disagree; 3 = somewhat disagree; 4 = neutral (neither agree nor disagree); 5 = somewhat agree; 6 = agree; 7 = strongly agree and 8 = I don’t know   \|  \| **The use of telemedicine…** \| 1 \| 2 \| 3 \| 4 \| 5 \| 6 \| 7 \| 8 \| \| --- \| --- \| --- \| --- \| --- \| --- \| --- \| --- \| --- \| --- \| \| 1 \| could be helpful, as I find it difficult to travel to a vet. \|  \|  \|  \|  \|  \|  \|  \|  \| \| 2 \| could save my pet from a stressful journey to the vet. \|  \|  \|  \|  \|  \|  \|  \|  \| \| 3 \| could help me decide whether my pet needs to see a vet. \|  \|  \|  \|  \|  \|  \|  \|  \| \| 4 \| could be useful for follow-up appointment. \|  \|  \|  \|  \|  \|  \|  \|  \| \| 5 \| could improve my access to a specialist if there are none locally. \|  \|  \|  \|  \|  \|  \|  \|  \| \| 6 \| is good in case of emergencies. \|  \|  \|  \|  \|  \|  \|  \|  \| \| 7 \| weakens the veterinarian-client bond. \|  \|  \|  \|  \|  \|  \|  \|  \| \| 8 \| enhances the veterinarian-client bond. \|  \|  \|  \|  \|  \|  \|  \|  \| \| 9 \| should cost less than a normal consultation. \|  \|  \|  \|  \|  \|  \|  \|  \| \| 10 \| has no benefit. \|  \|  \|  \|  \|  \|  \|  \|  \| \| 11 \| increases the risk of something being missed because the animal is not physically examined by the vet. \|  \|  \|  \|  \|  \|  \|  \|  \| \| 12 \| is more convenient than attending in person. \|  \|  \|  \|  \|  \|  \|  \|  \| \| 13 \| is not an option for me as I don’t have the necessary technical IT skills or equipment. \|  \|  \|  \|  \|  \|  \|  \|  \| |
| **C: Social Support**  **Finally, we kindly ask you to provide some information concerning your social environment and support network.**  **C.1: If you needed it, how often is someone available…**  *1=never, 2=occasionally, 3= mostly and 4= always*   \|  \| \| **1** \| **2** \| **3** \| **4** \| \| --- \| --- \| --- \| --- \| --- \| --- \| \| 1 \| to take you to the doctor if you need it. \|  \|  \|  \|  \| \| 2 \| to prepare your meals if you are unable to do it yourself. \|  \|  \|  \|  \| \| 3 \| to help with daily chores if you were sick. \|  \|  \|  \|  \| \| 4 \| to give you good advice about a crisis. \|  \|  \|  \|  \| \| 5 \| to confide in or talk to about yourself or your problems. \|  \|  \|  \|  \| \| 6 \| who understands your problems. \|  \|  \|  \|  \|   **Instruction C.2: single-choice question and list in drop-down menu**  **C.2: How often do you feel that you have no one to talk to?**   \| Hardly ever or never \|  \| \| --- \| --- \| \| Some of the time \|  \| \| Often \|  \|   **Instruction C.3: single-choice question and list in drop-down menu**  **C.3: How often do you feel left out?**   \| Hardly ever or never \|  \| \| --- \| --- \| \| Some of the time \|  \| \| Often \|  \|   **Instruction C.4: single-choice question and list in drop-down menu**  **C.4: How often do you feel isolated from others?**   \| Hardly ever or never \|  \| \| --- \| --- \| \| Some of the time \|  \| \| Often \|  \|   **Instruction C.5: single-choice question and list in drop-down menu**  **C.5: How often do you feel lonely?**   \| Often/always \|  \| \| --- \| --- \| \| Some of the time \|  \| \| Occasionally \|  \| \| Hardly ever \|  \| \| Never \|  \| |
| **Thank you for participating in the survey!** |

| **Einführung und Hintergrund:**  **Herzlichen Dank für Ihre Teilnahme an dieser Umfrage.**  **Ziel** dieser Umfrage ist es, **herauszufinden**, **warum manche Menschen Haustiere halten und andere nicht,** und **zu untersuchen**, welche **Einstellung** **HaustierbesitzerInnen** **zu der** **modernen** **Kleintierpraxis** haben. Das Ausfüllen des **Fragebogens** wird **in etwa drei Minuten Ihrer Zeit** in Anspruch nehmen, wenn Sie **keine Haustiere** haben. **Wenn Sie ein Haustier haben,** stellen wir Ihnen auch Fragen zu **Ihrer Bindung zu dem Haustier** (oder den Haustieren) sowie zu **Ihrer Einstellung zur modernen veterinärmedizinischen Praxis** und **Ihren Erwartungen hinsichtlich tierärztlicher Dienstleistungen.** Außerdem erwarten Sie einige Fragen zur Krankenversicherung von Haustieren sowie zur Nutzung von sozialen Medien und Internetquellen im Zusammenhang mit tierärztlicher Behandlung. Für **HaustierbesitzerInnen** wird das Ausfüllen des **Fragebogens in etwa 15-20 Minuten** dauern.    Diese Umfrage ist Teil eines Forschungsprojekts, an dem drei Länder beteiligt sind: Dänemark, Österreich und das Vereinigte Königreich. Die beteiligten Forscherinnen und Forscher arbeiten an der Universität Kopenhagen, an der Veterinärmedizinischen Universität Wien und an der Universität Glasgow.  Die **Teilnahme** an dieser Umfrage ist **freiwillig** und **Sie können diese jederzeit verlassen.** Ihre Antworten werden in **anonymisierter** Form an das Forschungsteam weitergeleitet. **Aus den Angaben ist kein Rückschluss auf Ihre Identität möglich.**  Durch das Klicken auf „Weiter“ bestätigen Sie, dass Sie über 17 Jahre alt sind und der Teilnahme an dieser Umfrage **einwilligen**.  **Herzlichen Dank für Ihren Beitrag!** |
| --- |
| **ABSCHNITT A:**  **Angaben zu Ihrem Haustier, demografische Angaben und Informationen zu der Tierarztpraxis / Tierklinik, die Sie aufsuchen**  **Instruction A.1: single-choice question**  **A.1: Haustiere werden oft zu Hause gehalten, aber unser Interesse gilt auch Tieren, die nicht zu Hause gehalten werden (z.B. Pferde). Landwirtschaftliche Nutztiere, z.B. Milchkühe, sind für unsere Studie nicht relevant.**  **Haben Sie ein oder mehrere Haustier(e)?**   \| Ja \|  \| \| --- \| --- \| \| Nein \|  \|   **Instruction A.2: multiple choice question and only if A1=Ja**  **A.2: Wie viele der folgenden Haustiere haben Sie? Bitte geben Sie die Anzahl der einzelnen Arten an, oder wählen Sie gegebenenfalls 0 (keines).**   \|  \| 0 (keines) \| 1 \| 2 \| 3 \| 4 \| Mehr als 4 \| \| --- \| --- \| --- \| --- \| --- \| --- \| --- \| \| Hund \|  \|  \|  \|  \|  \|  \| \| Katze \|  \|  \|  \|  \|  \|  \| \| Pferd \|  \|  \|  \|  \|  \|  \| \| Hase / Kaninchen \|  \|  \|  \|  \|  \|  \| \| Nagetier (z.B. Hamster, Meerschweinchen, Chinchilla, Maus/Ratte) \|  \|  \|  \|  \|  \|  \| \| Vogel \|  \|  \|  \|  \|  \|  \| \| Reptil (z.B. Eidechse, Schlange, Schildkröte) \|  \|  \|  \|  \|  \|  \| \| Fische (im Aquarium oder in einem Teich im Garten) \|  \|  \|  \|  \|  \|  \| \| Anderes Haustier als die Obengenannten \|  \|  \|  \|  \|  \|  \|   *Construct filter variable =* ***Number of pet species***  *Count =* ***Number of pet species*** *if A2Hund>0, A2Katze>0, A2Pferd>0, A2Hase/Kaninchen>0, A2Nagetier>0, A2Vogel>0, A2Reptil>0, A2Fische>0, A2* Anderes Haustier als die Obengenannten *>0*  **Instruction A.3.1: only if A2Hund=1**  **A.3.1: Bitte geben Sie das Alter Ihres Hundes und die Anzahl der Tierarztbesuche in den letzten 12 Monaten an, sowie ob der Hund krankenversichert ist.**  Scrolldown-Menu: Alter (<0,5–> 20 Jahre)  Scrolldown-Menu: Anzahl der Tierarztbesuche in den letzten 12 Monaten (0–> 20)  Scrolldown-Menu: Krankenversicherung (ja, nein, nicht mehr)  **Instruction A.3.1.1: single-choice question and only if A3.1. Krankenversicherung=Ja**  **A.3.1.1: Was ist die Versicherungsgrenze für Ihren Hund (inklusive OP-Versicherung, falls vorhanden)?**  Scrolldown-Menu:   \| **Ich weiß nicht** \| \| --- \| \| **Bis zu 1500€ pro Jahr** \| \| **1501–2500€ pro Jahr** \| \| **2501-5000€ pro Jahr** \| \| **5001–8000€ pro Jahr** \| \| **Mehr als 8000€ pro Jahr** \|   **Instruction A.3.2: only if A2Hund=2**  **A.3.2: Bitte geben Sie das Alter Ihrer Hunde und die Anzahl der Tierarztbesuche in den letzten 12 Monaten an, sowie ob die beiden Hunde krankenversichert sind.**  **A.3.2.1:**  Scrolldown-Menu: Alter (<0,5–> 20 Jahre)  Scrolldown-Menu: Anzahl der Tierarztbesuche in den letzten 12 Monaten (0–> 20)  Scrolldown-Menu: Krankenversicherung (ja, nein, nicht mehr)  **A.3.2.2:**  Scrolldown-Menu: Alter (<0,5–> 20 Jahre)  Scrolldown-Menu: Anzahl der Tierarztbesuche in den letzten 12 Monaten (0–> 20)  Scrolldown-Menu: Krankenversicherung (ja, nein, nicht mehr)  **Instruction A.3.3: only if A2Hund=3**  **A.3.3: Bitte geben Sie das Alter Ihrer Hunde und die Anzahl der Tierarztbesuche in den letzten 12 Monaten an, sowie ob die drei Hunde krankenversichert sind.**  **A.3.3.1:**  Scrolldown-Menu: Alter (<0,5–> 20 Jahre)  Scrolldown-Menu: Anzahl der Tierarztbesuche in den letzten 12 Monaten (0–> 20)  Scrolldown-Menu: Krankenversicherung (ja, nein, nicht mehr)  **A.3.3.2:**  Scrolldown-Menu: Alter (<0,5–> 20 Jahre)  Scrolldown-Menu: Anzahl der Tierarztbesuche in den letzten 12 Monaten (0–> 20)  Scrolldown-Menu: Krankenversicherung (ja, nein, nicht mehr)  **A.3.3.3:**  Scrolldown-Menu: Alter (<0,5–> 20 Jahre)  Scrolldown-Menu: Anzahl der Tierarztbesuche in den letzten 12 Monaten (0–> 20)  Scrolldown-Menu: Krankenversicherung (ja, nein, nicht mehr)  **Instruction A.3.4: only if A2Hund>3**  **A.3.4: Die folgenden Fragen beziehen sich auf das Alter von dreien Ihrer Hunde, die Anzahl der Tierarztbesuche in den letzten 12 Monaten und darauf, ob die drei Hunde krankenversichert sind. Bitte wählen Sie jene drei Hunde aus, deren Namen in alphabetischer Reihenfolge als Erste kommen.**  **A.3.4.1:** Erster Hund (Anfangsbuchstabe des Namens kommt in alphabetischer Reihenfolge als Erster)  Scrolldown-Menu: Alter (<0,5–> 20 Jahre)  Scrolldown-Menu: Anzahl der Tierarztbesuche in den letzten 12 Monaten (0–> 20)  Scrolldown-Menu: Krankenversicherung (ja, nein, nicht mehr)  **A.3.4.2:** Zweiter Hund (Anfangsbuchstabe des Namens kommt in alphabetischer Reihenfolge als Zweiter)  Scrolldown-Menu: Alter (<0,5–> 20 Jahre)  Scrolldown-Menu: Anzahl der Tierarztbesuche in den letzten 12 Monaten (0–> 20)  Scrolldown-Menu: Krankenversicherung (ja, nein, nicht mehr)  **A.3.4.3:** Dritter Hund (Anfangsbuchstabe des Namens kommt in alphabetischer Reihenfolge als Dritter)  Scrolldown-Menu: Alter (<0,5–> 20 Jahre)  Scrolldown-Menu: Anzahl der Tierarztbesuche in den letzten 12 Monaten (0–> 20)  Scrolldown-Menu: Krankenversicherung (ja, nein, nicht mehr)  **Instruction A.3.5: German questionnaire: only if A.3.2.1Krankenversicherung=Ja or A.3.2.2 Krankenversicherung=Ja or A.3.3.1 Krankenversicherung=Ja or A3.3.2 Krankenversicherung=Ja or A3.3.3 Krankenversicherung=Ja or A.3.4.1 Krankenversicherung=Ja or A3.4.2 Krankenversicherung=Ja or A3.4.3 Krankenversicherung=Ja**  **A.3.5: Was ist die Versicherungsgrenze für Ihre(n) Hund(e) (inklusive OP-Versicherung, falls vorhanden)?** ***Wenn der Grenzwert nicht für alle Hunde gleich ist, dann denken Sie an den Hund, dessen Name im Alphabet an erster Stelle steht.***   \| **Ich weiß nicht** \| \| --- \| \| **Bis zu 1500€ pro Jahr** \| \| **1501–2500€ pro Jahr** \| \| **2501-5000€ pro Jahr** \| \| **5001–8000€ pro Jahr** \| \| **Mehr als 8000€ pro Jahr** \|   **Instruction A.3.6: only if A2Cat=1**  **A.3.6: Bitte geben Sie das Alter Ihrer Katze und die Anzahl der Tierarztbesuche in den letzten 12 Monaten an, sowie ob die Katze krankenversichert ist.**  Scrolldown-Menu: Alter (<0,5–> 20 Jahre)  Scrolldown-Menu: Anzahl der Tierarztbesuche in den letzten 12 Monaten (0–> 20)  Scrolldown-Menu: Krankenversicherung (ja, nein, nicht mehr)  **Instruction A.3.6.1: single-choice question and only if A3.6 Krankenversicherung=Ja**  **A.3.6.1: Was ist die Versicherungsgrenze für Ihre Katze (inklusive OP-Versicherung, falls vorhanden)?**  Scrolldown-Menu:   \| **Ich weiß nicht** \| \| --- \| \| **Bis zu 1500€ pro Jahr** \| \| **1501–2500€ pro Jahr** \| \| **2501-5000€ pro Jahr** \| \| **5001–8000€ pro Jahr** \| \| **Mehr als 8000€ pro Jahr** \|   **Instruction A.3.7: only if A2Katze=2**  **A.3.7: Bitte geben Sie das Alter Ihrer Katzen und die Anzahl der Tierarztbesuche in den letzten 12 Monaten an, sowie ob die beiden Katzen krankenversichert sind.**  **A.3.7.1:**  Scrolldown-Menu: Alter (<0,5–> 20 Jahre)  Scrolldown-Menu: Anzahl der Tierarztbesuche in den letzten 12 Monaten (0–> 20)  Scrolldown-Menu: Krankenversicherung (ja, nein, nicht mehr)  **A.3.7.2:**  Scrolldown-Menu: Alter (<0,5–> 20 Jahre)  Scrolldown-Menu: Anzahl der Tierarztbesuche im letzten Jahr (0–> 20)  Scrolldown-Menu: Krankenversicherung (ja, nein, nicht mehr)  **Instruction A3.8: only if A2Katze=3**  **A.3.8: Bitte geben Sie das Alter Ihrer Katzen und die Anzahl der Tierarztbesuche in den letzten 12 Monaten an, sowie ob die drei Katzen krankenversichert sind.**  **A.3.8.1:**  Scrolldown-Menu: Alter (<0,5–> 20 Jahre)  Scrolldown-Menu: Anzahl der Tierarztbesuche in den letzten 12 Monaten (0–> 20)  Scrolldown-Menu: Krankenversicherung (ja, nein, nicht mehr)  **A.3.8.2:**  Scrolldown-Menu: Alter (<0,5–> 20 Jahre)  Scrolldown-Menu: Anzahl der Tierarztbesuche in den letzten 12 Monaten (0–> 20)  Scrolldown-Menu: Krankenversicherung (ja, nein, nicht mehr)  **A.3.8.3:**  Scrolldown-Menu: Alter (<0,5–> 20 Jahre)  Scrolldown-Menu: Anzahl der Tierarztbesuche in den letzten 12 Monaten (0–> 20)  Scrolldown-Menu: Krankenversicherung (ja, nein, nicht mehr)  **Instruction A.3.9: only if A2Katze>3**  **A.3.8: Die folgenden Fragen beziehen sich auf das Alter von dreien Ihrer Katzen, die Anzahl der Tierarztbesuche in den letzten 12 Monaten und darauf, ob die drei Katzen krankenversichert sind. Bitte wählen Sie jene drei Katzen aus, deren Namen in alphabetischer Reihenfolge als Erste kommen.**  **A.3.8.1:** Erste Katze (Anfangsbuchstabe des Namens kommt in alphabetischer Reihenfolge als Erster)  Scrolldown-Menu: Alter (<0,5–> 20 Jahre)  Scrolldown-Menu: Anzahl der Tierarztbesuche in den letzten 12 Monaten (0–> 20)  Scrolldown-Menu: Krankenversicherung (ja, nein, nicht mehr)  **A.3.8.2:** Zweite Katze (Anfangsbuchstabe des Namens kommt in alphabetischer Reihenfolge als Zweiter)  Scrolldown-Menu: Alter (<0,5–> 20 Jahre)  Scrolldown-Menu: Anzahl der Tierarztbesuche in den letzten 12 Monaten (0–> 20)  Scrolldown-Menu: Krankenversicherung (ja, nein, nicht mehr)  **A.3.8.3:** Dritte Katze (Anfangsbuchstabe des Namens kommt in alphabetischer Reihenfolge als Dritter)  Scrolldown-Menu: Alter (<0,5–> 20 Jahre)  Scrolldown-Menu: Anzahl der Tierarztbesuche in den letzten 12 Monaten (0–> 20)  Scrolldown-Menu: Krankenversicherung (ja, nein, nicht mehr)  **Instruction A.3.10: German questionnaire: only if A.3.7.1Krankenversicherung=Ja or A.3.7.2 Krankenversicherung=Ja or A.3.8.1 Krankenversicherung=Ja or A3.8.2 Krankenversicherung=Ja or A3.8.3 Krankenversicherung=Ja or A.3.9.1 Krankenversicherung=Ja or A3.9.2 Krankenversicherung=Ja or A3.9.3 Krankenversicherung=Ja**  **A.3.10: Was ist die Versicherungsgrenze für Ihre Katze(n) (inklusive OP-Versicherung, falls vorhanden)?** ***Wenn der Grenzwert nicht für alle Katzen gleich ist, dann denken Sie an die Katze, deren Name im Alphabet an erster Stelle steht.***  Scrolldown-Menu:   \| **Ich weiß nicht** \| \| --- \| \| **Bis zu 1500€ pro Jahr** \| \| **1501–2500€ pro Jahr** \| \| **2501–5000€ pro Jahr** \| \| **5001–8000€ pro Jahr** \| \| **Mehr als 8000€ pro Jahr** \|   **Instruction A.4: multiple choice question and only if A1=Nein**  **A.4: Warum haben Sie keinen Hund und/oder keine Katze?**   \| Ich mag keine Hunde und/oder Katzen. \|  \| \| --- \| --- \| \| Eine Person in meinem Haushalt mag keine Hunde und/oder Katzen. \|  \| \| Ich habe nicht genug Zeit, um mich um eine(n) Hund und/oder Katze zu kümmern. \|  \| \| Ich kann es mir nicht leisten, für eine(n) Hund und/oder Katze zu sorgen. \|  \| \| Ich/eine Person in meinem Haushalt habe/hat eine Allergie. \|  \| \| In meiner Unterkunft darf ich keine(n) Hund und/oder Katze halten. \|  \| \| Ich wohne in einer Wohnung und halte es nicht für gerecht, einen Hund oder eine Katze in dieser Umgebung zu halten. \|  \| \| Ich bin der Meinung, dass Menschen überhaupt keine Haustiere halten sollten, weil das die Rechte der Tiere verletzt. \|  \| \| Andere Gründe \|  \|   **Instruction A.4.1: open text field and only if A4=Other reasons**  **Sie können gerne erklären, warum Sie keinen Hund oder keine Katzen haben.**   \| **Offene Antwortmöglichkeit:** \| \| \| --- \| --- \| \| **Ich habe keine weiteren Anmerkungen.** \|  \|   **Instruction A.5: drop-down menu from 18 - 100 Jahre + “Möchte ich nicht angeben”**  **A.5: Bitte wählen Sie Ihr Alter aus dem Dropdown-Menü aus.**  ________ Jahre  **Instruction A.6: single-choice question**  **A.6: Bitte geben Sie Ihr Geschlecht an.**   \| Männlich \|  \| \| --- \| --- \| \| Weiblich \|  \| \| Keines davon \|  \| \| Möchte ich nicht angeben \|  \|   **Instruction A.7: single-choice question**  **A.7: Bitte geben Sie das Bundesland an, in dem Sie hauptsächlich wohnen:**   \| Burgenland \|  \| \| --- \| --- \| \| Kärnten \|  \| \| Land Salzburg \|  \| \| Niederösterreich \|  \| \| Oberösterreich \|  \| \| Steiermark \|  \| \| Tirol \|  \| \| Vorarlberg \|  \| \| Wien \|  \|   **Instruction A.8: single-choice question**  **A.8: Arbeiten/Arbeiteten Sie im Bereich der Veterinär- oder Humanmedizin?**   \| Ja \|  \| \| --- \| --- \| \| Nein \|  \|   **Instruction A.10.1: single-choice question**  **A.10.1: Wohnen Sie alleine (Haustiere nicht mit einberechnet)?**   \| Ja \|  \| \| --- \| --- \| \| Nein \|  \|   **Instruction A.10.2: single-choice question and only if A10.1=Nein and list in drop-down-menu**  **A.10.2: Mit wem wohnen Sie zusammen?**   \| Ich wohne mit (einem/einer) anderen Erwachsenen. \|  \| \| --- \| --- \| \| Ich wohne mit (einem/einer) anderen Erwachsenen und einem Kind unter 18 Jahren (ständig). \|  \| \| Ich wohne mit (einem/einer) anderen Erwachsenen und einem Kind unter 18 Jahren (teilweise). \|  \| \| Ich wohne mit (einem/einer) anderen Erwachsenen und Kindern unter 18 Jahren (ständig). \|  \| \| Ich wohne mit (einem/einer) anderen Erwachsenen und Kindern unter 18 Jahren (teilweise). \|  \| \| Ich wohne mit einem Kind unter 18 Jahren (ständig). \|  \| \| Ich wohne mit einem Kind unter 18 Jahren (teilweise). \|  \| \| Ich wohne mit Kindern unter 18 Jahren (ständig). \|  \| \| Ich wohne mit Kindern unter 18 Jahren (teilweise). \|  \| \| Andere \|  \|   **Instruction A11.1: single-choice question and only if A2=Hund & A10.2 = “ Ich wohne mit (einem/einer) anderen Erwachsenen.” or “ Ich wohne mit (einem/einer) anderen Erwachsenen und einem Kind unter 18 Jahren (ständig).” or “ Ich wohne mit (einem/einer) anderen Erwachsenen und einem Kind unter 18 Jahren (teilweise).“ or “Ich wohne mit (einem/einer) anderen Erwachsenen und Kindern unter 18 Jahren (ständig)“ or “ Ich wohne mit (einem/einer) anderen Erwachsenen und Kindern unter 18 Jahren (teilweise)“ or „Ich wohne mit einem Kind unter 18 Jahren (ständig).“ or „Ich wohne mit einem Kind unter 18 Jahren (teilweise).“ or „Ich wohne mit Kindern unter 18 Jahren (ständig).“ or „Ich wohne mit Kindern unter 18 Jahren (teilweise).“**  **A.11.1: Für wen wurde(n) der/die Hund(e) angeschafft?**   \| Alle (oder die meisten) im Haushalt \|  \| \| --- \| --- \| \| Mich \|  \| \| Meine(n) Partner/in \|  \| \| Mein Kind/Stiefkind \|  \| \| Meine Kinder/Stiefkinder \|  \| \| Für (ein) andere(s) Haustier(e) im Haushalt (zur Gesellschaft) \|  \| \| Andere \|  \| \| Ich weiß nicht \|  \|   **Instruction A11.2: single-choice question and only if A2=Katze & A10.2 = “ Ich wohne mit (einem/einer) anderen Erwachsenen.” or “ Ich wohne mit (einem/einer) anderen Erwachsenen und einem Kind unter 18 Jahren (ständig).” or “ Ich wohne mit (einem/einer) anderen Erwachsenen und einem Kind unter 18 Jahren (teilweise).“ or “Ich wohne mit (einem/einer) anderen Erwachsenen und Kindern unter 18 Jahren (ständig)“ or “ Ich wohne mit (einem/einer) anderen Erwachsenen und Kindern unter 18 Jahren (teilweise)“ or „Ich wohne mit einem Kind unter 18 Jahren (ständig).“ or „Ich wohne mit einem Kind unter 18 Jahren (teilweise).“ or „Ich wohne mit Kindern unter 18 Jahren (ständig).“ or „Ich wohne mit Kindern unter 18 Jahren (teilweise).“**  **A.11.2: Für wen wurde(n) die Katze(n) angeschafft?**   \| Alle (oder die meisten) im Haushalt \|  \| \| --- \| --- \| \| Mich \|  \| \| Meine(n) Partner/in \|  \| \| Mein Kind/Stiefkind \|  \| \| Meine Kinder/Stiefkinder \|  \| \| Für (ein) andere(s) Haustier(e) im Haushalt (zur Gesellschaft) \|  \| \| Andere \|  \| \| Ich weiß nicht \|  \|   **Instruction A12.1: single-choice question and only if A2=Hund & A10.2 = “ Ich wohne mit (einem/einer) anderen Erwachsenen.” or “ Ich wohne mit (einem/einer) anderen Erwachsenen und einem Kind unter 18 Jahren (ständig).” or “ Ich wohne mit (einem/einer) anderen Erwachsenen und einem Kind unter 18 Jahren (teilweise).“ or “Ich wohne mit (einem/einer) anderen Erwachsenen und Kindern unter 18 Jahren (ständig)“ or “ Ich wohne mit (einem/einer) anderen Erwachsenen und Kindern unter 18 Jahren (teilweise)“ or „Ich wohne mit einem Kind unter 18 Jahren (ständig).“ or „Ich wohne mit einem Kind unter 18 Jahren (teilweise).“ or „Ich wohne mit Kindern unter 18 Jahren (ständig).“ or „Ich wohne mit Kindern unter 18 Jahren (teilweise).“**  **A.12.1: Wer im Haushalt hat Ihrer Meinung nach die engste Bindung zu dem/den Hund(en)?**   \| Alle (oder die meisten) im Haushalt \|  \| \| --- \| --- \| \| Ich \|  \| \| Mein(e) Partner/in \|  \| \| Mein Kind/Stiefkind \|  \| \| Meine Kinder/Stiefkinder \|  \| \| Andere \|  \| \| Ich weiß nicht \|  \|   **Instruction A12.2: single-choice question and only if A2=Katze & A10.2 = “ Ich wohne mit (einem/einer) anderen Erwachsenen.” or “ Ich wohne mit (einem/einer) anderen Erwachsenen und einem Kind unter 18 Jahren (ständig).” or “ Ich wohne mit (einem/einer) anderen Erwachsenen und einem Kind unter 18 Jahren (teilweise).“ or “Ich wohne mit (einem/einer) anderen Erwachsenen und Kindern unter 18 Jahren (ständig)“ or “ Ich wohne mit (einem/einer) anderen Erwachsenen und Kindern unter 18 Jahren (teilweise)“ or „Ich wohne mit einem Kind unter 18 Jahren (ständig).“ or „Ich wohne mit einem Kind unter 18 Jahren (teilweise).“ or „Ich wohne mit Kindern unter 18 Jahren (ständig).“ or „Ich wohne mit Kindern unter 18 Jahren (teilweise).“**  **A.12.2: Wer im Haushalt hat Ihrer Meinung nach die engste Bindung zu der/den Katze(n)?**   \| Alle (oder die meisten) im Haushalt \|  \| \| --- \| --- \| \| Ich \|  \| \| Mein(e) Partner/in \|  \| \| Mein Kind/Stiefkind \|  \| \| Meine Kinder/Stiefkinder \|  \| \| Andere \|  \| \| Ich weiß nicht \|  \|   **Instruction A13.1: multiple choice question and only if A2=Hund**  **A.13.1: Warum wurde(n) der/die Hund(e) angeschafft? Kreuzen Sie alle Antworten an, die auf Sie zutreffen.**   \| Zur Gesellschaft (für Menschen) \|  \| \| --- \| --- \| \| Zur Gesellschaft für (ein) andere(s) Haustier(e) \|  \| \| Zur Zucht \|  \| \| Für Wettbewerbe/Ausstellungen \|  \| \| Für Hundesport (Training, Agility, etc.) \|  \| \| Zur sportlichen Betätigung (Spaziergänge) \|  \| \| Als Jagdhund \|  \| \| Zum Schutz von Heim und/oder Eigentum \|  \| \| Andere \|  \| \| Ich weiß nicht \|  \|   **Instruction A13.2: multiple choice question and only if A2=Katze**  **A.13.2: Warum wurde(n) die Katze(n) angeschafft? Kreuzen Sie alle Antworten an, die auf Sie zutreffen.**   \| Zur Gesellschaft (für Menschen) \|  \| \| --- \| --- \| \| Zur Gesellschaft für (ein) andere(s) Haustier(e) \|  \| \| Zur Zucht \|  \| \| Für Wettbewerbe/Ausstellungen \|  \| \| Um Nagetiere vom Haus abzuhalten \|  \| \| Die Katze(n) ist/sind mir zugelaufen und geblieben \|  \| \| Andere \|  \| \| Ich weiß nicht \|  \|   **Instruction 14.1: only if**  **Number of pet species=1 & A2Hund=1**  **Number of pet species=1 & A2Katze=1**  **Number of pet species=1 & A2Pferd=1**  **Number of pet species=1 & or A2Hase/Kaninchen=1**  **Number of pet species=1 & A2Nagetier=1**  **Number of pet species=1 & A2Vogel=1**  **Number of pet species=1 & A2Reptil=1**  **Number of pet species=1 & A2Fische=1**  **Number of pet species=1 & A2** **Anderes Haustier als die Obengenannten=1**  **A14.1: Denken Sie an Ihr Haustier und geben Sie an, ob Sie den folgenden Aussagen gar nicht zustimmen, eher nicht zustimmen, eher zustimmen oder völlig zustimmen.**  *Please use answer options /matrix provided under question A.14.11.1*  **Instruction 14.2: only if Number of pet species=1 & A2Hund>1**  **A14.2: Denken Sie an Ihren Lieblingshund und geben Sie an, ob Sie den folgenden Aussagen gar nicht zustimmen, eher nicht zustimmen, eher zustimmen oder völlig zustimmen.**  *Please use answer options /matrix provided under question A.14.11.1*  **Instruction 14.3: only if Number of pet species=1 & A2CKatze>1**  **A14.3: Denken Sie an Ihre Lieblingskatze und geben Sie an, ob Sie den folgenden Aussagen gar nicht zustimmen, eher nicht zustimmen, eher zustimmen oder völlig zustimmen.**  *Please use answer options /matrix provided under question A.14.11.1*  **Instruction 14.4: only if Number of pet species=1 & A2Pferd>1**  **A14.4: Denken Sie an Ihr Lieblingspferd und geben Sie an, ob Sie den folgenden Aussagen gar nicht zustimmen, eher nicht zustimmen, eher zustimmen oder völlig zustimmen.**  *Please use answer options /matrix provided under question A.14.11.1*  **Instruction 14.5: only if Number of pet species=1 & A2Hasen/Kaninchen>1**  **A14.5: Denken Sie an Ihren Lieblingshasen/Lieblingskaninchen und geben Sie an, ob Sie den folgenden Aussagen gar nicht zustimmen, eher nicht zustimmen, eher zustimmen oder völlig zustimmen.**  *Please use answer options /matrix provided under question A.14.11.1*  **Instruction 14.6: only if Number of pet species=1 & A2Nagetier>1**  **A14.6: Denken Sie an Ihr Lieblingsnagetier und geben Sie an, ob Sie den folgenden Aussagen gar nicht zustimmen, eher nicht zustimmen, eher zustimmen oder völlig zustimmen.**  *Please use answer options /matrix provided under question A.14.11.1*  **Instruction 14.7: only if Number of pet species=1 & A2Vogel>1**  **A14.7: Denken Sie an Ihren Lieblingsvogel und geben Sie an, ob Sie den folgenden Aussagen gar nicht zustimmen, eher nicht zustimmen, eher zustimmen oder völlig zustimmen.**  **Instruction 14.8: only if Number of pet species=1 & A2Reptil>1**  **A14.8: Denken Sie an Ihr Lieblingsreptil und geben Sie an, ob Sie den folgenden Aussagen gar nicht zustimmen, eher nicht zustimmen, eher zustimmen oder völlig zustimmen.**  *Please use answer options /matrix provided under question A.14.11.1*  **Instruction 14.9: only if Number of pet species=1 & A2Fische>1**  **A14.9: Denken Sie an Ihren Lieblingsfisch und geben Sie an, ob Sie den folgenden Aussagen gar nicht zustimmen, eher nicht zustimmen, eher zustimmen oder völlig zustimmen.**  *Please use answer options /matrix provided under question A.14.11.1*  **Instruction 14.10: only if Number of pet species=1 & A2 Anderes Haustier als die Obengenannten** **>1**  **A14.10: Denken Sie an Ihr Lieblingshaustier und geben Sie an, ob Sie den folgenden Aussagen gar nicht zustimmen, eher nicht zustimmen, eher zustimmen oder völlig zustimmen.**  *Please use answer options /matrix provided under question A.14.11.1*  **Instruction A.14.11: only if Number of pet species>1**  **A14.11: Denken Sie nun bitte an Ihr Lieblingshaustier. Was ist Ihr Lieblingshaustier?**   \| Hund \|  \| \| --- \| --- \| \| Katze \|  \| \| Pferd \|  \| \| Hase / Kaninchen \|  \| \| Nagetier (z.B. Hamster, Meerschweinchen, Chinchilla, Maus/Ratte) \|  \| \| Vogel \|  \| \| Reptil (z.B. Eidechse, Schlange, Schildkröte) \|  \| \| Fische (im Aquarium oder in einem Teich im Garten) \|  \| \| Anderes Haustier als die Obengenannten \|  \|   **Instruction 14.11.1: only if Number of pet species>1**  **A14.11.1: Geben Sie nun bitte an, ob Sie den folgenden Aussagen über Ihr Lieblingshaustier gar nicht zustimmen, eher nicht zustimmen, eher zustimmen oder völlig zustimmen.**  *1 = stimme überhaupt nicht zu, 2 = stimme eher nicht zu, 3 = stimme eher zu und 4 = stimme völlig zu*   \|  \| **Bindung zu meinem Haustier** \| **1** \| **2** \| **3** \| **4** \| \| --- \| --- \| --- \| --- \| --- \| --- \| \| 1 \| Mein Haustier bedeutet mir mehr als jeder meiner Freunde. \|  \|  \|  \|  \| \| 2 \| Ich vertraue mich oft meinem Haustier an. \|  \|  \|  \|  \| \| 3 \| Ich finde, dass Haustiere dieselben Rechte und Privilegien haben sollte wie andere Familienmitglieder auch. \|  \|  \|  \|  \| \| 4 \| Ich finde, dass mein Haustier mein bester Freund/meine beste Freundin ist. \|  \|  \|  \|  \| \| 5 \| Meine Gefühle gegenüber Leuten werden häufig davon beeinflusst, wie sich diese meinem Haustier gegenüber verhalten. \|  \|  \|  \|  \| \| 6 \| Ich liebe mein Haustier, weil er/sie mir gegenüber loyaler ist, als die meisten Menschen in meinem Leben. \|  \|  \|  \|  \| \| 7 \| Ich genieße es anderen Menschen Bilder meines Haustieres zu zeigen. \|  \|  \|  \|  \| \| 8 \| Ich finde, mein Haustier ist nur ein Haustier. \|  \|  \|  \|  \| \| 9 \| Ich liebe mein Haustier, weil es mich nie verurteilt. \|  \|  \|  \|  \| \| 10 \| Mein Haustier weiß es, wenn es mir schlecht geht. \|  \|  \|  \|  \| \| 11 \| Ich spreche oft mit anderen Menschen über mein Haustier. \|  \|  \|  \|  \| \| 12 \| Mein Haustier versteht mich. \|  \|  \|  \|  \| \| 13 \| Ich glaube, dass die Liebe zu meinem Haustier mir hilft, gesund zu bleiben. \|  \|  \|  \|  \| \| 14 \| Haustiere verdienen genau so viel Respekt wie Menschen. \|  \|  \|  \|  \| \| 15 \| Mein Haustier und ich haben eine sehr enge Beziehung. \|  \|  \|  \|  \| \| 16 \| Ich würde fast alles tun, um gut für mein Haustier zu sorgen. \|  \|  \|  \|  \| \| 17 \| Ich spiele häufig mit meinem Haustier. \|  \|  \|  \|  \| \| 18 \| Ich finde, dass mein Haustier ein toller Begleiter ist. \|  \|  \|  \|  \| \| 19 \| Mein Haustier macht mich glücklich. \|  \|  \|  \|  \| \| 20 \| Ich finde, dass mein Haustier ein Teil meiner Familie ist. \|  \|  \|  \|  \| \| 21 \| Ich bin meinem Haustier nicht sehr verbunden. \|  \|  \|  \|  \| \| 22 \| Der Besitz eines Haustieres macht mich glücklicher. \|  \|  \|  \|  \| \| 23 \| Für mich ist mein Haustier ein Freund/eine Freundin. \|  \|  \|  \|  \|   **Instruction A15.1: only if A2=Hund**  **A.15.1: Bitte geben Sie an, welche Art von tierärztlicher Praxis Sie üblicherweise mit Ihrem/Ihren Hund(en) besuchen:**   \| Tierarztpraxis (1-3 TierärztInnen) \|  \| \| --- \| --- \| \| Tierarztklinik (4 oder mehr TierärztInnen) \|  \| \| Universitätsklinik \|  \| \| Gemischte Praxis (in der auch andere Tierarten behandelt werden, z. B. Nutztiere). \|  \| \| Wohltätigkeitsorganisation \|  \| \| Ich besuche unterschiedliche Praxistypen. \|  \| \| Ich habe keine Tierarztpraxis, die ich besuche \|  \| \| Ich weiß nicht \|  \|   **Instruction A15.2: only if A2=Katze**  **A.15.2: Bitte geben Sie an, welche Art von tierärztlicher Praxis Sie üblicherweise mit Ihrer/Ihren Katze(n) besuchen:**   \| Tierarztpraxis (1-3 TierärztInnen) \|  \| \| --- \| --- \| \| Tierarztklinik (4 oder mehr TierärztInnen) \|  \| \| Universitätsklinik \|  \| \| Gemischte Praxis (in der auch andere Tierarten behandelt werden, z. B. Nutztiere). \|  \| \| Wohltätigkeitsorganisation \|  \| \| Ich besuche unterschiedliche Praxistypen. \|  \| \| Ich habe keine Tierarztpraxis, die ich besuche \|  \| \| Ich weiß nicht \|  \|   **Instruction A.16: only if A2=Hund &/or A2=Katze**  **A.16: Wenn Sie an Ihre(n) Tierarzt/Tierärztin denken, wie wichtig sind Ihnen die folgenden Aspekte?**  1 = stimme gar nicht zu; 2 = stimme nicht zu; 3 = stimme eher nicht zu; 4 = neutral (weder Zustimmung noch Ablehnung); 5 = stimme eher zu; 6 = stimme zu und 7 = stimme völlig zu; 8 = Ich weiß nicht   \|  \| **Es ist wichtig, dass …** \| 1 \| 2 \| 3 \| 4 \| 5 \| 6 \| 7 \| 8 \| \| --- \| --- \| --- \| --- \| --- \| --- \| --- \| --- \| --- \| --- \| \| 1 \| ich jedes Mal denselben/dieselbe Tierarzt/Tierärztin sehe. \|  \|  \|  \|  \|  \|  \|  \|  \| \| 2 \| ich ein gutes Verhältnis mit meinem/meiner Tierarzt/Tierärztin habe / meine(n) Tierarzt/Tierärztin mag. \|  \|  \|  \|  \|  \|  \|  \|  \| \| 3 \| mein Tier meine(n) Tierarzt/Tierärztin mag / sich mit ihm/ihr wohlfühlt. \|  \|  \|  \|  \|  \|  \|  \|  \| \| 4 \| mein(e) Tierarzt/Tierärztin Interesse für mein Haustier zeigt. \|  \|  \|  \|  \|  \|  \|  \|  \| \| 5 \| mein(e) Tierarzt/Tierärztin Zusatzqualifikationen zusätzlich zur veterinärmedizinischen Ausbildung hat. \|  \|  \|  \|  \|  \|  \|  \|  \| \| 6 \| mein(e) Tierarzt/Tierärztin ein professionelles Auftreten hat. \|  \|  \|  \|  \|  \|  \|  \|  \| \| 7 \| ich meinem/meiner Tierarzt/Tierärztin vertraue. \|  \|  \|  \|  \|  \|  \|  \|  \|   **Instruction A.17: only if A2=Hund &/or A2=Katze**  **A.17: Wie wichtig sind die folgenden Aspekte für Sie bei der Auswahl einer Praxis?**  *1 = „überhaupt nicht wichtig“; 2 = „weniger wichtig“; 3 = „wichtig“; 4 = „sehr wichtig“; 5 = „Ich weiß nicht“*   \|  \| **Die Praxis sollte…** \| **1** \| **2** \| **3** \| **4** \| **5** \| \| --- \| --- \| --- \| --- \| --- \| --- \| --- \| \| 1 \| - nahe an meinem Wohnort sein \|  \|  \|  \|  \|  \| \| 2 \| - 24-Stunden-Betreuung für Routinefälle selbst anbieten (keine Überweisung anderswohin) \|  \|  \|  \|  \|  \| \| 3 \| - gut erreichbar sein und über Parkmöglichkeiten verfügen. \|  \|  \|  \|  \|  \| \| 4 \| - zu günstigen Zeiten Termine anbieten. \|  \|  \|  \|  \|  \| \| 5 \| - Bedürfnisse der verschiedenen Tierarten berücksichtigen (z.B. separate Warteräume für Katzen zur Stressreduktion) \|  \|  \|  \|  \|  \| \| 6 \| - bei Bedarf spezialisierte Behandlungen anbieten, z.B. Gelenkersatz oder Krebsbehandlung \|  \|  \|  \|  \|  \| \| 7 \| - Alternativmedizin anbieten, z.B. Traditionelle Chinesische Medizin, Homöopathie \|  \|  \|  \|  \|  \| \| 8 \| - vernünftige Preise haben \|  \|  \|  \|  \|  \| \| 9 \| - privat betrieben sein \|  \|  \|  \|  \|  \| \| 10 \| - von einer Firma betrieben werden, z.B. AniCura \|  \|  \|  \|  \|  \| \| 11 \| - Hausbesuche anbieten \|  \|  \|  \|  \|  \| \| 12 \| - Teilzahlung ermöglichen \|  \|  \|  \|  \|  \| \| 13 \| - Direktversicherungsansprüche geltend machen \|  \|  \|  \|  \|  \|   **Instruction A.18: multiple-choice question and only if A2=Hund &/or A2=Katze**  **A.18: Welche Diagnose- und Behandlungsmethoden erwarten Sie von der Praxis, die Sie üblicherweise besuchen? Bitte kreuzen Sie alle zutreffenden an.**   \| **Radiografie** (Röntgen) \|  \| \| --- \| --- \| \| **Ultraschall** (bildgebendes Verfahren, das häufig für Bauchraumuntersuchungen eingesetzt wird) \|  \| \| **Endoskopie** (Kamera für einen Blick in das Innere des Körpers, zum Entfernen von Fremdkörpern, z.B. aus dem Magen, den Atemwegen – ermöglicht „Schlüssellochchirurgie“) \|  \| \| **Arthroskopie** (Kamera für den Blick in das Innere von Gelenken) \|  \| \| **Hauseigenes Labor** (z.B. für Blutproben, Urinuntersuchungen) \|  \| \| **Zahnärztliche Ausstattung** \|  \| \| **MRT-Scanner** (hochentwickeltes bildgebendes Verfahren, das häufig an der Wirbelsäule eingesetzt wird) \|  \| \| **CT-Scanner** (hochentwickeltes bildgebendes Verfahren, das häufig im Brustraum eingesetzt wird) \|  \| \| **keines der Obengenannten** \|  \| \| **Ich weiß nicht** \|  \|   **Instruction A.19.1: single-choice question and only if A2=Hund**  **A.19.1: Haben Sie mit Ihrem/Ihren Hund(en) jemals eine(n) spezialisierten Tierarzt/Tierärztin aufgesucht (z.B. NeurologIn, VerhaltensmedizinerIn, OnkologIn)?**   \| Ja \|  \| \| --- \| --- \| \| Nein \|  \| \| Ich weiß nicht \|  \|   **Instruction A.19.2: single-choice question and only if A2=Katze**  **A.19.2: Haben Sie mit Ihrer/Ihren Katze(n) jemals eine(n) spezialisierten Tierarzt/Tierärztin aufgesucht (z.B. NeurologIn, VerhaltensmedizinerIn, OnkologIn)?**   \| Ja \|  \| \| --- \| --- \| \| Nein \|  \| \| Ich weiß nicht \|  \|   **Instruction A.20.1: single-choice question and only if A2=Hund & A.3.1-A.3.4Krankenversicherung=Nein or A.3.1-A3.4Krankenversicherung=nicht mehr**  **A.20.1: Was würden Sie tun, wenn Ihr(e) nicht versicherter/versicherten Hund(e) an einer schweren Krankheit leiden würde(n) und entweder behandelt werden müsste (mit guten Erfolgsaussichten) oder eingeschläfert werden müsste?**  **Instruction A.20.2: single-choice question and only if A2=Katze & A.3.6-A.3.9Health Insurance=No or A.3.6-A3.9Health Insurance=Not anymore**  **A.20.2: Was würden Sie tun, wenn Ihre nicht versicherte(n) Katze(n) an einer schweren Krankheit leiden würde(n) und entweder behandelt werden müsste (mit guten Erfolgsaussichten) oder eingeschläfert werden müsste?**   \| Ich würde um die Einschläferung bitten. \|  \| \| --- \| --- \| \| Ich würde < 100€ für die Behandlung ausgeben. \|  \| \| Ich würde 101–500€ für die Behandlung ausgeben. \|  \| \| Ich würde 501–1000€ für die Behandlung ausgeben. \|  \| \| Ich würde 1001–3000€ für die Behandlung ausgeben. \|  \| \| Ich würde 3001–5000€ für die Behandlung ausgeben. \|  \| \| Ich würde 5001–7999€ für die Behandlung ausgeben. \|  \| \| Ich würde 8000€ oder mehr für die Behandlung ausgeben. \|  \| \| Ich weiß nicht \|  \|   **Instruction A.21.1: single-choice question and only if A2=Hund & A.3.1-A.3.4Krankenversicherung=Ja**  **A.21.1: Was würden Sie tun, wenn Ihr(e) versicherter/versicherten Hund(e) an einer schweren Krankheit leiden würde(n) und entweder behandelt werden müsste (mit guten Erfolgsaussichten) oder eingeschläfert werden müsste?**  **Instruction A.21.2: single-choice question and only if A2=Katze & A.3.6-A.3.9Krankenversicherung=Ja**  **A.21.2: Was würden Sie tun, wenn Ihr(e) versicherte(n) Katze(n) an einer schweren Krankheit leiden würde(n) und entweder behandelt werden müsste (mit guten Erfolgsaussichten) oder eingeschläfert werden müsste?**   \| Ich würde um die Einschläferung bitten. \|  \| \| --- \| --- \| \| Ich würde < 100€ über die Versicherungssumme hinaus ausgeben. \|  \| \| Ich würde 101–500€ über die Versicherungssumme hinaus ausgeben. \|  \| \| Ich würde 501–1000€ über die Versicherungssumme hinaus ausgeben. \|  \| \| Ich würde 1001–3000€ über die Versicherungssumme hinaus ausgeben. \|  \| \| Ich würde 3001–5000€ über die Versicherungssumme hinaus ausgeben. \|  \| \| Ich würde 5001–7999€ über die Versicherungssumme hinaus ausgeben. \|  \| \| Ich würde 8000€ oder mehr über die Versicherungssumme hinaus ausgeben. \|  \| \| Ich weiß nicht \|  \| |
| **Instruction Section B: only if A2=Hund & A2=Katze**  **ABSCHNITT B:**  **Der zweite Teil des Fragebogens beschäftigt sich mit den hochentwickelten Behandlungsmethoden, die in der modernen Tiermedizin zur Verfügung stehen.**  **B.1: Entwicklungen in der Kleintiermedizin**  **B.1: Bitte geben Sie an, inwieweit Sie den folgenden Aussagen zustimmen:**  1 = stimme überhaupt nicht zu; 2 = stimme nicht zu; 3 = stimme eher nicht zu; 4 = neutral (weder Zustimmung noch Ablehnung); 5 = stimme eher zu; 6 = stimme zu und 7 = stimme völlig zu   \|  \|  \| 1 \| 2 \| 3 \| 4 \| 5 \| 6 \| 7 \| \| --- \| --- \| --- \| --- \| --- \| --- \| --- \| --- \| --- \| \| 1 \| Meinem Haustier sollten dieselben **diagnostischen Tests** wie menschlichen Patienten offenstehen. \|  \|  \|  \|  \|  \|  \|  \| \| 2 \| Meinem Haustier sollten dieselben **Behandlungsmöglichkeiten** wie menschlichen Patienten offenstehen. \|  \|  \|  \|  \|  \|  \|  \| \| 3 \| Ich würde mit meinem Haustier an einer wissenschaftlichen Studie teilnehmen, um die Weiterentwicklung der tierärztlichen Versorgung zu unterstützen, solange das Risiko möglicher Komplikationen niedrig ist. \|  \|  \|  \|  \|  \|  \|  \| \| 4 \| Mein(e) Tierarzt/Tierärztin sollte meinem Haustier die modernste Behandlung bieten, die es gibt. \|  \|  \|  \|  \|  \|  \|  \| \| 5 \| Es ist wichtig, dass mein(e) Tierarzt/Tierärztin mit seinem/ihrem Wissen zur Weiterentwicklung der tierärztlichen Versorgung für zukünftige Patienten beiträgt. \|  \|  \|  \|  \|  \|  \|  \| \| 6 \| Die fortschrittliche Behandlung in der modernen Veterinärmedizin geht „zu weit“, indem sie Tieren zu viel zumutet. \|  \|  \|  \|  \|  \|  \|  \| \| 7 \| Die fortschrittliche Behandlung in der modernen Veterinärmedizin ist unnötig – Tiere sollten nicht auf die gleiche Weise behandelt werden wie Menschen. \|  \|  \|  \|  \|  \|  \|  \| |
| **B.2: Faktoren in Bezug auf die Patienten, die KundInnen und das berufliche Umfeld der TierärztInnen**  **B.2: Bitte geben Sie an, inwieweit Sie den folgenden Aussagen – in Bezug auf den Zugang ihres/ihrer Tierarztes/Tierärztin – zustimmen:**  1 = stimme überhaupt nicht zu; 2 = stimme nicht zu; 3 = stimme eher nicht zu; 4 = neutral (weder Zustimmung noch Ablehnung); 5 = stimme eher zu; 6 = stimme zu und 7 = stimme völlig zu   \|  \|  \| 1 \| 2 \| 3 \| 4 \| 5 \| 6 \| 7 \| \| --- \| --- \| --- \| --- \| --- \| --- \| --- \| --- \| --- \| \| 1 \| Bei medizinischen Entscheidungen sollte mein Haustier für meine(n) Tierarzt/Tierärztin die oberste Priorität sein. \|  \|  \|  \|  \|  \|  \|  \| \| 2 \| Wenn mein Haustier unheilbar krank wäre, sollte mein(e) Tierarzt/Tierärztin bereit sein, Palliativversorgung zu leisten, damit ich genug Zeit habe, mich zu verabschieden. \|  \|  \|  \|  \|  \|  \|  \| \| 3 \| Mein(e) Tierarzt/Tierärztin sollte alle infrage kommenden Behandlungsmöglichkeiten mit mir besprechen, aber die Entscheidung liegt bei mir. \|  \|  \|  \|  \|  \|  \|  \| \| 4 \| Bei der Entscheidungsfindung sollte mein(e) Tierarzt/Tierärztin meine emotionalen Belange berücksichtigen. \|  \|  \|  \|  \|  \|  \|  \| \| 5 \| Mein(e) Tierarzt/Tierärztin sollte mich ermutigen, meinem Haustier die bestmögliche Versorgung zu ermöglichen, auch wenn ich dadurch unter Druck gerate, z. B. in zeitlicher oder finanzieller Hinsicht. \|  \|  \|  \|  \|  \|  \|  \| \| 6 \| Bei der Entscheidungsfindung sollte mein(e) Tierarzt/Tierärztin meine persönliche Situation bzw. Anliegen berücksichtigen, auch wenn dies bedeutet, dass ich meinem Tier nicht die bestmögliche Behandlung zukommen lassen kann. \|  \|  \|  \|  \|  \|  \|  \| \| 7 \| Ich möchte, dass mein(e) Tierarzt/Tierärztin nur Entscheidungen trifft, die im besten Interesse meines Tieres sind, auch wenn das bedeutet, dass er/sie mich an eine(n) andere(n) Tierarzt/Tierärztin überweist. \|  \|  \|  \|  \|  \|  \|  \| \| 8 \| Es liegt in der Verantwortung meines/meiner Tierarztes/Tierärztin, die beste Lösung für mein Haustier **und** mich zu finden. \|  \|  \|  \|  \|  \|  \|  \| \| 9 \| Mein(e) Tierarzt/Tierärztin sollte nicht nur professionell arbeiten, sondern mich auch emotional unterstützen. \|  \|  \|  \|  \|  \|  \|  \| \| 10 \| Wenn mein(e) Tierarzt/Tierärztin eine andere Meinung als ich vertritt, sollte er/sie die endgültige Entscheidung mir überlassen. \|  \|  \|  \|  \|  \|  \|  \| \| 11 \| Es ist wichtiger, dass mein(e) Tierarzt/Tierärztin im besten Interesse meines Haustieres handelt, als meine Bedenken (z.B. finanzielle Probleme) zu berücksichtigen. \|  \|  \|  \|  \|  \|  \|  \| \| 12 \| Im Fall von konfliktreichen Entscheidungen, sollte mein(e) Tierarzt/Tierärztin Mitgefühl für meine persönlichen Gefühle zeigen. \|  \|  \|  \|  \|  \|  \|  \| \| 13 \| Nicht mein(e) Tierarzt/Tierärztin sollte die Entscheidungen treffen, sondern ich sollte sie für mein Haustier treffen. \|  \|  \|  \|  \|  \|  \|  \| \| 14 \| Bei der Entscheidungsfindung sollte mein(e) Tierarzt/Tierärztin sich in meine Situation einfühlen. \|  \|  \|  \|  \|  \|  \|  \| \| 15 \| Mein(e) Tierarzt/Tierärztin sollte bereit sein, bei der Patientenversorgung Kompromisse einzugehen, wenn ich mir die bestmögliche/angemessenste Behandlung nicht leisten kann. \|  \|  \|  \|  \|  \|  \|  \| |
| **B.2.1: Tierärztlicher Rat**  **Instruction B.2.1: single-choice question**  **B.2.1: Waren Sie schon einmal in einer Situation, in der Sie die Behandlung Ihres Haustieres gegen den Rat Ihres/Ihrer Tierarztes/Tierärztin fortsetzen wollten?**   \| Ja \|  \| \| --- \| --- \| \| Nein \|  \| \| Ich weiß nicht \|  \|   **Instruction B.2.2: only if B.2.1=Ja**  **B.2.2: Inwieweit haben die folgenden Faktoren Ihren Wunsch beeinflusst, die Behandlung gegen den Rat Ihres/Ihrer Tierarztes/Tierärztin fortzusetzen?**  1 = überhaupt nicht; 2 = sehr wenig; 3 = mäßig; 4 = relativ stark; 5 = sehr stark und 6 = Ich weiß nicht   \|  \|  \| 1 \| 2 \| 3 \| 4 \| 5 \| 6 \| \| --- \| --- \| --- \| --- \| --- \| --- \| --- \| --- \| \| 1 \| Ich kenne mein Haustier besser als mein/meine Tierarzt/Tierärztin. \|  \|  \|  \|  \|  \|  \| \| 2 \| Druck durch eine andere Person (z.B. Familienmitglied, FreundIn) \|  \|  \|  \|  \|  \|  \| \| 3 \| Ich habe medizinische Informationen im Internet gefunden. \|  \|  \|  \|  \|  \|  \| \| 4 \| Ich denke/dachte, es sei im besten Interesse meines Tieres. \|  \|  \|  \|  \|  \|  \| \| 5 \| Ich habe eine positive Erfahrung mit einem anderen Tier gemacht bzw. davon gehört, das dieselbe Behandlung erhalten hat. \|  \|  \|  \|  \|  \|  \| \| 6 \| Ich habe positive Erfahrungen in der Familie oder im Freundeskreis gemacht/gehört, die dieselbe Behandlung durchlaufen haben. \|  \|  \|  \|  \|  \|  \| \| 7 \| Ich habe positive Berichte über diese Behandlung in den Zeitungen und in sozialen Medien gelesen. \|  \|  \|  \|  \|  \|  \| \| 8 \| Ich habe eine zweite Meinung von einem/einer anderen Tierarzt/Tierärztin eingeholt. \|  \|  \|  \|  \|  \|  \| \| 9 \| Mein Tier war versichert. \|  \|  \|  \|  \|  \|  \|   **Instruction B.2.3: single-choice question**  **B.2.3: Waren Sie schon einmal in einer Situation, in der Sie eine Behandlung Ihres Haustieres, die Ihr(e) Tierarzt/Tierärztin empfohlen hat, abgelehnt haben?**   \| Ja \|  \| \| --- \| --- \| \| Nein \|  \| \| Ich weiß nicht \|  \|   **Instruction B.2.4: only if B.2.3=Ja**  **B.2.4: Inwieweit haben die folgenden Faktoren Ihre Entscheidung beeinflusst, eine von Ihrem/Ihrer Tierarzt/Tierärztin empfohlene Behandlung abzulehnen?**  1 = überhaupt nicht; 2 = sehr wenig; 3 = mäßig; 4 = relativ stark; 5 = sehr stark und 6 = Ich weiß nicht   \|  \|  \| 1 \| 2 \| 3 \| 4 \| 5 \| 6 \| \| --- \| --- \| --- \| --- \| --- \| --- \| --- \| --- \| \| 1 \| Ich konnte mir die Behandlung damals nicht leisten. \|  \|  \|  \|  \|  \|  \| \| 2 \| Ich hatte das Gefühl, dass mein Tier zu alt war. \|  \|  \|  \|  \|  \|  \| \| 3 \| Ich hatte keine starke emotionale Bindung zu meinem Tier. \|  \|  \|  \|  \|  \|  \| \| 4 \| Druck durch eine andere Person (z.B. Familienmitglied, FreundIn) \|  \|  \|  \|  \|  \|  \| \| 5 \| Ich habe/hatte nur eingeschränkte Zeitressourcen für die erforderliche Versorgung. \|  \|  \|  \|  \|  \|  \| \| 6 \| Ich war nicht der Meinung, dass die Behandlung im besten Interesse meines Tieres war. \|  \|  \|  \|  \|  \|  \| \| 7 \| Ich habe eine schlechte Erfahrung mit einem anderen Tier gemacht bzw. davon gehört, das dieselbe Behandlung durchlaufen hat. \|  \|  \|  \|  \|  \|  \| \| 8 \| Ich habe schlechte Erfahrungen in der Familie oder im Freundeskreis gemacht bzw. davon gehört, die dieselbe Behandlung durchlaufen haben. \|  \|  \|  \|  \|  \|  \| \| 9 \| Ich habe negative Berichte über diese Behandlung in den Zeitungen und in sozialen Medien gelesen. \|  \|  \|  \|  \|  \|  \| \| 10 \| Ich wollte eine zweite Meinung von einem/einer anderen Tierarzt/Tierärztin einholen. \|  \|  \|  \|  \|  \|  \| |
| **B.3: Krankenversicherung in der tierärztlichen Praxis**  **Instruction B.3.1: multiple choice question and only if A.3.1Krankenversicherung=Ja or A.3.2.1Krankenversicherung=ja or A.3.2.2 Krankenversicherung=ja or A.3.3.1 Krankenversicherung=ja or A3.3.2 Krankenversicherung=ja or A3.3.3 Krankenversicherung=ja or A.3.4.1 Krankenversicherung=ja or A3.4.2 Krankenversicherung=ja or A3.4.3 Krankenversicherung=ja or A3.6 Krankenversicherung=ja or A.3.7.1 Krankenversicherung=ja or A.3.7.2 Krankenversicherung=ja or A.3.8.1 Krankenversicherung=ja or A3.8.2 Krankenversicherung=ja or A3.8.3 Krankenversicherung=ja or A.3.9.1 Krankenversicherung=ja or A3.9.2 Krankenversicherung=ja or A3.9.3 Krankenversicherung=ja**  **B.3.1 Warum haben Sie eine Krankenversicherung für Ihr(e) Haustier(e)? Bitte kreuzen Sie alle Zutreffenden an.**   \| 1 \| Mein(e) Tierarzt/Tierärztin gab mir den Rat, eine Krankenversicherung abzuschließen. \|  \| \| --- \| --- \| --- \| \| 2 \| Bereits frühere Haustiere waren versichert. \|  \| \| 3 \| Ich war einmal in der Situation, dass ich mir die Behandlung nicht leisten konnte. \|  \| \| 4 \| Freunde gaben mir den Rat, eine Krankenversicherung abzuschließen. \|  \| \| 5 \| Ich denke, das gehört als verantwortungsvolle(r) BesitzerIn dazu. \|  \| \| 6 \| Ohne Versicherung könnte ich mir vielleicht die tierärztliche Behandlung nicht leisten. \|  \| \| 7 \| Weil mein Haustier alt ist und einem höheren Krankheitsrisiko ausgesetzt ist. \|  \| \| 8 \| Um im Falle einer Erkrankung meines Haustieres keine schwierigen finanziellen Entscheidungen treffen zu müssen. \|  \| \| 9 \| Als ich mein Haustier bekam, war die Versicherung gratis/sehr günstig dabei (z.B. Welpenpaket). \|  \| \| 10 \| Ich weiß nicht \|  \| \| 11 \| Andere \|  \|   **Instruction B.3.2: multiple choice question and only if A.3.1Krankenversicherung=Nein or A.3.2.1Krankenversicherung=Nein or A.3.2.2 Krankenversicherung=Nein or A.3.3.1 Krankenversicherung=Nein or A3.3.2 Krankenversicherung=Nein or A3.3.3 Krankenversicherung=Nein or A.3.4.1 Krankenversicherung=Nein or A3.4.2 Krankenversicherung=Nein or A3.4.3 Krankenversicherung=Nein or A3.6 Krankenversicherung=Nein or A.3.7.1 Krankenversicherung=Nein or A.3.7.2 Krankenversicherung=Nein or A.3.8.1 Krankenversicherung=Nein or A3.8.2 Krankenversicherung=Nein or A3.8.3 Krankenversicherung=Nein or A.3.9.1 Krankenversicherung=Nein or A3.9.2 Krankenversicherung=Nein or A3.9.3 Krankenversicherung=Nein**  **B.3.2 Warum haben Sie keine Krankenversicherung für Ihr(e) Haustier(e)? Bitte kreuzen Sie alle Zutreffenden an.**   \| 1 \| Ich hatte noch nichts davon gehört. \|  \| \| --- \| --- \| --- \| \| 2 \| Ich vertraue den Versicherungspolicen nicht. \|  \| \| 3 \| Haustierversicherungspolicen sind zu teuer. \|  \| \| 4 \| Mein(e) Tierarzt/Tierärztin gab mir den Rat, keine Krankenversicherung abzuschließen. \|  \| \| 5 \| Die Vorteile sind die Kosten nicht wert. \|  \| \| 6 \| Es war zu schwierig, eine passende Versicherungspolice zu finden. \|  \| \| 7 \| Mein Haustier ist zu alt und somit ist eine Versicherung nicht mehr relevant. \|  \| \| 8 \| Mein Haustier ist zu alt und die Versicherung ist zu teuer geworden. \|  \| \| 9 \| Ich weiß nicht \|  \| \| 10 \| Andere \|  \|   **Instruction B.3.1: multiple choice question and only if A.3.1Krankenversicherung=nicht mehr or A.3.2.1Krankenversicherung=nicht mehr or A.3.2.2 Krankenversicherung=nicht mehr or A.3.3.1 Krankenversicherung=nicht mehr or A3.3.2 Krankenversicherung=nicht mehr or A3.3.3 Krankenversicherung=nicht mehr or A.3.4.1 Krankenversicherung=nicht mehr or A3.4.2 Krankenversicherung=nicht mehr or A3.4.3 Krankenversicherung=nicht mehr or A3.6 Krankenversicherung=nicht mehr or A.3.7.1 Krankenversicherung=nicht mehr or A.3.7.2 Krankenversicherung=nicht mehr or A.3.8.1 Krankenversicherung=nicht mehr or A3.8.2 Krankenversicherung=nicht mehr or A3.8.3 Krankenversicherung=nicht mehr or A.3.9.1 Krankenversicherung=nicht mehr or A3.9.2 Krankenversicherung=nicht mehr or A3.9.3 Krankenversicherung=nicht mehr**  **B.3.3 Warum haben Sie die Krankenversicherung für Ihr(e) Haustier(e) nicht verlängert? Bitte kreuzen Sie alle Zutreffenden an.**   \| 1 \| Die Vorteile sind die Kosten nicht wert. \|  \| \| --- \| --- \| --- \| \| 2 \| Sie wurde zu teuer. \|  \| \| 3 \| Es wurden zu viele Ausschlüsse hinzugefügt. \|  \| \| 4 \| Nicht alle Kosten waren gedeckt. \|  \| \| 5 \| Ich weiß nicht \|  \| \| 6 \| Andere \|  \| |
| **B.4.: Verwendung von sozialen Medien durch KundInnen im Zusammenhang mit der tierärztlichen Praxis**  **Soziale Medien sind eine Form der elektronischen Kommunikation (z.B. Facebook, Twitter, Instagram), durch die NutzerInnen Online-Communities schaffen, um Informationen, Gedanken, persönliche Nachrichten und andere Inhalte (z.B. Videos, Fotos) zu teilen.**  **Instruction B.4.1: multiple choice question**  **B.4.1: Welche Social-Media-Plattformen nutzen Sie beiläufig oder als aktive(r) Nutzer(in)? Bitte kreuzen Sie alle Zutreffenden an.**   \| 1 \| Ich nutze keine Social-Media-Plattformen. \|  \| \| --- \| --- \| --- \| \| 2 \| Facebook \|  \| \| 3 \| Instagram \|  \| \| 4 \| Twitter \|  \| \| 5 \| YouTube \|  \| \| 6 \| TikTok \|  \| \| 7 \| Andere \|  \|   **Instruction B.4.2: single-choice choice question and only if B4.1=Facebook, B4.1=Instagram, B4.1=Twitter, B4.1=YouTube, B4.1=TikTok and/or B4.1=Andere**  **B.4.2: Wie oft nutzen Sie Social-Media-Plattformen?**   \| Täglich \|  \| \| --- \| --- \| \| 4-6 Tage in der Woche \|  \| \| 1-3 Tage in der Woche \|  \| \| Weniger als einen Tag in der Woche \|  \| \| Seltener \|  \| \| Ich weiß nicht \|  \|   **Instruction B.4.3: single-choice choice question**  **B.4.3: Erwarten Sie, dass Ihre Tierarztpraxis eine aktive Social-Media-Präsenz hat?**   \| Ja \|  \| \| --- \| --- \| \| Nein \|  \| \| Ich weiß nicht \|  \|   **B.4.4: Inwieweit stimmen Sie den folgenden Aussagen zu?**  1 = stimme überhaupt nicht zu; 2 = stimme nicht zu; 3 = stimme eher nicht zu; 4 = neutral (weder Zustimmung noch Ablehnung); 5 = stimme eher zu; 6 = stimme zu, 7 = stimme völlig zu und 8 = Ich weiß nicht   \|  \| **Ich denke, dass die Verwendung sozialer Medien durch Tierarztpraxen…** \| 1 \| 2 \| 3 \| 4 \| 5 \| 6 \| 7 \| 8 \| \| --- \| --- \| --- \| --- \| --- \| --- \| --- \| --- \| --- \| --- \| \| 1 \| den Menschen einen Einblick gibt, was in der Praxis passiert. \|  \|  \|  \|  \|  \|  \|  \|  \| \| 2 \| nicht notwendig ist. \|  \|  \|  \|  \|  \|  \|  \|  \| \| 3 \| ein nützlicher Weg für TierbesitzerInnen ist, um eine (neue) Praxis zu finden. \|  \|  \|  \|  \|  \|  \|  \|  \| \| 4 \| es den TierbesitzerInnen ermöglicht, mit der Tierarztpraxis und/oder dem/der Tierarzt/Tierärztin auf informelle und einfache Weise in Kontakt zu bleiben. \|  \|  \|  \|  \|  \|  \|  \|  \|   **Instruction B.4.5: single-choice choice question**  **B.4.5: Haben Sie im Internet (z.B. Website oder soziale Medien) einmal (einen) negative(n) Kommentar(e) oder (eine) Beschwerde(n) über Ihre(n) Tierarzt/Tierärztin gepostet?**   \| Ja \|  \| \| --- \| --- \| \| Nein \|  \|   **Instruction B.4.6: multiple choice question and only if B.4.5=yes**  **B.4.6: Falls ja, was war das Thema des/der negativen Kommentars/Kommentare oder der Beschwerde(n)? Bitte kreuzen Sie alle Zutreffenden an.**   \| zu lange Wartezeit \|  \| \| --- \| --- \| \| der medizinische Rat des/der Tierarztes/Tierärztin \|  \| \| der Umgang des Personals mit meinem Haustier \|  \| \| das Ergebnis der Behandlung meines Hautieres / Komplikationen \|  \| \| die Kosten der Behandlung \|  \| \| das Verhalten (eines/einer) bestimmen/bestimmter Mitarbeiter(s)/Mitarbeiterin(nen) \|  \| \| fehlende technische Ausstattung der Praxis \|  \| \| unfreundliche Kommunikation des/der Tierarztes/Tierärztin \|  \| \| zu technische oder komplizierte Erklärungen des/der Tierarztes/Tierärztin \|  \| \| unfreundliche Kommunikation anderer MitarbeiterInnen \|  \| \| Andere \|  \|   **Instruction B.4.7: single-choice choice question**  **B.4.7: Hat Ihr(e) Tierarzt/Tierärztin auf das negative Feedback / die Beschwerde reagiert?**   \| Ja \|  \| \| --- \| --- \| \| Nein \|  \| \| Ich weiß nicht \|  \| |
| **B.5: Nutzung von Internetquellen für veterinärmedizinische Informationen**  **Instruction B.5.1: single-choice choice question**  **B.5.1: Wie oft nutzen Sie Internetquellen, um medizinische Informationen zu finden, BEVOR Sie Ihre(n) Tierarzt/Tierärztin aufsuchen?**   \| Nie \|  \| \| --- \| --- \| \| Gelegentlich \|  \| \| Häufig \|  \| \| Immer \|  \| \| Ich weiß nicht \|  \|   **Instruction B.5.2: single-choice choice question**  **B.5.2: Wie oft nutzen Sie Internetquellen, um medizinische Informationen zu finden, NACH dem Gespräch mit Ihrem/Ihrer Tierarzt/Tierärztin?**   \| Nie \|  \| \| --- \| --- \| \| Gelegentlich \|  \| \| Häufig \|  \| \| Immer \|  \| \| Ich weiß nicht \|  \|   **Instruction B.5.3: multiple choice question and only if B.5.1 or B.5.2=gelegentlich, B.5.1 or B.5.2=häufig, B.5.1 or B.5.2=immer and/or B.5.1 or B.5.2=Ich weiß nicht**  **B.5.3: Welche Quellen haben Sie verwendet?**   \| 1 \| Blogs und Chatrooms \|  \| \| --- \| --- \| --- \| \| 2 \| Websites mit veterinärmedizinischen Inhalten \|  \| \| 3 \| Soziale Medien (z.B. Facebook, Twitter) \|  \| \| 4 \| Website der Praxis \|  \| \| 5 \| Tierärztlichen Verbänden \|  \| \| 6 \| Website einer Universität \|  \| \| 7 \| Andere \|  \|   **Instruction B.5.4: only if B.5.1 or B.5.2=gelegentlich, B.5.1 or B.5.2=häufig, B.5.1 or B.5.2=immer and/or B.5.1 or B.5.2=Ich weiß nicht**  **B.5.4: Inwieweit stimmen Sie den folgenden Aussagen zu?**  1 = stimme überhaupt nicht zu; 2 = stimme nicht zu; 3 = stimme eher nicht zu; 4 = neutral (weder Zustimmung noch Ablehnung); 5 = stimme eher zu; 6 = stimme zu; 7 = stimme völlig zu und 8 = Ich weiß nicht   \|  \| **Die Verwendung von Internetquellen …** \| 1 \| 2 \| 3 \| 4 \| 5 \| 6 \| 7 \| 8 \| \| --- \| --- \| --- \| --- \| --- \| --- \| --- \| --- \| --- \| --- \| \| 1 \| erhöht meine Erwartungen an den Standard der verfügbaren tierärztlichen Versorgung für mein Haustier. \|  \|  \|  \|  \|  \|  \|  \|  \| \| 2 \| ermöglicht mir ein informierteres Gespräch mit meinem/meiner Tierarzt/Tierärztin. \|  \|  \|  \|  \|  \|  \|  \|  \| \| 3 \| kann zu Situationen führen, in denen ich besser informiert bin als mein(e) Tierarzt/Tierärztin. \|  \|  \|  \|  \|  \|  \|  \|  \| \| 4 \| ermöglicht es mir, meine(n) Tierarzt/Tierärztin herauszufordern, seine/ihre Empfehlung zu begründen. \|  \|  \|  \|  \|  \|  \|  \|  \| \| 5 \| hilft mir dabei, die richtige Entscheidung für mein Tier zu treffen. \|  \|  \|  \|  \|  \|  \|  \|  \| \| 6 \| ermöglicht es mir, einige Medikamente billiger zu kaufen (z.B. Flohmittel, Entwurmungsmittel). \|  \|  \|  \|  \|  \|  \|  \|  \| \| 7 \| kann einen falschen Eindruck von der Standardbehandlung vermitteln. \|  \|  \|  \|  \|  \|  \|  \|  \|   **Instruction B.5.5: single-choice question and only if B.5.1 or B.5.2=gelegentlich, B.5.1 or B.5.2=häufig, B.5.1 or B.5.2=immer and/or B.5.1 or B.5.2=Ich weiß nicht**  **B.5.5: Sind Sie durch im Internet gefundene Informationen jemals zu einer anderen Meinung als dem professionellen Rat Ihres/Ihrer Tierarztes/Tierärztin gekommen?**   \| Ja \|  \| \| --- \| --- \| \| Nein \|  \| \| Ich weiß nicht \|  \| |
| **B.6: Telemedizin in der modernen Kleintierpraxis**  **Die Telemedizin ist zu einer Option in der modernen Kleintierpraxis geworden, bei der durch den Einsatz von elektronischen Medien eine Beratung und Betreuung "aus der Ferne" möglich ist. Dies erlaubt es TierbesitzerInnen, eine umfassende und kostenpflichtige Beratung durch TierärztInnen in Anspruch zu nehmen, ohne dass sie Ihre Tiere in die Praxis bringen müssen.**  **Instruction B.6.1: single-choice question**  **B.6.1: Haben Sie jemals Telemedizin in Anspruch genommen, um medizinischen Rat für Ihr(e) Haustier(e) einzuholen?**   \| Ja \|  \| \| --- \| --- \| \| Nein \|  \| \| Ich wusste nicht, dass das eine Option ist. \|  \|     **Instruction B.6.2: single-choice question and only if B.6.1=Nein or B.6.1=Ich wusste nicht, dass das eine Option ist.**  **B.6.2: Würden Sie von Telemedizin Gebrauch machen, wenn Ihr(e) Tierarzt/Tierärztin dies statt einer persönlichen Beratung anbieten würde?**   \| Ja \|  \| \| --- \| --- \| \| Nein \|  \| \| Ich weiß nicht \|  \|   **B.6.3: Inwieweit stimmen Sie den folgenden Aussagen zu?**  1 = stimme überhaupt nicht zu; 2 = stimme nicht zu; 3 = stimme eher nicht zu; 4 = neutral (weder Zustimmung noch Ablehnung); 5 = stimme eher zu; 6 = stimme zu; 7 = stimme völlig zu und 8 = Ich weiß nicht   \|  \| **Der Einsatz von Telemedizin…** \| 1 \| 2 \| 3 \| 4 \| 5 \| 6 \| 7 \| 8 \| \| --- \| --- \| --- \| --- \| --- \| --- \| --- \| --- \| --- \| --- \| \| 1 \| könnte hilfreich sein, weil ich die Fahrt zum/zur Tierarzt/Tierärztin schwierig finde. \|  \|  \|  \|  \|  \|  \|  \|  \| \| 2 \| könnte meinem Haustier die stressige Fahrt zum/zur Tierarzt/Tierärztin ersparen. \|  \|  \|  \|  \|  \|  \|  \|  \| \| 3 \| könnte mir bei der Entscheidung helfen, ob ein Besuch beim Tierarzt/ bei der Tierärztin notwendig ist. \|  \|  \|  \|  \|  \|  \|  \|  \| \| 4 \| könnte für Folgetermine eine Hilfe sein. \|  \|  \|  \|  \|  \|  \|  \|  \| \| 5 \| könnte mir besseren Zugang zu einem Spezialisten verschaffen, wenn es in der Region keinen gibt. \|  \|  \|  \|  \|  \|  \|  \|  \| \| 6 \| ist gut bei Notfällen. \|  \|  \|  \|  \|  \|  \|  \|  \| \| 7 \| schwächt das Verhältnis zwischen Tierarzt/Tierärztin und Kunden/Kundin. \|  \|  \|  \|  \|  \|  \|  \|  \| \| 8 \| stärkt das Verhältnis zwischen Tierarzt/Tierärztin und Kunden/Kundin. \|  \|  \|  \|  \|  \|  \|  \|  \| \| 9 \| sollte kostengünstiger als eine normale Beratung sein. \|  \|  \|  \|  \|  \|  \|  \|  \| \| 10 \| bietet keine Vorteile. \|  \|  \|  \|  \|  \|  \|  \|  \| \| 11 \| erhöht das Risiko, dass etwas übersehen wird, weil das Tier nicht physisch vom/von der Tierarzt/Tierärztin untersucht wird. \|  \|  \|  \|  \|  \|  \|  \|  \| \| 12 \| ist komfortabler als eine persönliche Beratung. \|  \|  \|  \|  \|  \|  \|  \|  \| \| 13 \| ist für mich keine Option, weil ich die nötigen IT-Kenntnisse oder das benötigte Equipment nicht habe. \|  \|  \|  \|  \|  \|  \|  \|  \| |
| **C: Soziale Unterstützung**  **Als letzten Punkt bitten wir Sie um Angaben zu Ihrem sozialen Umfeld und Ihrem Unterstützungsnetzwerk.**  **C.1: Wie häufig steht Ihnen folgende Unterstützung durch andere Menschen zur Verfügung?**  *1 = nie, 2 = gelegentlich, 3 = meistens, 4 = immer*   \|  \| \| **1** \| **2** \| **3** \| **4** \| \| --- \| --- \| --- \| --- \| --- \| --- \| \| 1 \| Jemand, der Sie zum Arzt fährt, wenn es nötig ist \|  \|  \|  \|  \| \| 2 \| Jemand, der Ihnen Essen zubereitet, wenn Sie dazu nicht in der Lage sind \|  \|  \|  \|  \| \| 3 \| Jemand, der Ihnen bei alltäglichen Arbeiten hilft, wenn Sie krank sind \|  \|  \|  \|  \| \| 4 \| Jemand, der Ihnen in schwierigen Situationen gute Ratschläge gibt \|  \|  \|  \|  \| \| 5 \| Jemand, dem Sie sich anvertrauen oder mit dem Sie über persönliche Probleme sprechen können \|  \|  \|  \|  \| \| 6 \| Jemand, der Ihre Probleme versteht \|  \|  \|  \|  \|   **Instruction C.2: single-choice question and list in drop-down menu**  **C.2: Wie oft haben Sie das Gefühl, niemanden zu haben, mit dem Sie reden können?**   \| Selten oder nie \|  \| \| --- \| --- \| \| Manchmal \|  \| \| Oft \|  \|   **Instruction C.3: single-choice question and list in drop-down menu**  **C.3: Wie oft fühlen Sie sich ausgeschlossen?**   \| Selten oder nie \|  \| \| --- \| --- \| \| Manchmal \|  \| \| Oft \|  \|   **Instruction C.4: single-choice question and list in drop-down menu**  **C.4: Wie oft fühlen Sie sich von anderen isoliert?**   \| Selten oder nie \|  \| \| --- \| --- \| \| Manchmal \|  \| \| Oft \|  \|   **Instruction C.5: single-choice question and list in drop-down menu**  **C.5: Wie oft fühlen Sie sich allein?**   \| Oft / immer \|  \| \| --- \| --- \| \| Manchmal \|  \| \| Gelegentlich \|  \| \| Fast nie \|  \| \| Nie \|  \| |
|  |
| **Herzlichen Dank für Ihre Teilnahme an dieser Umfrage!** |
