# Supplementary material for: The development of a brief version of the Lexington Attachment to Pets Scale (Brief-LAPS)
Source: Front Vet Sci. 2025 Sep 2;12:1619187. doi: 10.3389/fvets.2025.1619187 (PMC12439530; doi:10.3389/fvets.2025.1619187)
Supplement: Supplementary file 4 [file Supplementary_file_4.docx]

**Supplementary file 4**

**Three steps of measurement invariance**

Configural invariance is the first step of measurement invariance. It examines whether the factorial structure is the same across groups. If this is affirmed, then the latent structure is similar in the sense that the groups understand the underlying construct in the same way. The next step, metric invariance, examines whether the different groups respond to the items included in the latent scale in a similar way. This invariance level is tested by restraining factor loadings to be the same across the groups. Finally, scalar invariance examines whether observed scores on the items of the scale are consistently connected with the latent scores across groups. This invariance level is necessary in order to perform unbiased group mean comparisons of the latent construct, and it is tested by constraining factor loadings and item thresholds to be equal across groups (1,2). Also, at this level it is justifiable to use the composite score method (i.e. all observed item scores are summed together) in group mean comparisons (Steinmetz, 2013; Steenkamp & Baumgartner, 1998). As clarified in the main manuscript, we attempt to obtain scalar invariance, as scholars predominantly use the composite score method (see **Table 4** of the paper).

**Table S4.1** below outlines the measurement invariance results. RMSEA and CFI values are reported for each sequential step, starting with factorial invariance, followed by metric invariance, and ending with scalar invariance. If the criteria for metric and scalar invariance were not fulfilled, we released restrictions with a view to identifying partial metric or partial scalar invariance (Putnick & Bornstein, 2016).

We followed guidelines for ordered categorical MG-CFA (Rutkowski & Svetina (2017) that the root mean square error approximation (RMSEA) must not exceed 0.055 in any of the three invariance levels; that the change in the value of the comparative fit index (CFI) must not exceed ΔCFI -0.004 when comparing the metric with the configural model, and when comparing the scalar with the metric model; and that the RMSEA must not exceed ΔRMSEA 0.005 when comparing the metric with the configural model, and must not exceed ΔRMSEA 0.01 when comparing the scalar model with the metric model.

**Variables where sub-groups are compared in MG-CFA**

Species of pet (0=dog; 1=cat)

Country (1=Austria; Denmark=2; UK=3)

Gender (0=male; 1=female): Respondents had two more gender response options available (“Other” and “I do not wish to tell”). But they were treated as missing, as the options were used very infrequently.

Age in years (0=18-47 years; 1> 47 years)

One adult in household (0=two or more adult; 1=one adult)

Child(ren) in family (0=no child(ren); 1=1 or several children)

Income: Respondents were asked about the household’s annual income in the relevant valutas of each country. For each country, we divided annual income into five equally sized groups. These five were subsequently coded into a binary variable (0=low income (income levels 1,2,3 and “I don’t know/do not wish to disclose); 1=High income (income levels 4 and 5)).

UCLA 3-item loneliness scale: This 3-item scale measures loneliness (4). It was recoded into a binary variable (0=scores 3-6; 1=scores 7-9).

Perceived loneliness: this single-item measure is used in national surveys in the UK ([Community Life Survey, England 2017 to 2018: Statistical bulletin](https://assets.publishing.service.gov.uk/media/5b76b131ed915d14f4404b75/Community_Life_Survey_2017-18_statistical_bulletin.pdf)) and the Nordic/Baltic countries (5) as a brief measure of perceived loneliness, i.e. whether a person feels lonely. It is a frequency scale from “often/always” to “never”. We recoded it into a binary variable (1= often/always; 0=all other responses).

Emotional support: This is based on a 6-item measure of social support that covers two dimensions, namely emotional and tangible support (6). We calculated composite scores for both dimensions. Emotional support was recoded into a binary variable (0=scores 3-6; 1=scores 7-12)

Tangible support: This is the second dimension from the 6-item measure of social support that was described about (6). Similar to Emotional support, Tangible support was recoded into a binary variable (0=scores 3-6; 1=scores 7-12)

**Results from tests of measurement invariance**

| **Table S4.1.** Results from MG-CFA of overall fit statistics and incremental changes (n=2037) | | | | | | | | | | |
| --- | --- | --- | --- | --- | --- | --- | --- | --- | --- | --- |
| Model | N | *X*^2^ | df | p-value | ∆ *X*^2^(df) | p-value | CFI | ∆CFI | RMSEA | ∆RMSEA |
| **Animal species of favorite pet** | | | | | | | | | | |
| Configural | 2037 | 87.569 | 28 | <0.001 |  |  | 0.996 |  | 0.046 |  |
| Metric | 2037 | 105.823 | 34 | <0.001 | 24.324 (6) | 0.0005 | 0.995 | -0.001 | 0.046 | 0.000 |
| Scalar | 2037 | 175.756 | 54 | <0.001 | 73.584 (20) | <0.001 | 0.991 | -0.004 | 0.047 | 0.001 |
| **Country** | | | | | | | | | | |
| Configural | 2037 | 107.614 | 42 | <0.001 |  |  | 0.996 |  | 0.048 |  |
| Metric | 2037 | 124.727 | 54 | <0.001 | 30.808 (12) | 0.0001 | 0.995 | -0.001 | 0.044 | -0.004 |
| Scalar | 2037 | 388.672 | 94 | <0.001 | 279.718 (40) | <0.001 | 0.980 | -0.015 | 0.068 | 0.024 |
| Partial scalar* | 2037 | 228.214 | 78 | <0.001 | 112.924(24) | <0.001 | 0.990 | 0.010 | 0.053 | 0.009 |
| **Gender** | | | | | | | | | | |
| Configural | 2037 | 102.028 | 28 | <0.001 |  |  | 0.995 |  | 0.051 |  |
| Metric | 2037 | 150.776 | 34 | <0.001 | 4.269 (6) | 0.6404 | 0.997 | 0.02 | 0.035 | -0.016 |
| Scalar | 2037 | 114.865 | 54 | <0.001 | 41.411 (20) | 0.0033 | 0.996 | -0.01 | 0.033 | -0.002 |
| **Age** | | | | | | | | | | |
| Configural | 2037 | 86.763 | 28 | <0.001 |  |  | 0.996 |  | 0.045 |  |
| Metric | 2037 | 82.951 | 34 | <0.001 | 13.165 (6) | 0.0405 | 0.997 | -0.001 | 0.038 | -0.007 |
| Scalar | 2037 | 139.186 | 54 | <0.001 | 58.945 (20) | 0.0003 | 0.994 | -0.003 | 0.039 | 0.001 |
| **Income** | | | | | | | | | | |
| Configural | 2037 | 83.505 | 28 | <0.001 |  |  | 0.996 |  | 0.044 |  |
| Metric | 2037 | 76.324 | 34 | <0.001 | 9.135 (6) | 0.1661 | 0.997 | 0.001 | 0.035 | -0.009 |
| Scalar | 2037 | 88.690 | 54 | <0.001 | 15.399 (20) | 0.7531 | 0.998 | 0.001 | 0.025 | -0.010 |
| **One adult in household** | | | | | | | | | | |
| Configural | 2037 | 88.121 | 28 | <0.001 |  |  | 0.996 |  | 0.046 |  |
| Metric | 2037 | 115.290 | 34 | <0.001 | 29.059 (6) | <0.001 | 0.994 | -0.002 | 0.048 | 0.002 |
| Scalar | 2037 | 136.317 | 54 | <0.001 | 25.254 (20) | 0.1919 | 0.994 | 0.000 | 0.039 | -0.009 |
| **Children in household** | | | | | | | | | | |
| Configural | 2037 | 96.785 | 28 | <0.001 |  |  | 0.995 |  | 0.049 |  |
| Metric | 2037 | 116.519 | 34 | <0.001 | 30.333 (6) | <0.001 | 0.995 | 0.000 | 0.049 | 0.000 |
| Scalar | 2037 | 154.667 | 54 | <0.001 | 27.143 (20) | 0.1313 | 0.993 | -0.002 | 0.043 | -0.006 |
| **Perceived loneliness** | | | | | | | | | | |
| Configural | 2037 | 104.675 | 28 | <0.001 |  |  | 0.994 |  | 0.052 |  |
| Metric | 2037 | 101.787 | 34 | <0.001 | 13.863 (6) | 0.0312 | 0.995 | 0.001 | 0.044 | -0.006 |
| Scalar | 2037 | 141.489 | 54 | <0.001 | 46.849 (20) | 0.0006 | 0.993 | -0.002 | 0.040 | -0.004 |
| **UCLA loneliness** | | | | | | | | | | |
| Configural | 2037 | 82.407 | 28 | <0.001 |  |  | 0.996 |  | 0.044 |  |
| Metric | 2037 | 83.316 | 34 | <0.001 | 13.894 (6) | 0.0308 | 0.996 | 0.000 | 0.038 | -0.006 |
| Scalar | 2037 | 124.720 | 54 | <0.001 | 46.138 (20) | 0.0008 | 0.995 | -0.001 | 0.036 | -0.002 |
| **Tangible support** | | | | | | | | | | |
| Configural | 2037 | 95.996 | 28 | <0.001 |  |  | 0.995 |  | 0.044 |  |
| Metric | 2037 | 94.329 | 34 | <0.001 | 15.292 (6) | 0.0181 | 0.996 | 0.001 | 0.044 | 0.000 |
| Scalar | 2037 | 122.201 | 54 | <0.001 | 31.361 (6) | 0.0506 | 0.995 | -0.001 | 0.035 | -0.009 |
| **Emotional support** | | | | | | | | | | |
| Configural | 2037 | 93.765 | 28 | <0.001 |  |  | 0.995 |  | 0.048 |  |
| Metric | 2037 | 111.070 | 34 | <0.001 | 24.278 (6) | 0.0005 | 0.995 | 0.000 | 0.047 | -0.002 |
| Scalar | 2037 | 167.436 | 54 | <0.001 | 60.815 (20) | 0.0000 | 0.992 | -0.003 | 0.045 | -0.002 |
| CFI = comparative fit index;  RMSEA = root mean square error of approximation;  p-value for ∆*X*^2^ was calculated using the DIFFTEST function in *MPlus* version 8.6;  * Partial scalar invariance was reached after freeing thresholds 1-3 for the item “My pet knows when I’m feeling bad”, freeing thresholds 1-3 for the item “My pet means more to me than any of my friends”, and freeing thresholds 1 and 2 for the item “I love my pet because it never judges me” | | | | | | | | | | |

**References:**

1. Meredith W. Measurement invariance, factor analysis and factorial invariance. Psychometrika. 1993 Dec;58(4):525–43.

2. Steenkamp JEM, Baumgartner H. Assessing Measurement Invariance in Cross‐National Consumer Research. J CONSUM RES. 1998 Jun;25(1):78–107.

3. Steinmetz H. Analyzing Observed Composite Differences Across Groups: Is Partial Measurement Invariance Enough? Methodology. 2013 Jan 1;9(1):1–12.

4. Hughes ME, Waite LJ, Hawkley LC, Cacioppo JT. A Short Scale for Measuring Loneliness in Large Surveys: Results From Two Population-Based Studies. Res Aging. 2004 Nov;26(6):655–72.

5. Reine I, Miķelsone M, Tomsone S, Guðmundsson H, Ivanovs A, Sigurður Guðmundsson H, et al. Loneliness in the Baltic-Nordic Region. In: Zahir Ahmed M, editor. Determinants of Loneliness [Internet]. IntechOpen; 2024 [cited 2024 Sep 16]. Available from: https://www.intechopen.com/chapters/1173304

6. Beutel ME, Brähler E, Wiltink J, Michal M, Klein EM, Jünger C, et al. Emotional and tangible social support in a German population-based sample: Development and validation of the Brief Social Support Scale (BS6). Kocalevent RD, editor. PLoS ONE. 2017 Oct 12;12(10):e0186516.
